# Novel Cytotoxic Sesquiterpene Coumarin Ethers and Sulfur-Containing Compounds from the Roots of *Ferula turcica*

**DOI:** 10.3390/molecules28155733

**Published:** 2023-07-28

**Authors:** Fatma Memnune Eruçar, Sarath P. D. Senadeera, Jennifer A. Wilson, Ekaterina Goncharova, John A. Beutler, Mahmut Miski

**Affiliations:** 1 Department of Pharmacognosy, Faculty of Pharmacy, Istanbul University, Istanbul 34116, Türkiye; memnune.erucar@istanbul.edu.tr; 2 Department of Pharmacognosy, Institute of Graduate Studies in Health Sciences, Istanbul University, Istanbul 34116, Türkiye; 3 Molecular Targets Program, National Institutes of Health, National Cancer Institute, Frederick, MD 21702, USA; dayani.sarathparakumge@nih.gov (S.P.D.S.); wilsonje@mail.nih.gov (J.A.W.); katya.goncharova@nih.gov (E.G.); 4 Advanced Biomedical Computational Science, Frederick National Laboratory for Cancer Research, Frederick, MD 21702, USA

**Keywords:** Apiaceae, *Ferula turcica*, sesquiterpene coumarin ethers, sulfur-containing compounds, cytotoxic activity, colon cancer, kidney cancer

## Abstract

Six new sesquiterpene coumarin ethers, namely turcicanol A (**1**), turcicanol A acetate (**2**), turcicanol B (**3**), turcica ketone (**4**), 11′-dehydrokaratavicinol (**5**), and galbanaldehyde (**6**), and one new sulfur-containing compound, namely turcicasulphide (**7**), along with thirty-two known secondary metabolites were isolated from the root of the endemic species *Ferula turcica* Akalın, Miski, & Tuncay through a bioassay-guided isolation approach. The structures of the new compounds were elucidated by spectroscopic analysis and comparison with the literature. Cell growth inhibition of colon cancer cell lines (COLO205 and HCT116) and kidney cancer cell lines (UO31 and A498) was used to guide isolation. Seventeen of the compounds showed significant activity against the cell lines.

## 1. Introduction

Cancers are rapidly increasing in incidence worldwide and, in total, are the second most important cause of death worldwide. According to research by the World Health Organization (WHO), cancer was the cause of death of 10 million people in 2020 [1]. Cancer is still an incurable disease; thus, there is a need to find new molecules in this field. Türkiye is one of the leading countries in its herbal richness, biodiversity, and ethnobotanical knowledge [2,3]. An important source that inspires researchers in the discovery of pharmaceuticals for the treatment of many diseases is the use of botanical resources. According to studies, 81% of cancer drugs approved between 1940 and 2014 are compounds of natural origin [4].

The genus *Ferula* is one of the largest genera of the Apiaceae family and ranks third in the world and first in Asia, with approximately 185 species [5]. About 26 species of *Ferula* grow in Türkiye (Iran-Turan region), 16 of which are endemic, and they are commonly referred to as “Çaksir” or “Çasir” [6,7]. *Ferula turcica* Akalın, Miski, & Tuncay is a new species defined as a member of the section *Merwia* in Türkiye [6]. The use of gum-like resins (oleo–gum–resin) obtained from *Ferula* species for the treatment of several diseases, including cancer, for thousands of years has been recorded in various sources, including Dioscorides’ *De Materia Medica* and Avicenna’s *The Canon of Medicine* [8,9,10,11]. The compounds identified in *Ferula* species and frequently encountered in gum–resin drugs obtained from *Ferula* species are mostly sesquiterpene esters [12,13,14], sesquiterpene coumarin ethers [15,16], and sulfur-containing substances [17,18].

Studies with sesquiterpene coumarin ethers have shown that these secondary metabolites have cytotoxic activity; they induce apoptosis in Jurkat-derived apoptotic cells and contribute to tumor suppression by inhibiting macrophage secretion and facilitating beneficial phenotypes [19,20,21]. Due to the high affinity of sesquiterpene coumarins such as conferone toward the -p-glycoprotein (Pgp) transporter, conferone has a synergistic effect on the cytotoxic activity of cancer drugs, such as vinblastine, whose effectiveness is reduced in the treatment of cancer [22]. Therefore, sesquiterpene coumarins constitute an important group for promising new drug discovery in the field of cancer.

In this study, the dichloromethane extract of *Ferula turcica* roots belonging to the *Merwia* section of the *Ferula* species in Türkiye was investigated for its cytotoxic secondary metabolites.

## 2. Results

**Figure 1:**
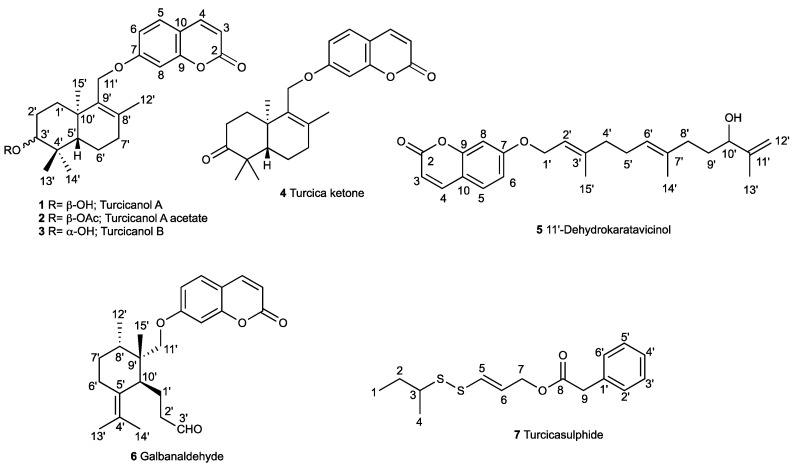
New compounds isolated from *Ferula turcica* roots.

The dichloromethane and methanol extracts of the roots of *Ferula turcica* were tested against COLO205 (colon), HCT116 (colon), UO31 (kidney), and A498 (kidney) cancer cell lines. Comparison of the cytotoxic activity of dichloromethane and methanol extracts of the roots of *F. turcica* showed that the cytotoxic constituents were mainly concentrated in the dichloromethane extract (Table 1). The dichloromethane extract of the roots of *F. turcica* was subjected to Sephadex LH-20 fractionation, followed by preparative HPLC with reverse-phase C18 columns to yield 7 novel (Figure 1) and 30 known compounds.

### 2.1. Characterization of Cytotoxic Compounds

Six new sesquiterpene coumarin ethers, namely turcicanol A (**1**), turcicanol A acetate (**2**), turcicanol B (**3**), turcica ketone (**4**), 11′-dehydrokaratavicinol (**5**), and galbanaldehyde (**6**), and a new sulfur-containing compound, namely turcicasulphide (**7**), were isolated from the dichloromethane extract of the roots of *Ferula turcica* (Figure 1).

**Figure 2:**
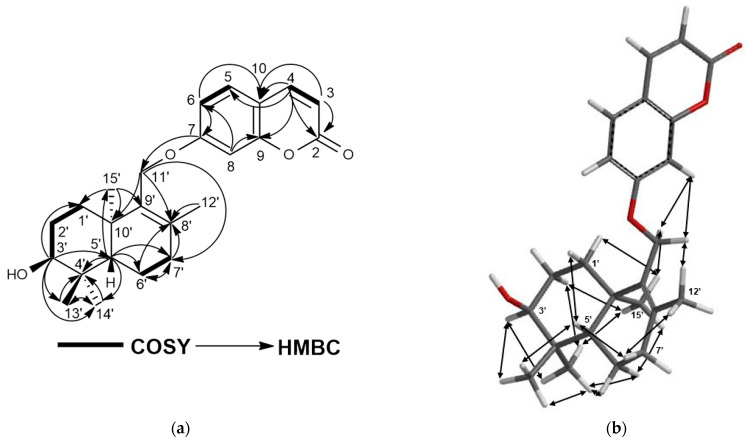
(**a**) COSY and HMBC correlations of turcicanol A (**1**); (**b**) NOE correlations of turcicanol A (**1**).

Turcicanol (**1**) was isolated as an amorphous white powder. The (+)-HRESIMS of **1** showed a [M + H]^+^ molecular ion peak at *m*/*z* 383.2219, suggesting a molecular formula of C_24_H_30_O_4_ for **1** with ten degrees of unsaturation. The ^1^H-NMR spectrum of **1** was closely similar to that of conferol (**8**) (Supplementary Materials Figure S67); thus, this compound should be an unsaturated bi-cyclic drimane sesquiterpene ether of umbelliferone. The most significant difference between the ^1^H-NMR spectra of conferol (**8**) and turcicanol A (**1**) was the lack of ABX signals of the H-11′a and H-11′b protons located at δ_H_ 4.02 and 4.17 ppm (each 1H, dd). The ^1^H-NMR spectrum of **1** displayed two AB-type doublets at δ_H_ 4.40 and 4.55 ppm (each 1H) (see Table 2); such difference strongly suggests that the double bond of **1** was located between C-8′ and C-9′, and the H–9′ proton of conferol (**8**) was not present. The ^13^C-NMR, 2D-COSY, HSQC, and HMBC spectra (Supplementary Materials, Figures S5–S8 and Figure 2a) confirmed the proposed structure of **1** as turcicanol A (Figure 1). The NOE correlations observed in the 2D-NOESY spectrum of **1** (Supplementary Materials, Figure S9 and Figure 2b) clearly confirmed the relative stereochemistry of turcicanol A as depicted in the formula **1** (Figure 1). Thus, turcicanol A (**1**) is a C-8′–C-9′ double-bond isomer of conferol (**8**).

**Figure 3:**
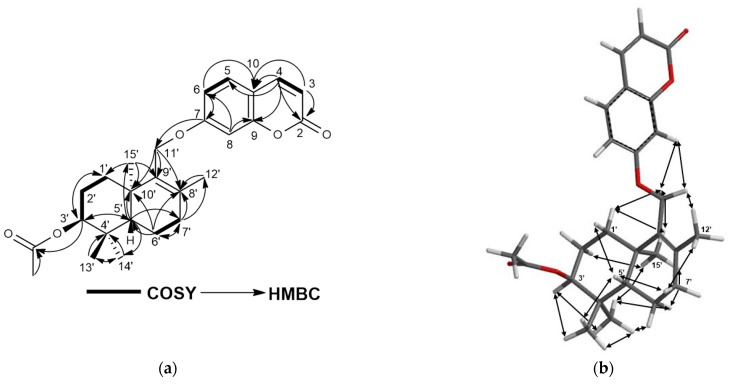
(**a**) COSY and HMBC correlations of turcicanol A acetate (**2**); (**b**) NOE correlations of turcicanol A acetate (**2**).

Turcicanol A acetate (**2**) was isolated as an amorphous white powder. The [M + H]^+^ molecular ion peak observed at *m/z* 425.2325 indicated a C_26_H_32_O_5_ molecular formula for **2** with 11 degrees of unsaturation. The ^1^H-NMR spectrum of **2** was similar to that of turcicanol A (**1**) with the exception of the ca. 1.5 ppm downfield shift of the H-3′ signal to δ 4.69 ppm, and the presence of a methyl singlet at δ_H_ 2.07 ppm clearly suggested the presence of an acetoxy group in **2**. The HMBC correlation from H-3′ (δ_H_ 4.96) to the ester carbonyl at δ_C_ 171.1 established the acetyl group at position 3′ of **2**. The ^13^C-NMR, 2D-COSY, HSQC, and HMBC spectra (Supplementary Materials, Figures S14–S17 and Figure 3a and Table 2) confirmed the proposed structure of **2** as turcicanol A acetate. In addition, the NOE correlations observed in the 2D-NOESY spectrum of **2** (Supplementary Materials, Figure S18 and Figure 3b) clearly confirmed the relative stereochemistry of turcicanol A acetate as depicted in Figure 1.

**Figure 4:**
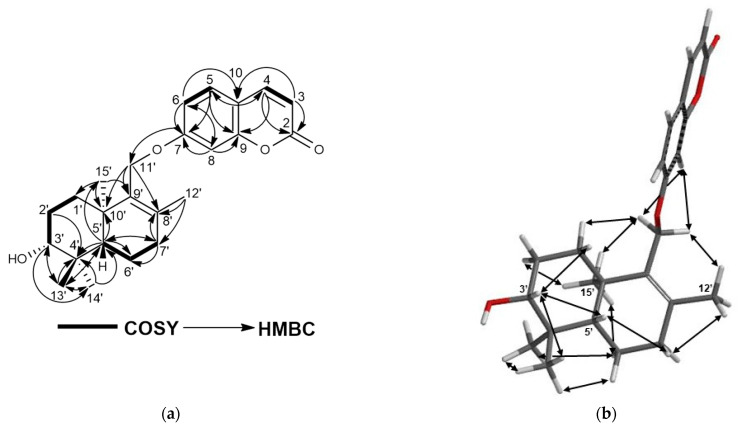
(**a**) COSY and HMBC correlations of turcicanol B (**3**); (**b**) NOE correlations of turcicanol B (**3**).

Turcicanol B (**3**) was isolated as an amorphous white powder. The (+)-HRESIMS of turcicanol B (**3**) showed an [M + H]^+^ molecular ion peak at *m*/*z* 383.2225, which indicated a C_24_H_30_O_4_ molecular formula for **3** with ten degrees of unsaturation. The ^1^H-NMR spectrum of **3** was similar to that of turcicanol A (**1**). The only difference was the shift of the H–3′ proton signal to δ_H_ 3.29 ppm (see Table 2) from δ_H_ 3.49 ppm. The ^1^H-NMR spectrum of turcicanol B (**3**) showed a hydroxyl geminal H-3′ proton as a dd (*J* = 4.5, 11.7 Hz), suggesting an axial orientation; thus, 3′-OH of turcicanol B (**3**) should be equatorial (i.e., α-OH) (Table 2). The ^13^C-NMR, 2D-COSY, HSQC, and HMBC spectra (Supplementary Materials, Figures S23–S26 and Figure 4a and Table 2) indicated the proposed structure for **3** as turcicanol B. The key NOE correlations observed in the 2D-NOESY spectrum of **3** (Supplementary Materials, Figure S27 and Figure 4b) confirmed that the relative configuration of turcicanol B was as depicted in the formula **3** (Figure 1).

**Figure 5:**
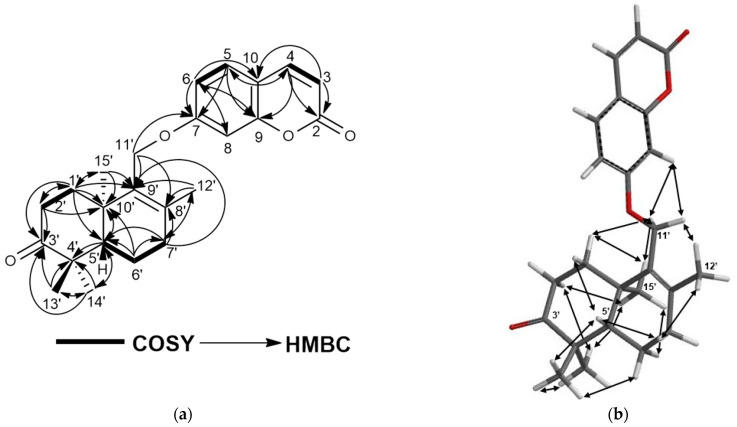
(**a**) COSY and HMBC correlations of turcica ketone (**4**); (**b**) NOE correlations of turcica ketone (**4**).

Compound **4** was isolated as an amorphous white powder. The [M + H]^+^ molecular ion of **4** was observed at *m*/*z* 381.2064 in the (+)-HRESIMS spectrum, indicating a C_24_H_28_O_4_ molecular formula for **4** with 11 degrees of unsaturation. The ^1^H-NMR and ^13^C-NMR spectra of **4** were similar to those of turcicanol A (**1**) and B (**3**) except for the lack of an H-3′ hydroxy geminal proton signal and the presence of a quaternary carbonyl signal at δ_C_ 217 ppm in the ^13^C-NMR spectrum of **4**. The carbonyl signal in the ^13^C-NMR spectrum of **4** clearly showed correlation with the H–1′, H–2′, H–13′, and H–14′ protons in the 2D-HMBC spectrum of **4**, confirming the presence of the carbonyl group at the C-3′ position. Thus, the structure of **4** is the keto form of turcicanol A (**1**) and turcicanol B (**3**) (Table 2). The ^13^C-NMR, 2D-COSY, HSQC, and HMBC spectra (Supplementary Materials, Figures S32–S35 and Figure 5a and Table 2) further corroborated the proposed structure of **4** as turcica ketone. Also, the strong anisotropic shift of the de-shielded H-2′ protons (0.6–0.8 ppm) strongly suggested the presence of a keto group at the C-3′ position (Table 2). Furthermore, the NOE correlations observed in the 2D-NOESY spectrum of **4** (Supplementary Materials, Figure S36 and Figure 5b) clearly confirmed the relative configuration of turcica ketone as shown in formula **4** (Figure 1).

**Figure 6:**
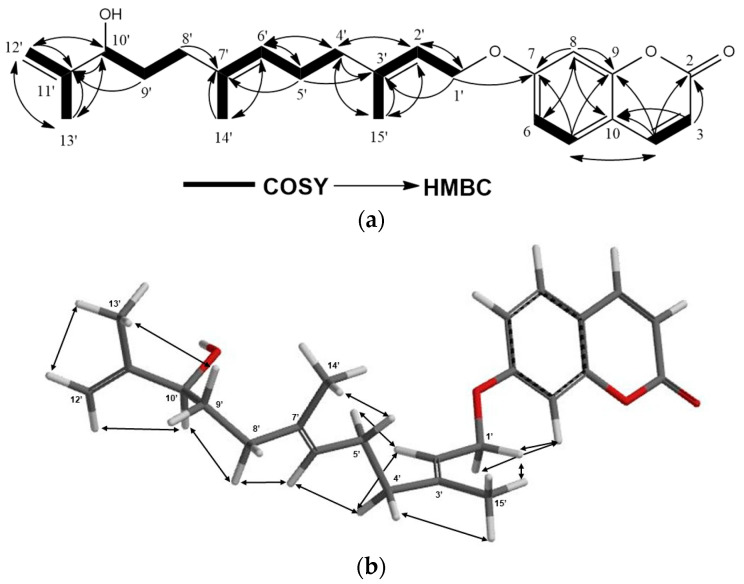
(**a**) COSY and HMBC correlations of 11′-dehydrokaratavicinol (**5**); (**b**) NOE correlations of 11′-dehydrokaratavicinol (**5**).

Compound **5** was isolated as an amorphous white powder. The (+)-HRESIMS spectrum of **5** displayed a [M + Na]^+^ molecular ion peak at *m*/*z* 405.2037, indicating a C_24_H_30_O_4_ molecular formula for **5** with 10 degrees of unsaturation. The ^1^H-NMR spectra of karatavicinol (**32**) (Supplementary Materials Figure S91) and compound **5** are similar except for the presence of two methylene proton singlets at δ_H_ 4.83 and δ_H_ 4.93 ppm in the ^1^H NMR of **5** (brs 1H for each) and the lack of hydroxyl adjacent to the C12′ and C13′ methyl signals of karatavicinol in **5** suggested that a double bond between C-11′ and C-12′ was present in **5**. Furthermore, due to the allylic positioning of the C-10′ hydroxyl group, the chemical shift of the oxygenated methine proton at C-10′ in the ^1^H-NMR spectrum of **5** was shifted downfield ca. 0.7 ppm to δ 4.05 ppm (Table 2). The ^13^C-NMR, 2D-COSY, HSQC, and HMBC spectra (Supplementary Materials, Figures S41–S44 and Figure 6a and Table 2) confirmed the proposed structure of **5** as 11′-dehydrokaratavicinol. The key NOE correlations observed in the 2D-NOESY spectrum of **5** (Supplementary Materials, Figure S45 and Figure 6b) clearly confirmed the geometries of the double bonds of 11′-dehydrokaratavicinol as shown in formula **5** (Figure 1). 

**Figure 7:**
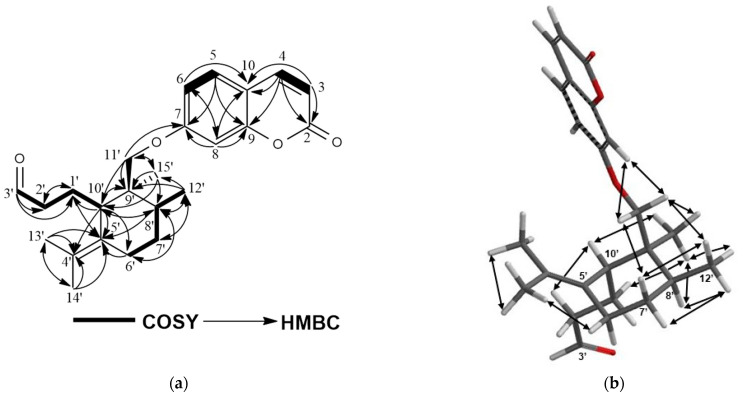
(**a**) COSY and HMBC correlations of galbanaldehyde (**6**); (**b**) NOE correlations of galbanaldehyde (**6**).

Compound **6** was isolated as an amorphous white powder. The [M + H]^+^ molecular ion of compound **6** at *m*/*z* 383.2233 indicated a molecular formula of C_24_H_30_O_4_ for **6** with 10 degrees of unsaturation. The ^1^H-NMR spectrum of **6** was very similar to that of galbanic acid (**27**) (Supplementary Materials, Figure S86) with the exception of the H-3′ signal appearing at δ 9.74 ppm as a narrow triplet, suggesting the presence of an aldehyde group at the C-3′ position (Table 2). The ^13^C-NMR, 2D-COSY, HSQC, and HMBC spectra (Supplementary Materials, Figures S50–S53 and Figure 7a and Table 2) confirmed the proposed structure of compound **6** as galbanaldehyde. The NOE correlations observed in the 2D-NOESY spectrum of **6** (Supplementary Materials, Figure S54 and Figure 7b) confirmed the relative configuration of galbanaldehyde as depicted in the formula **6** (Figure 1), which is identical to that of galbanic acid (**27**) [23,24,25]. 

**Figure 8:**
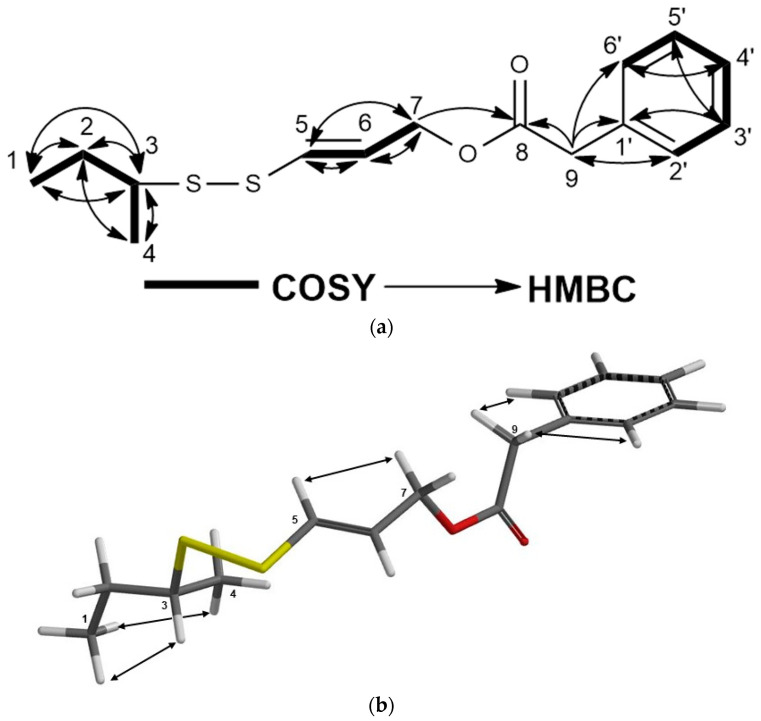
(**a**) COSY and HMBC correlations of turcicasulphide (**7**); (**b**) NOE correlations of turcicasulphide (**7**).

Compound **7** was isolated as colorless oil. The (+)-HRESIMS spectrum of compound **7** exhibited an [M + Na]^+^ molecular ion at *m*/*z* 319.0791, suggesting C_15_H_20_O_2_S_2_ as a molecular formula for turcicasulphide (**7**) with 6 degrees of unsaturation. The ^1^H-NMR spectra of persicasulphide C (**39**) (Supplementary Materials, Figure S98) and compound **7** were very similar to each other with the exception of presence of two doublets and one triplet in the aromatic region of ^1^HNMR, corresponding to the mono-substitute benzene ring in 7. The presence of aromatic and benzylic proton signals at δ_H_ 7.32, 7.29, 7.28, and 3.62 ppm suggested that a benzyl group is present in the molecule. Also, δ_H_ 4.6 (H-7) and δ_C_ 64.4 (C-7) indicated that C-7 is oxygenated. The HMBC correlations from δ_H_ 4.6 (H-7) and δ_H_ 3.62 (H-9) to δ_C_ 171.3 (C-8) connected the benzyl group to the position 7 of **7** through an ester linkage. This indicated that the 3-hydroxyisovalerate ester of persicasulphide C (**39**) was replaced with a benzyl ester in turcicasulphide (**7**) (Table 2). The ^13^C-NMR, 2D-COSY, HSQC, and HMBC spectra (Supplementary Materials, Figures S59–S62 and Figure 8a and Table 2) confirmed the proposed structure for **7** as turcicasulphide. The *J* coupling constant of 14.8 Hz between H-5 and H-6 indicated that these protons are trans to each other. The NOE correlations observed in the 2D-NOESY spectrum of **7** (Supplementary Materials, Figure S63 and Figure 8b) clearly confirmed the relative configuration of position 3 as shown in formula **7** (Figure 1). 

**Figure 9:**
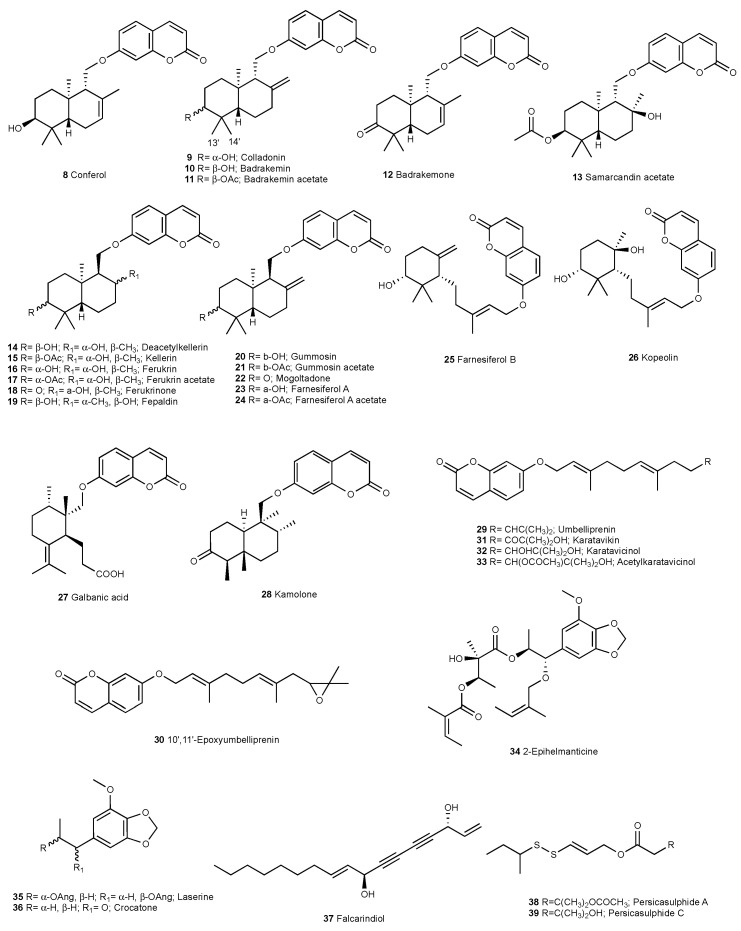
Known compounds isolated from *Ferula turcica* roots.

The known compounds conferol (**8**) [26], colladonin (**9**) [27], badrakemin (**10**) [27], badrakemin acetate (**11**) [28], badrakemone (**12**) [26], samarcandin acetate (**13**) [26], deacetylkellerin (**14**) [26], kellerin (**15**) [26], ferukrin (**16**) [26], ferukrin acetate (**17**) [26], ferukrinone (**18**) [29], fepaldin (**19**) [30,31], gummosin (**20**) [26], gummosin acetate (**21**) [29], mogoltadone (**22**) [26], farnesiferol A (**23**) [26], farnesiferol A acetate (**24**) [26], farnesiferol B (**25**) [32], kopeolin (**26**) [33], galbanic acid (**27**) [34], kamolone (**28**) [35], umbelliprenin (**29**) [36], 10′,11′-epoxyumbelliprenin (**30**) [37], karatavikin (**31**) [38], karatavicinol (**32**) [39], 10′-acetylkaratavicinol (**33**) [40], 2-epihelmanticine (**34**) [41], laserine (**35**) [42], crocatone (**36**) [43], falcarindiol (**37**) [44], persicasulphide A (**38**) [45], and persicasulphide C (**39**) [45] (Figure 9) were identified by the comparison of their spectroscopic data with that of the literature data. 

Most of the sesquiterpene coumarins isolated from the roots of *Ferula turcica* were bicyclic drimane sesquiterpene ethers of umbelliferone. The C-11′ hydroxymethylene group of the drimane sesquiterpene forms an ether linkage between the 7-OH of umbelliferone and the drimane moiety. The orientation of the C-11′ hydroxymethylene group of the drimane moiety was determined as equatorial in compounds **8**–**13** and axial in compounds **14**–**24** by NOE correlations observed in the 2D-NOESY spectra of those compounds. As it was shown by X-ray crystallography of the *R*-MTPA ester derivative of samarcandin and chemical transformations [26], stereochemistries of the other methyl groups of compounds **8**–**24** were identical, and their absolute configurations should be as depicted in formulas **8**–**24**. Thus, the absolute configurations of the biogenetically related turcicanol derivatives should be as shown in formulas **1**–**4**.

### 2.2. Cytotoxic Activity

The pure compounds of *Ferula turcica* were tested against colon cancer cell lines (COLO 205 and HCT 116) and kidney cell lines (UO31 and A498). The results are given in Table 3.

According to the cytotoxicity studies, the acetylation of the hydroxyl C-10 position in the sulfur-bearing compounds (compounds **38**, **39**) preserved the cytotoxic activity in the UO31 and COLO205 cell lines and slightly increased the activity in HCT116; however, the loss of hydroxyl at the C-10 and substitution of a benzene ring led to the loss of cytotoxic activity (see compound **7**) in all cell lines. The cytotoxic activity was observed in colladonin (**9**) but not in badrakemin (**10**), whose hydroxyl substitution at the C-3′ was in the *β* position. In addition, the cytotoxic activity in gummosin (**20**), which is the C-9′ epimer of badrakemin (**10**), and colladonin (**9**) increased the cytotoxic activity with axial stereochemistry. The acetate derivatives of badrakemin (**10**) and gummosin (**20**) as well as the cytotoxic activity of badrakemin acetate (**11**) increased slightly in HCT116 cell lines, and the activity of gummosin acetate (**21**) decreased in COLO205 cell lines, decreased in HCT116, increased in A498, and decreased in UO31 cell lines. As for the ketone derivatives, the oxidation products at C-3′ of badrakemin (**10**) and gummosin (**20**) as well as oxidation did not cause any increase in badrakemone (**12**); it caused a decrease in cytotoxic activity in mogoltadone (**22**). Conferol (**8**) showed cytotoxic activity in colon cancer cell lines. We noted that even with the double-bond shift to C-8′ and C-9′ positions, cytotoxic activity in turcicanol A (1) is still significant in HCT116 colon cancer cells, just as in its isomers conferol (**8**) and gummosin (**20**). It was determined that the acetylation of the compound (turcicanol A acetate **2**) causes activity to be lost in these four cell lines. In turcicanol B (**3**), an increase in cytotoxic activity was observed in the UO31 kidney cell line. When the cytotoxic activity results of the pure compound turcica ketone (**4**), an isomer of badrakemone (**12**) and mogoltadone (**22**), were examined, the endocyclic double bond was more cytotoxic than the exocyclic double bond, as in badrakemone (**12**) and mogoltadone (**22**). The cytotoxic activity results of galbanic acid (**27**) and its aldehyde derivative (**6**) showed that the aldehyde form of C-3′ increased the cytotoxic activity on HCT116 colon cancer cell lines. 

## 3. Discussion

The literature data show that sesquiterpene coumarins such as gummosin, badrakemin acetate, ferukrinone, deacetylkellerin, farnesiferol A, farnesiferol B, farnesiferol C, samarcandin, umbelliprenin, kellerin, and gummosin have significant cytotoxic effects on breast (MCF-7) and prostate (PC-3) cancer cell lines (cytotoxic activity 30 and 32.1 µg/mL, respectively) [46]. Tosun et al. examined the cytotoxic activities of pure compounds on kidney cancer cells (UO31 and A498), colon cancer cells (COLO205 and KM12), and Ewing sarcoma cancer cell lines (A673 and TC32) and determined that umbelliprenin (1.8 µM), karatavicinol (7.6 µM), badrakemone (11 µM), badrakemin (0.38 µM), and colladonin (0.75 µM) suppressed growth of the UO31 kidney cancer cell line, while badrakemin (9.1 µM) and colladonin (2.5 µM) showed cytotoxic activity in the KM12 colon cancer cell line [27]. In another study, galbanic acid, a sesquiterpene coumarin obtained from *F. szowitsiana* roots, and farnesiferol A isolated from *Ferula persica* roots were found to be effective on doxorubicin-resistant breast cancer (MCF-7/Adr) cell lines [47]. In a combination study with sesquiterpene coumarins and doxorubucin, it was determined that the cytotoxic activity of doxorubicin was increased against MCF-7/Adr resistant cell lines, and the best result was seen with the combination of doxorubucin + lehmferin; it was determined that the activity increases in resistant cells during the using of the combination (5.08 µM) in contrast to doxorubucin usage alone (21.41 µM) [48]. 

According to the IC_50_ values, seventeen compounds (**1**, **3**, **4**, **6**, **15**, **17**, **19**–**23**, **25**, **28**–**30**, **32**, **38**, and **39**) showed cytotoxic activity in this study. The most effective compounds against cancer cell lines were determined as conferol (**8**), gummosin (**20**), and persicasulphide A (**38**) and C (**39**), which is in agreement with the literature data. While it is true that no clear structure–activity patterns emerged from the testing, the diversity of structures may have limited any such conclusions.

## 4. Materials and Methods

### 4.1. General Experimental Procedures

LC-MS analysis was performed with Agilent Technologies^®^ 6130 Quadrupole LC/MS (Santa Clara, CA, USA). UV–vis spectra were obtained using Shimadzu^®^ UV-1700 PharmaSpec (Kyoto, Japan). IR spectra were determined using Bruker^®^ Alpha FT-IR (Billerica, MA, USA). NMR spectra of the compounds were acquired on a Bruker^®^ Avance III spectrometer operating at 600 MHz for ^1^H and 150 MHz for ^13^C in deuterated chloroform (Billerica, MA, USA). HRESIMS analysis of compounds **1**–**6** were performed using Agilent^®^ 6530 Accurate Mass Q-TOF (Santa Clara, CA, USA), while the HRESIMS data of turcicasulphide (**7**) were acquired on a Thermo Scientific-Q Exactive^®^ (Waltham, MA, USA). Optical rotation data were acquired using a Rudolph Analytical Autopol V Plus^®^ in dichloromethane (Hackettstown, NJ, USA). A Buchi rotary evaporator was used to evaporate the solvent of the extract (Buchi, Flawil, Switzerland). A Sephadex LH-20 (Sigma Chem. Co. 25–100 µm) (GE Healthcare, Chicago, IL, USA) column (5 × 100 cm) was used for the initial fractionation. A Gilson^®^ PLC 2050 was used for the further purification of the compounds (Saint-Avé, France). Hexane, dichloromethane, methanol, and acetonitrile (Merck, Darmstadt, Germany) were used during the chromatographic analyses.

### 4.2. Plant Material

The plant root materials used in this study were collected from the shores of Tuz Lake in Konya (Yavşan Tuzlası) on 16 June 2015, while the plant was fruiting, and the voucher specimen was archived in ISTE (Istanbul University Faculty of Pharmacy Herbarium) with the number 116,464. The species was identified by Prof. Emine Akalın and Hüseyin Onur Tuncay [6].

### 4.3. Extraction and Isolation

The powdered roots (270 g) of *Ferula turcica* were extracted by maceration at room temperature with dichloromethane (2 × 1 L) for 1 h in a Soxhlet extractor. After maceration, the plant material was further subjected to continuous extraction with dichloromethane and then with methanol using continuous extraction in a Soxhlet extractor. The dichloromethane extracts obtained by maceration and continuous extraction were concentrated separately under reduced pressure in a rotary evaporator at 35 °C. Since the TLC comparison of the extracts obtained by maceration and continuous extraction with dichloromethane showed close similarity, they were combined to yield the dichloromethane extract, 12 g (yield 4.5%). The methanol extract was evaporated to obtain 11 g (yield 4.1%) [26]. The cytotoxic dichloromethane extract (8.6 g) was fractionated on a Sephadex LH-20 column (5 × 100 cm) using a hexane: dichloromethane: methanol (7:4.5:0.5) solvent system as an initial mobile system, and the mobile system was eluted until 7:1:4 with the same solvent order [26]. The secondary metabolite profile of each fraction was examined by TLC chromatography, and similar fractions were combined to yield 30 fractions (Supplementary Material, Figure S1). Cytotoxic fractions with approximately 200 mg of mass (i.e., FST 10–29) were further purified on a reverse-phase preparative HPLC with a gradient elusion at a flow rate of 9 mL/min for 1 h to obtain 60 fractions. Fractions with less mass (<20 mg) were subjected to reverse-phase semipreparative HPLC purification at a flow rate of 4 mL/min for 45 min to obtain 45 fractions (one-minute collection) or 90 fractions (half-a-minute collection). Chromatograms were observed at 200–600 nm, 210 nm, 254 nm, 280 nm, and 366 nm wavelengths during HPLC purification. A Luna 5 µ Phenomenex^®^ (21.2 × 150 mm) and a Luna 5 µ Phenomenex^®^ (10 × 250 mm) C18 columns were used for purification. Acetonitrile and water were used as the mobile phase. The mobile phase composition was modified according to the polarity of fractions [49]. Thirty-two known compounds, namely samarcandin acetate (**13**, 10 mg), deacetylkellerin (**14**, 25.9 mg), kellerin (**15**, 61.8 mg), ferukrin (**16**, 29.3 mg), ferukrin acetate (**17**, 3.8 mg), ferukrinone (**18**, 5.3 mg), fepaldin (**19**, 0.6 mg), colladonin (**9**, 1 mg), badrakemin (**10**, 1.6 mg), badrakemin acetate (**11**, 6.2 mg), badrakemone (**12**, 1.3 mg), conferol (**8**, 1.3 mg), gummosin (**20**, 42.8 mg), gummosin acetate (**21**, 30.6 mg), mogoltadone (**22**, 77 mg), farnesiferol A (**23**, 24.1 mg), farnesiferol A acetate (**24**, 1.8 mg), farnesiferol B (**25**, 1.7 mg), kopeolin (**26**, 0.7 mg), galbanic acid (**27**, 5.6 mg), kamolone (**28**, 1.1 mg), umbelliprenin (**29,** 29.1 mg), 10′,11′-epoxyumbelliprenin (**30**, 0.8 mg), karatavikin (**31**, 1 mg), karatavicinol (**32**, 0.3 mg), 10′-acetylkaratavicinol (**33**, 6.3 mg), 2-epihelmanticine (**34**, 8 mg), laserine (**35**, 12 mg), crocatone (**36**, 0.8 mg), falcarindiol (**37**, 1.5 mg), persicasulphide A (**38,** 64.6 mg), persicasulphide C (**39**, 10 mg), and seven new compounds, turcicanol A (**1**, 2.9 mg), turcicanol A acetate (**2**, 4.3 mg), turcicanol B (**3**, 0.4 mg), turcica ketone (**4**, 1.4 mg), 11′-dehydrokaratavicinol (**5**, 3 mg), galbanaldehyde (**6**, 4.7 mg), and turcicasulphide (**7**, 0.6 mg), were obtained from the dichloromethane extract of *F. turcica*. (See Supplementary Materials Figure S1 for the isolation chart.) 

Turcicanol A (**1**, 2.9 mg): Amorphous white powder, [α${]}_{D}^{24}$: −39° (c, 0.076 mg/mL, CH_2_Cl_2_); UV (c, 0.012 mg/mL) (MeOH) λ_max_ (log ε) nm: 203 (4.59), 218 (sh) (4.12), 295 (sh) (3.83) nm, 324 (4.10) nm. IR υ_max_ (NaCl) cm^−1^: 3053, 2942, 2866, 2826, 1730, 1613, 1554, 1507, 1475, 1455, 1427, 1402, 1385, 1349, 1278, 1230, 1198, 1156, 1124, 1096, 1062, 995, 921, 892, 835, 758, 736, 702, 662, 634, 616, 595, 544, 518, 492, 472, 459, 435, 419 cm^−1^. ^1^H-NMR and ^13^C-NMR data are in Table 2; HRESIMS *m*/*z* [M + H]^+^ 383.2219 (calculated for C_24_H_31_O_4_: 383.2222).

Turcicanol A acetate (**2**, 4.3 mg): Amorphous white powder, [α${]}_{D}^{24}$: −31° (c, 0.089 mg/mL, CH_2_Cl_2_); UV (c, 0.015 mg/mL) (MeOH) λ_max_ (log ε) nm: 204 (4.71) nm, 219 (sh) (4.23) nm, 295 (sh) (3.96), 324 (4.23) nm. IR υ_max_ (NaCl) cm^−1^: 3078, 3055, 2943, 2875, 2831, 1732, 1612, 1555, 1507, 1455, 1428, 1402, 1375, 1350, 1276, 1248, 1198, 1183, 1157, 1123, 1096, 1047, 1015, 996, 971, 891, 836, 736, 702, 664, 634, 615, 543, 516, 480, 460, 417, 407 cm^−1^. ^1^H-NMR and ^13^C-NMR data are in Table 2; HRESIMS *m*/*z* [M + H]^+^ 425.2325 (calculated for C_26_H_33_O_5_: 425.2328).

Turcicanol B (**3**, 0.4 mg): Amorphous white powder, [α${]}_{D}^{24}$: −43° (c, 0.04 mg/mL, CH_2_Cl_2_); UV (c, 0.012 mg/mL) (MeOH) λ_max_ (log ε) nm: 203 (4.46) nm, 220 (sh) (3.94), 296 (sh) (3.62), 324 (3.86) nm. IR υ_max_ (NaCl) cm^−1^: 2956, 2922, 2869, 2847, 1733, 1611, 1554, 1507, 1460, 1402, 1377, 1350, 1277, 1299, 1197, 1156, 1123, 1097, 1036, 997, 892, 835, 735, 700, 615, 461, 444, 415 cm^−1^. ^1^H-NMR and ^13^C-NMR data are in Table 2; HRESIMS *m/z* [M + H]^+^ 383.2225 (calculated for C_24_H_31_O_4_: 383.2222).

Turcica ketone (**4**, 1.4 mg): Amorphous white powder, [α${]}_{D}^{24}$: −14° (c, 0.04 mg/mL, CH_2_Cl_2_); UV (c, 0.012 mg/mL) (MeOH) λ_max_ (log ε) nm: 203 (4.50) nm, 217 (sh) (4.06), 295 (sh) (3.75) nm, 324 (4.00) nm. IR υ_max_ (NaCl) cm^−1^: 3077, 3057, 2954, 2929, 2871, 1731, 1704, 1611, 1554, 1507, 1459, 1428, 1401, 1382, 1348, 1276, 1229, 1196, 1156, 1122, 996, 974, 891, 834, 745, 702, 634, 615, 593, 534, 494, 477, 456, 419 cm^−1^. ^1^H-NMR and ^13^C-NMR data are in Table 2; HRESIMS *m/z* [M + H]^+^ 381.2064 (calculated for C_24_H_29_O_4_: 381.2066).

11′-Dehydrokaratavicinol (**5**, 3 mg): Amorphous white powder, [α${]}_{D}^{24}$: −1.5° (c, 0.003 mg/mL, CH_2_Cl_2_); UV (c, 0.012 mg/mL) (MeOH) λ_max_ (log ε) nm: 203 (4.58) nm, 215 (sh) (4.12), 299 (sh) (3.78), 324 (4.02) nm. IR υ_max_ (NaCl) cm^−1^: 3072, 2933, 2856, 1732, 1613, 1555, 1507, 1446, 1402, 1350, 1278, 1231, 1199, 1157, 1127, 1096, 1060, 1000, 895, 835, 757, 683, 634, 616, 559, 516, 460, 418, 406 cm^−1^. ^1^H-NMR and ^13^C-NMR data are given in Table 2; HRESIMS *m*/*z* [M + Na]^+^ 405.2037 (calculated for C_24_H_30_O_4_Na: 405.2042).

Galbanaldehyde (**6**, 4.7 mg): Amorphous white powder, [α${]}_{D}^{24}$: −24° (c, 0.078 mg/mL, CH_2_Cl_2_); UV (c, 0.012 mg/mL) (MeOH) λ_max_ (log ε) nm: 203 (4.80), 218 (sh) (4.29) nm, 295 (sh) (4.02), 324 (4.30) nm. IR υ_max_ (NaCl) cm^−1^: 3081, 3053, 2962, 2923, 2879, 2656, 1719, 1731, 1729, 1613, 1555, 1509, 1454, 1428, 1302, 1351, 1279, 1231, 1199, 1156, 1122, 1096, 1011, 988, 891, 835, 751, 735, 703, 633, 616, 546, 533, 514, 459, 417 cm^−1^. ^1^H-NMR and ^13^C-NMR data are given in Table 2; HRESIMS *m*/*z* [M + H]^+^ 383.2233 (calculated for C_24_H_31_O_4_: 383.2222).

Turcicasulphide (**7**, 0.6 mg): Colorless oil, UV (c, 0.013 mg/mL) (MeOH) λ_max_ (log ε) nm: 203 (4.52) nm, 234 (sh) (4.09), 322 (3.58) nm. IR υ_max_ (NaCl) cm^−1^: 2962, 2925, 2870, 2857, 1736, 1611, 1494, 1451, 1373, 1338, 1276, 1241, 1144, 993, 972, 939, 835, 722, 702, 464 cm^−1^. ^1^H-NMR and ^13^C-NMR data are in Table 2; HRESIMS *m*/*z* [M + Na]^+^ 319.0791 (calculated for C_15_H_20_O_2_S_2_Na: 319.0802).

### 4.4. 2DAY (Colon 2) XTT Cytotoxic Activity Assay

The two-XTT bioactivity test is an in vitro colorimetric cytotoxic activity test developed by the NCI MTP Assay Development and Screening Section [50] and used for this study. Colon (COLO205, HCT116) and kidney (A498, UO31) cancer cell lines were used during the tests. RPMI-1640 (Roswell Park Memorial Institute, Buffalo, NY, USA) medium, 10% FBS (fetal bovine serum), 1% glutamine, and 1% penicillin/streptomycin solutions were used for cell growth and treatment. Transfers were performed under laminar air flow in a sterile environment. The suspension containing the cells was seeded into 96-well plates with a volume of 45 µL with 3.5 × 10^5^ cells per well. Then, the plate was incubated at 37 °C and 5% CO_2_ for 24 h. The extract and pure compounds prepared in DMSO were added and incubated for another 48 h. After incubation, 10 µL of the tetrazolium salt XTT (2,3-bis[2-methoxy-4-nitro-5-sulfophenyl]-2*H*-tetrazolium-5-carboxanolide) was applied to the cells. After 4 h of incubation, dead cells were not stained with formazan dye, while viable cells could be counted in the EnVision plate reader under UV light (450 nm and 650 nm). Sanguinarine chloride hydrate was used as a positive control in the experiment.

## 5. Conclusions

A dichloromethane extract of *Ferula turcica* root was studied for the first time. Seven new and thirty-two known compounds (**1**–**39**) were isolated from the dichloromethane extract using bioactivity-directed fractionation, and their cytotoxic activities were investigated against COLO205, HCT116, A498, and UO31 cancer cell lines. The structures of the new compounds were determined by spectroscopic techniques, and the spectral data of the compounds are presented for the first time. Some structure–activity relationships of the compounds for cytotoxic activities illuminate the effects of substitution, oxidation, acetylation, and double bond positions.

## Figures and Tables

**Table 1:** Cytotoxic activities of *Ferula turcica* root extracts.

Extracts	IC_50_ (µg/mL)			
	COLO 205	HCT116	A498	UO31
Dichloromethane extract	6.8	16.8	20	13.9
Methanol extract	>100	>100	>100	>100

**Table 2:** ^1^H NMR and ^13^C NMR shifts of compounds **1**–**4** (in CDCl_3_, δ in ppm, and *J* in Hz).

Position	Turcicanol A (1)		Turcicanol A Acetate (2)		Turcicanol B (3)		Turcica Ketone (4)	
	^1^H-NMR	^13^C-NMR	^1^H-NMR	^13^C-NMR	^1^H-NMR	^13^C-NMR	^1^H-NMR	^13^C-NMR
**2**	-	161.5	-	161.5	-	161.4	-	161.4
**3**	6.25; d; *9.4;* 1H	113.0	6.24; d; *9.5*; 1H	113.1	6.25; d; *9.4*; 1H	113.1	6.25; d; *9.4*; 1H	113.2
**4**	7.64; d; *9.4*; 1H	143.5	7.64; d; *9.5*; 1H	143.6	7.64; d; *9.4*; 1H	143.6	7.64; d; *9.4*; 1H	143.5
**5**	7.36; d; *8.9*; 1H	128.8	7.37; d; *8.5*; 1H	128.8	7.37; d; *7.5*; 1H	128.7	7.37; d; *9.2*; 1H	129.0
**6**	6.87; dd; *2.3; 8.9*; 1H	113.4	6.89; dd; *2.2; 8.5*; 1H	113.2	6.87; dd; *2.3; 7.5*; 1H	113.3	6.85; dd; *2.4; 9.2*; 1H	113.2
**7**	-	162.9	-	162.5	-	162.5	-	162.3
**8**	6.87; d; *2.3*; 1H	101.4	6.87; d; *2.2*; 1H	101.6	6.86; d; *2.3*; 1H	101.5	6.86; d; *2.4*; 1H	101.5
**9**	-	156.2	-	156.0	-	156.0	-	156.0
**10**	-	112.5	-	112.5	-	112.6	-	112.7
**1′α**	1.80; td; *3.7; 13.4*; 1H	29.4	1.45; dd; *3.1; 9.6*; 1H	30.0	1.70; m; 1H **	34.5	1.91; dd; *6.3; 8.5*; 2H	34.7
**1′β**	1.43; dt; *3.7; 13.4*; 1H		1.67; m; 1H *		1.49; td; *2.4; 13.1*; 1H			
**2′α**	1.96; tt; *3.3; 14.3*; 1H	25.7	1.90; tddd; *1.7; 5.3; 14.1*; 1H	23.3	1.70; m; 2H **	27.8	2.51; m; 2H *	34.2
**2′β**	1.62; dq; *3.3; 15.3*; 1H		1.64; m; 1H *					
**3′**	3.46; t; *2.8*; 1H	75.8	4.69; t; *2.4*; 1H	77.7	3.29; dd; *4.5; 11.7*; 1H	78.9	-	217.0
**4′**	-	37.7	-	36.9	-	38.9	-	47.2
**5′**	1.68; m; 1H *	44.8	1.7; dd; *1.7; 10.7*; 1H	45.9	1.24; dd; *2; 12.3*; 1H	50.7	1.83; dd; *2.4; 12.4*; 1H	50.8
**6′α**	1.54; qd; *6.5; 12.6*; 1H	18.5	1.53; m; 1H *	18.4	1.54; m; 1H *	18.7	1.61; ddd; *6.5; 11.1; 12.7*; 1H	19.9
**6′β**	1.64; m; 1H *		1.66; m; 1H *		1.75; dd; *6.8; 13.5*; 1H		1.67; ddt; *2.4; 6.5; 8.6*; 1H	
**7′α**	2.16; dtd; *6.8; 18.2*; 2H	33.8	2.16; td; *6.4; 18.5*; 1H	33.6	2.17; d; *6.8*; 2H	34.0	2.21; m; 2H *	33.6
**7′β**			2.20; d; *7.6*; 1H					
**8′**	-	135.8	-	136.0	-	136.2	-	136.7
**9′**	-	135.3	2.93; t; *5.1*; 1H	135.2	-	135.1	-	133.7
**10′**	-	37.8	-	37.8	-	37.9	-	37.67
**11′a**	4.40; d; *9.9*; 1H	64.6	4.56; d; *9.9*; 1H	64.7	4.38; d; *9.9*; 1H	64.8	4.41; d; *10.1*; 1H	64.7
**11′b**	4.55; d; *9.9*; 1H		4.40; d; *9.9*; 1H		4.54; d; *9.9*; 1H		4.55; d; *10.1*; 1H	
**12′**	1.68; s; 3H	19.6	1.71; s; 3H	19.7	1.69; s; 3H	19.5	1.72; s; 3H	19.7
**13′**	0.88; s; 3H	22.1	0.90; s; 3H	27.7	0.83; s; 3H	15.7	1.08; s; 3H	21.1
**14′**	1.00; s; 3H	28.2	0.94; s; 3H	21.7	1.04; s; 3H	28.2	1.13; s; 3H	27.0
**15′**	1.04; s; 3H	20.8	1.05; s; 3H	20.7	1.03; s; 3H	20.9	1.11; s; 3H	20.6
**CH3–(OAc)**	-	-	2.07; s; 3H	21.5	-	-	-	-
**C=O (OAc)**	-	-	-	171.1	-	-	-	-
**Position**	**11′-Dehydrokaratavicinol (5)**		**Galbanaldehyde (6)**				**Turcicasulphide (7)**	
	**^1^** **H-NMR**	**^13^** **C-NMR**	**^1^** **H-NMR**		**^13^** **C-NMR**		**^1^** **H-NMR**	**^13^** **C-NMR**
**1**	-	-	-		-		0.97; t; *7.4*; 3H	11.7
**2a**	-	161.5	-		161.2		1.51; m; 1H *	28.2
**2b**							1.68; td; *6.5; 13.2*; 1H	
**3**	6.25; d; *9.5*; 1H	113.0	6.24; d; *9.4*; 1H		113.0		2.79; h; *6.7*; 1H	48.1
**4**	7.64; d; *9.5*; 1H	143.7	7.63; d; *9.4*; 1H		143.2		1.28; d; *6.9*; 3H	20.2
**5**	7.35; d; *8.6*; 1H	128.8	7.34; d; *8.6*; 1H		128.7		6.31; dt; *1.3; 14.8*; 1H	133.1
**6**	6.85; dd; *2.4; 8.6*; 1H	113.4	6.81; dd; *2.4; 8.6*; 1H		113.3		5.96; dt; *6.5; 14.8*; 1H	122.5
**7**	-	162.2	-		162.8		4.6; dd; *1.3; 6.5;* 2H	64.4
**8**	6.82; d; *2.4*	101.6	6.75; d; *2.4*; 1H		101.2		-	171.3
**9**	-	155.8	-		156.0		3.63; s; 2H	41.5
**10**	-	112.5	-		112.4		-	-
**1′**	4.60; d; *6.5*; 2H	65.4	1.84; m; 2H**		19.6		-	134.0
**2′**	5.46; td; *1.2; 6.5*; 1H	118.6	2.29; t; *7.6*; 2H		42.3		7.29; m; 1H *	129.4
**3′**	-	142.2	9.74; t; *1.5*; 1H		203.5		7.32; m; 1H **	128.3
**4′**	2.1; dd; *4.6; 11.6*; 2H	39.6	-		126.5		7.28; m; 1H *	127.3
**5′**	2.15; q; *6.7*; 2H	26.2	-		129.7		7.32; m; 1H **	128.7
**6′α**	5.14; t; *6.7*; 1H	124.1	1.86; m; 1H **		24.6		7.29; m; 1H *	129.4
**6′β**			2.5; dt; *3.1; 14.3*; 1H					
**7′α**	-	135.4	1.2; dd; *4.6; 13.5*; 1H		32.0		-	-
**7′β**			1.6; m; 1H *					
**8′a**	1.99; ddd; *6.4; 9.1; 14.4*; 1H	35.8	1.9; m; 1H **		34.8		-	-
**8′b**	2.03; ddd; *6.22; 9.1; 15.1*; 1H						-	-
**9′**	1.63; m; 2H *	33.1	-		40.8		-	-
**10′**	4.03; dd; *5.4; 7.5*; 1H	75.6	2.92; dd; *4.2; 12*; 1H		42.7		-	-
**11′a**	-	147.7	3.7; d; *8.2*; 1H		71.8		-	-
**11′b**			3.87; d; *8.2*; 1H					
**12′a**	4.83; brs; 1H	111.2	0.91; d; *6.7*; 3H		16.1		-	-
**12′b**	4.93; brs; 1H							
**13′**	1.72; s; 3H	17.8	1.43; s; 3H		20.4		-	-
**14′**	1.61; s; 3H	16.1	1.61; s; 3H		20.4		-	-
**15′**	1.75; s; 3H	16.9	1.16; s; 3H		22.6			

*, ** Partially overlapped signals.

**Table 3:** IC_50_ values of *Ferula turcica* compounds.

Compound		IC_50_ (µM)			
		Colo205	HCT116	A498	UO31
**1**	Turcicanol A	>50	**41.1**	>50	>50
**2**	Turcicanol A acetate	>50	>50	>50	>50
**3**	Turcicanol B	>50	>50	>50	**38.3**
**4**	Turcica ketone	**37.3**	**37.1**	>50	**32.2**
**5**	11′-Dehydrokaratavicinol	>50	>50	>50	>50
**6**	Galbanaldehyde	>50	**48.8**	>50	>50
**7**	Turcicasulphide	>50	>50	>50	>50
**8**	Conferol	**16.9**	**36.2**	>50	>50
**9**	Colladonin	**35.9**	**47.4**	>50	**33.1**
**10**	Badrakemin	>50	>50	>50	>50
**11**	Badrakemin acetate	>50	**46.1**	>50	**44.9**
**12**	Badrakemone	>50	>50	>50	>50
**13**	Samarcandin acetate	>50	>50	>50	>50
**14**	Deacetylkellerin	>50	>50	>50	>50
**15**	Kellerin	>50	>50	>50	>50
**16**	Ferukrin	>50	>50	>50	>50
**17**	Ferukrin acetate	>50	>50	>50	>50
**18**	Ferukrinone	>50	>50	>50	>50
**19**	Fepaldin	>50	>50	>50	>50
**20**	Gummosin	**12.7**	**18**	>50	**19.7**
**21**	Gummosin acetate	>50	**32.2**	**43.3**	**31.2**
**22**	Mogoltadone	**46.9**	>50	>50	>50
**23**	Farnesiferol A	**35.7**	>50	>50	**45.3**
**24**	Farnesiferol A acetate	>50	>50	>50	>50
**25**	Farnesiferol B	**42.3**	>50	>50	>50
**26**	Kopeolin	>50	>50	>50	>50
**27**	Galbanic acid	>50	>50	>50	>50
**28**	Kamolone	>50	**43.5**	>50	>50
**29**	Umbelliprenin	**49.5**	>50	>50	>50
**30**	10′,11′-Epoxyumbelliprenin	**44.4**	>50	>50	>50
**31**	Karatavikin	>50	>50	>50	>50
**32**	Karatavicinol	>50	>50	>50	**34.4**
**33**	10′-Acetylkaratavicinol	>50	>50	>50	>50
**34**	2-Epihelmanticine	>50	>50	>50	>50
**35**	Laserine	>50	>50	>50	>50
**36**	Crocatone	>50	>50	>50	>50
**37**	Falcarindiol	>50	>50	>50	>50
**38**	Persicasulphide A	**49.9**	**15.8**	>50	**42.5**
**39**	Persicasulphide C	**19.6**	>50	>50	**25.1**

## Data Availability

Not applicable.

## Supplementary Materials

The following supporting information can be downloaded at https://www.mdpi.com/article/10.3390/molecules28155733/s1, Figures S1–S108: LC-MS analysis of the dichloromethane extract of *F. turcica*, isolation chart of the dichloromethane extract of *F. turcica*, structures of pure compounds, the ^1^D and ^2^D NMR, HRESIMS, UV, and IR data of pure compounds **1**–**7** as well as the ^1^H-NMR spectra of compounds **8**–**39** and cytotoxic activity graphic of pure compounds **1**–**39**.
