# Supplementary material for: Novel Cytotoxic Sesquiterpene Coumarin Ethers and Sulfur-Containing Compounds from the Roots of Ferula turcica
Source: Molecules. 2023 Jul 28;28(15):5733. doi: 10.3390/molecules28155733 (PMC10420993; doi:10.3390/molecules28155733)
Supplement: Supplementary file 1 [file molecules-28-05733-s001.zip › molecules-2519715-supplementary.pdf]

SUPPLEMENTARY MATERIALS

Novel cytotoxic sesquiterpene coumarin ethers and sulfur-containing compounds from the roots of *Ferula turcica*

Fatma Memnune Eruçar [1,2,3], Sarath P. D. Senadeera [3], Jennifer A. Wilson [3], Ekaterina Goncharova [3,4], John A. Beutler [3,*] and Mahmut Miski [1,*]

[1] Department of Pharmacognosy, Faculty of Pharmacy, Istanbul University, Istanbul 34116, Türkiye; memnune.erucar@istanbul.edu.tr

[2] Department of Pharmacognosy, Institute of Graduate Studies in Health Sciences, Istanbul University, Istanbul 34116, Türkiye (F.M.E.)

[3] Molecular Targets Program, National Institutes of Health, National Cancer Institute, Frederick, MD 21702, USA; dayani.sarathparakumge@nih.gov (S.P.D.S.); wilsonje@mail.nih.gov (J.A.W.); katya.goncharova@nih.gov (E.G.)

[4] Advanced Biomedical Computational Science, Frederick National Laboratory for Cancer Research, Frederick, MD 21702, USA

* Correspondence: beutlerj@mail.nih.gov (J.A.B.); mahmut.miski@gmail.com (M.M.)

Abstract: Six new sesquiterpene coumarin ethers turcicanol A (**1**), turcicanol A acetate (**2**), turcicanol B (**3**), turcica ketone (**4**), 11′-dehydrokaratavicinol (**5**), galbanaldehyde (**6**), and one new sulfur containing compound turcicasulphide (**7**) along with thirty-two known secondary metabolites were isolated from root of the endemic species *Ferula turcica* Akalın, Miski & Tuncay through a bioassay-guided isolation approach. The structure of the new compounds was elucidated by spectroscopic analysis and comparison with the literature. Cell growth of colon cancer cell lines (COLO205, HCT116) and kidney cancer cell lines (UO31, A498) was used to guide isolation. Seventeen of the compounds showed significant activity against the cell lines.

* Correspondence:

Dr. John A. Beutler; e-mail: beutlerj@mail.nih.gov

Prof. Dr. Mahmut Miski; e-mail: mahmut.miski@gmail.com

Contents **Page**

Figure S1 Pure compounds isolation chart of *F. turcica* 5
Figure S2 LC-MS result of *F. turcica* DCM extract 6
Figure S3 Structures of the compounds 7
Figure S4 ^{1}H-NMR spectrum (600 MHz, $CDCl_3$) of turcicanol A (**1**) 8
Figure S5 ^{13}C-NMR spectrum (150 MHz, $CDCl_3$) of turcicanol A (**1**) 8
Figure S6 COSY spectrum of turcicanol A (**1**) 9
Figure S7 HSQC spectrum of turcicanol A (**1**) 9
Figure S8 HMBC spectrum of turcicanol A (**1**) 10
Figure S9 NOESY spectrum of turcicanol A (**1**) 10
Figure S10 UV spectrum (MeOH) of turcicanol A (**1**) 11
Figure S11 IR spectrum (NaCl) of turcicanol A (**1**) 11
Figure S12 HRMS of turcicanol A (**1**) 12
Figure S13 ^{1}H-NMR spectrum (600 MHz, $CDCl_3$) of turcicanol A acetate (**2**) 13
Figure S14 ^{13}C-NMR spectrum (150 MHz, $CDCl_3$) of turcicanol A acetate (**2**) 13
Figure S15 COSY spectrum of turcicanol A acetate (**2**) 14
Figure S16 HSQC spectrum of turcicanol A acetate (**2**) 14
Figure S17 HMBC spectrum of turcicanol A acetate (**2**) 15
Figure S18 NOESY spectrum of turcicanol A acetate (**2**) 15
Figure S19 UV spectrum (MeOH) of turcicanol A acetate (**2**) 16
Figure S20 IR spectrum (NaCl) of turcicanol A acetate (**2**) 16
Figure S21 HRMS of turcicanol A acetate (**2**) 17
Figure S22 ^{1}H-NMR spectrum (600 MHz, $CDCl_3$) of turcicanol B (**3**) 18
Figure S23 ^{13}C-NMR spectrum (150 MHz, $CDCl_3$) of turcicanol B (**3**) 18
Figure S24 COSY spectrum of turcicanol B (**3**) 19
Figure S25 HSQC spectrum of turcicanol B (**3**) 19
Figure S26 HMBC spectrum of turcicanol B (**3**) 20
Figure S27 NOESY spectrum of turcicanol B (**3**) 20
Figure S28 UV spectrum (MeOH) of turcicanol B (**3**) 21
Figure S29 IR spectrum (NaCl) of turcicanol B (**3**) 21
Figure S30 HRMS of turcicanol B (**3**) 22
Figure S31 ^{1}H-NMR spectrum (600 MHz, $CDCl_3$) of turcica ketone (**4**) 23
Figure S32 ^{13}C-NMR spectrum (150 MHz, $CDCl_3$) of turcica ketone (**4**) 23
Figure S33 COSY spectrum of turcica ketone (**4**) 24
Figure S34 HSQC spectrum of turcica ketone (**4**) 24
Figure S35 HMBC spectrum of turcica ketone (**4**) 25

Figure S36 NOESY spectrum of turcica ketone (**4**) 25
Figure S37 UV spectrum (MeOH) of turcica ketone (**4**) 26
Figure S38 IR spectrum (NaCl) of turcica ketone (**4**) 26
Figure S39 HRMS of turcica ketone (**4**) 27
Figure S40 ^{1}H-NMR spectrum (600 MHz, $CDCl_3$) of 11'-dehydrokaratavicinol (**5**) 28
Figure S41 ^{13}C-NMR spectrum (150 MHz, $CDCl_3$) of 11'-dehydrokaratavicinol (**5**) 28
Figure S42 COSY spectrum of 11'-dehydrokaratavicinol (**5**) 29
Figure S43 HSQC spectrum of 11'-dehydrokaratavicinol (**5**) 29
Figure S44 HMBC spectrum of 11'-dehydrokaratavicinol (**5**) 30
Figure S45 NOESY spectrum of 11'-dehydrokaratavicinol (**5**) 30
Figure S46 UV spectrum (MeOH) of 11'-dehydrokaratavicinol (**5**) 31
Figure S47 IR spectrum (NaCl) of 11'-dehydrokaratavicinol (**5**) 31
Figure S48 HRMS of 11'-dehydrokaratavicinol (**5**) 32
Figure S49 ^{1}H-NMR spectrum (600 MHz, $CDCl_3$) of galbanaldehyde (**6**) 33
Figure S50 ^{13}C-NMR spectrum (150 MHz, $CDCl_3$) of galbanaldehyde (**6**) 33
Figure S51 COSY spectrum of galbanaldehyde (**6**) 34
Figure S52 HSQC spectrum of galbanaldehyde (**6**) 34
Figure S53 HMBC spectrum of galbanaldehyde (**6**) 35
Figure S54 NOESY spectrum of galbanaldehyde (**6**) 35
Figure S55 UV spectrum (MeOH) of galbanaldehyde (**6**) 36
Figure S56 IR spectrum (NaCl) of galbanaldehyde (**6**) 36
Figure S57 HRMS of galbanaldehyde (**6**) 37
Figure S58 ^{1}H-NMR spectrum (600 MHz, $CDCl_3$) of turcicasulphide (**7**) 38
Figure S59 ^{13}C-NMR spectrum (150 MHz, $CDCl_3$) of turcicasulphide (**7**) 38
Figure S60 COSY spectrum of turcicasulphide (**7**) 39
Figure S61 HSQC spectrum of turcicasulfide (**7**) 39
Figure S62 HMBC spectrum of turcicasulphide (**7**) 40
Figure S63 NOESY spectrum of turcicasulphide (**7**) 40
Figure S64 UV spectrum (MeOH) of turcicasulphide (**7**) 41
Figure S65 IR spectrum (NaCl) of turcicasulphide (**7**) 41
Figure S66 HRMS of turcicasulphide (**7**) 42
Figure S67 ^{1}H-NMR spectrum (600 MHz, $CDCl_3$) of conferol (**8**) 43
Figure S68 ^{1}H-NMR spectrum (600 MHz, $CDCl_3$) of colladonin (**9**) 43
Figure S69 ^{1}H-NMR spectrum (600 MHz, $CDCl_3$) of badrakemin (**10**) 44
Figure S70 ^{1}H-NMR spectrum (600 MHz, CDCl3) of badrakemin acetate (**11**) 44
Figure S71 ^{1}H-NMR spectrum (600 MHz, $CDCl_3$) of badrakemone (**12**) 45
Figure S72 ^{1}H-NMR spectrum (600 MHz, $CDCl_3$) of samarcandin acetate (**13**) 45
Figure S73 ^{1}H-NMR spectrum (600 MHz, $CDCl_3$) of deacetylkellerin (**14**) 46
Figure S74 ^{1}H-NMR spectrum (600 MHz, $CDCl_3$) of kellerin (**15**) 46
Figure S75 ^{1}H-NMR spectrum (600 MHz, $CDCl_3$) of ferukrin (**16**) 47

Figure S76 ^{1}H-NMR spectrum (600 MHz, $CDCl_3$) of ferukrin acetate (**17**) 47
Figure S77 ^{1}H-NMR spectrum (600 MHz, $CDCl_3$) of ferukrinone (**18**) 48
Figure S78 ^{1}H-NMR spectrum (600 MHz, $CDCl_3$) of fepaldin (**19**) 48
Figure S79 ^{1}H-NMR spectrum (600 MHz, $CDCl_3$) of gummosin (**20**) 49
Figure S80 ^{1}H-NMR spectrum (600 MHz, $CDCl_3$) of gummosin acetate (**21**) 49
Figure S81 ^{1}H-NMR spectrum (600 MHz, $CDCl_3$) of mogoltadone (**22**) 50
Figure S82 ^{1}H-NMR spectrum (600 MHz, $CDCl_3$) of farnesiferol A (**23**) 50
Figure S83 ^{1}H-NMR spectrum (600 MHz, $CDCl_3$) of farnesiferol A acetate (**24**) 51
Figure S84 ^{1}H-NMR spectrum (600 MHz, $CDCl_3$) of farnesiferol B (**25**) 51
Figure S85 ^{1}H-NMR spectrum (600 MHz, $CDCl_3$) of kopeolin (**26**) 52
Figure S86 ^{1}H-NMR spectrum (600 MHz, $CDCl_3$) of galbanic acid (**27**) 52
Figure S87 ^{1}H-NMR spectrum (600 MHz, $CDCl_3$) of kamolone (**28**) 53
Figure S88 ^{1}H-NMR spectrum (600 MHz, $CDCl_3$) of umbelliprenin (**29**) 53
Figure S89 ^{1}H-NMR spectrum (600 MHz, $CDCl_3$) of 10', 11'-epoxyumbelliprenin (**30**) 54
Figure S90 ^{1}H-NMR spectrum (600 MHz, $CDCl_3$) of karatavikin (**31**) 54
Figure S91 ^{1}H-NMR spectrum (600 MHz, $CDCl_3$) of karatavicinol (**32**) 55
Figure S92 ^{1}H-NMR spectrum (600 MHz, $CDCl_3$) of 10′-acetylkaratavicinol (**33**) 55
Figure S93 ^{1}H-NMR spectrum (600 MHz, $CDCl_3$) of 2-epihelmanticine (**34**) 56
Figure S94 ^{1}H-NMR spectrum (600 MHz, $CDCl_3$) of laserine (**35**) 56
Figure S95 ^{1}H-NMR spectrum (600 MHz, $CDCl_3$) of crocatone (**36**) 57
Figure S96 ^{1}H-NMR spectrum (600 MHz, $CDCl_3$) of falcarindiol (**37**) 57
Figure S97 ^{1}H-NMR spectrum (600 MHz, $CDCl_3$) of persicasulphide A (**38**) 58
Figure S98 ^{1}H-NMR spectrum (600 MHz, $CDCl_3$) of persicasulphide C (**39**) 58
Figure S99 Cytotoxic activity graphics of pure compounds -1 59
Figure S100 Cytotoxic activity graphics of pure compounds -2 60
Figure S101 Cytotoxic activity graphics of pure compounds -3 61
Figure S102 Cytotoxic activity graphics of pure compounds -4 62
Figure S103 Cytotoxic activity graphics of pure compounds -5 63
Figure S104 Cytotoxic activity graphics of pure compounds -6 64
Figure S105 Cytotoxic activity graphics of pure compounds -7 65
Figure S106 Cytotoxic activity graphics of pure compounds -8 66
Figure S107 Cytotoxic activity graphics of pure compounds -9 67
Figure S108 Cytotoxic activity graphics of pure compounds -10 68

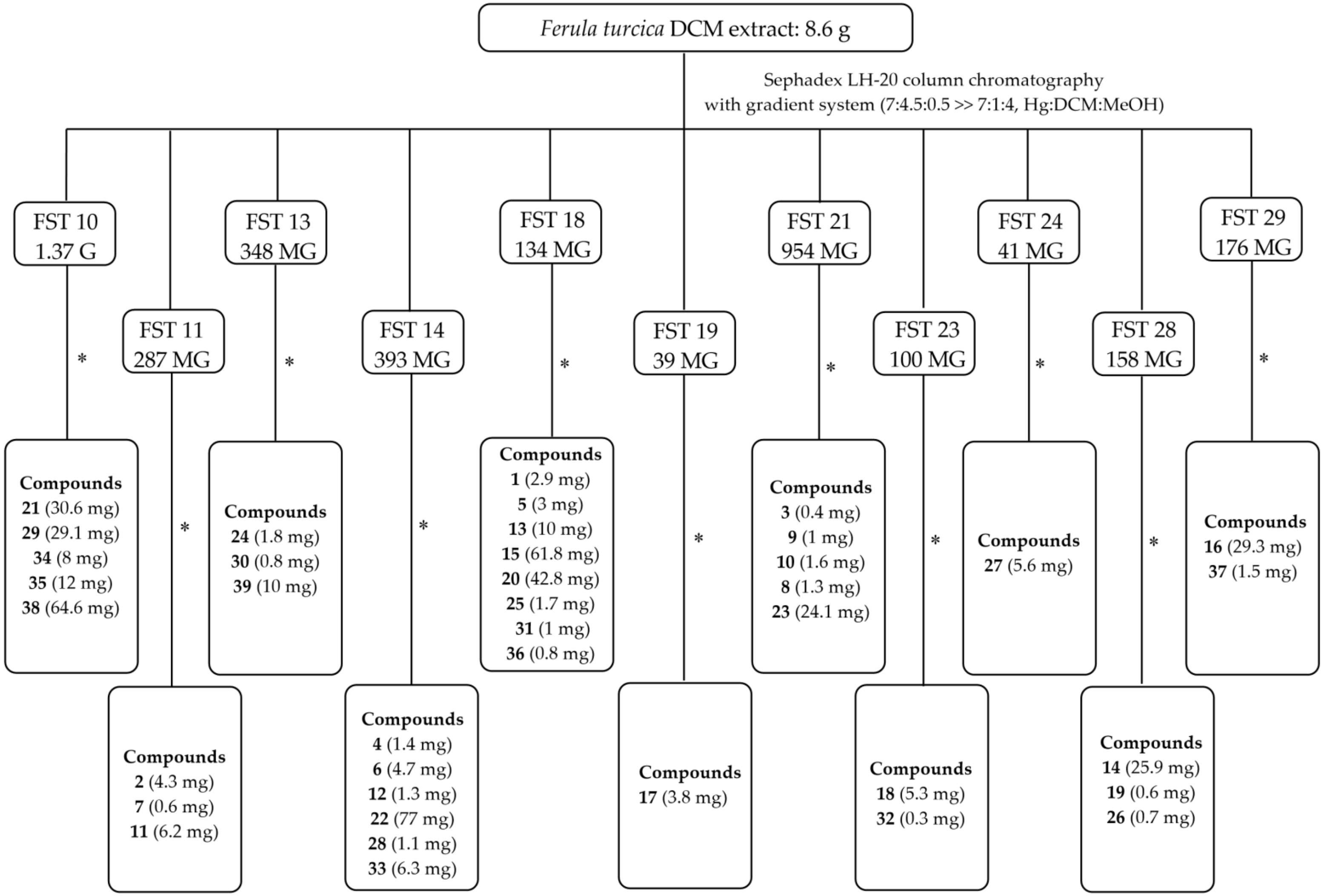

*Preparative HPLC with gradient system (60:100 >> 100:0, ACN:H_2O) and C18 reverse phase column

Figure S1 Pure compounds isolation chart of *F. turcica*

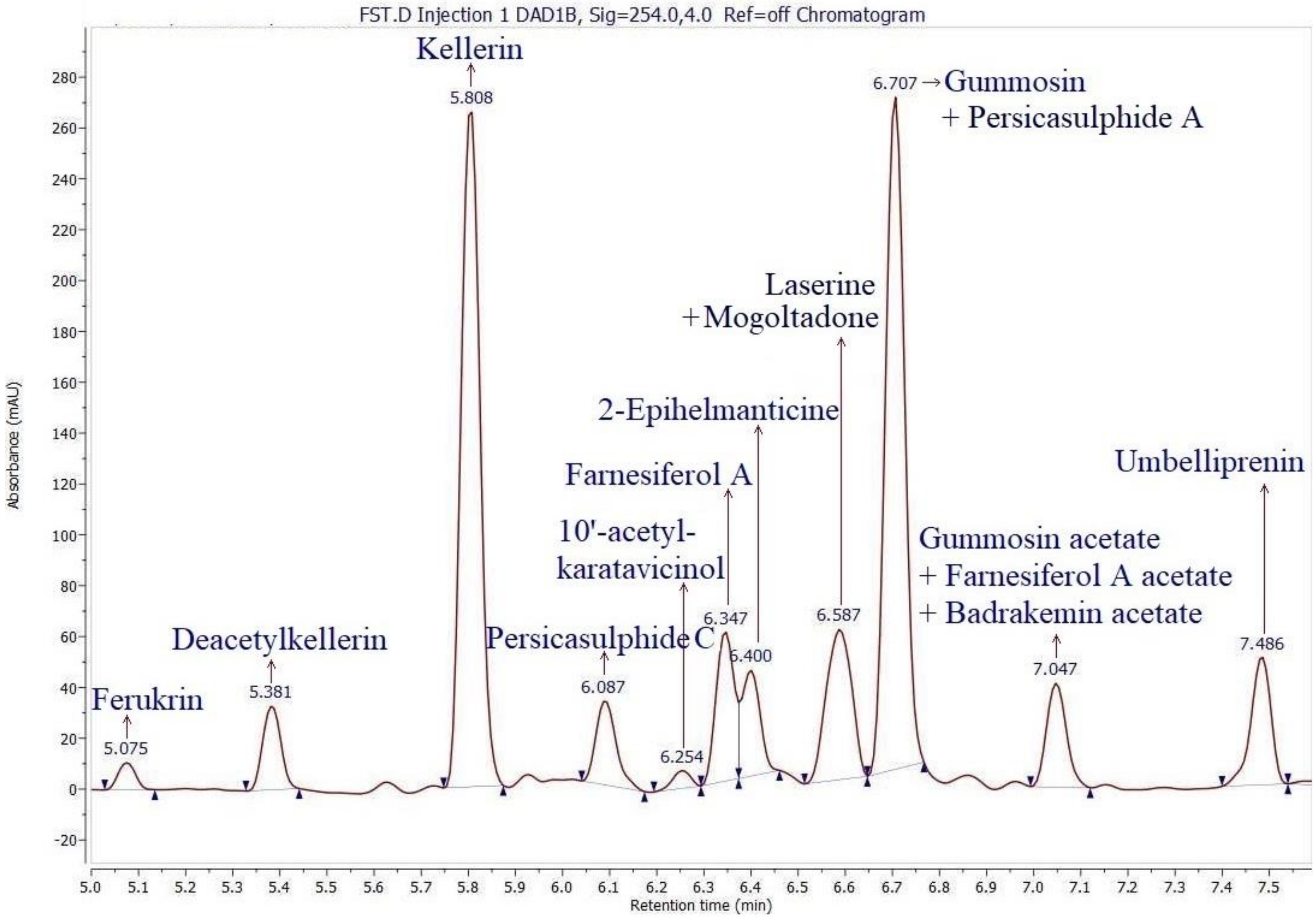

Figure S2 LC-MS result of *F. turcica* DCM extract

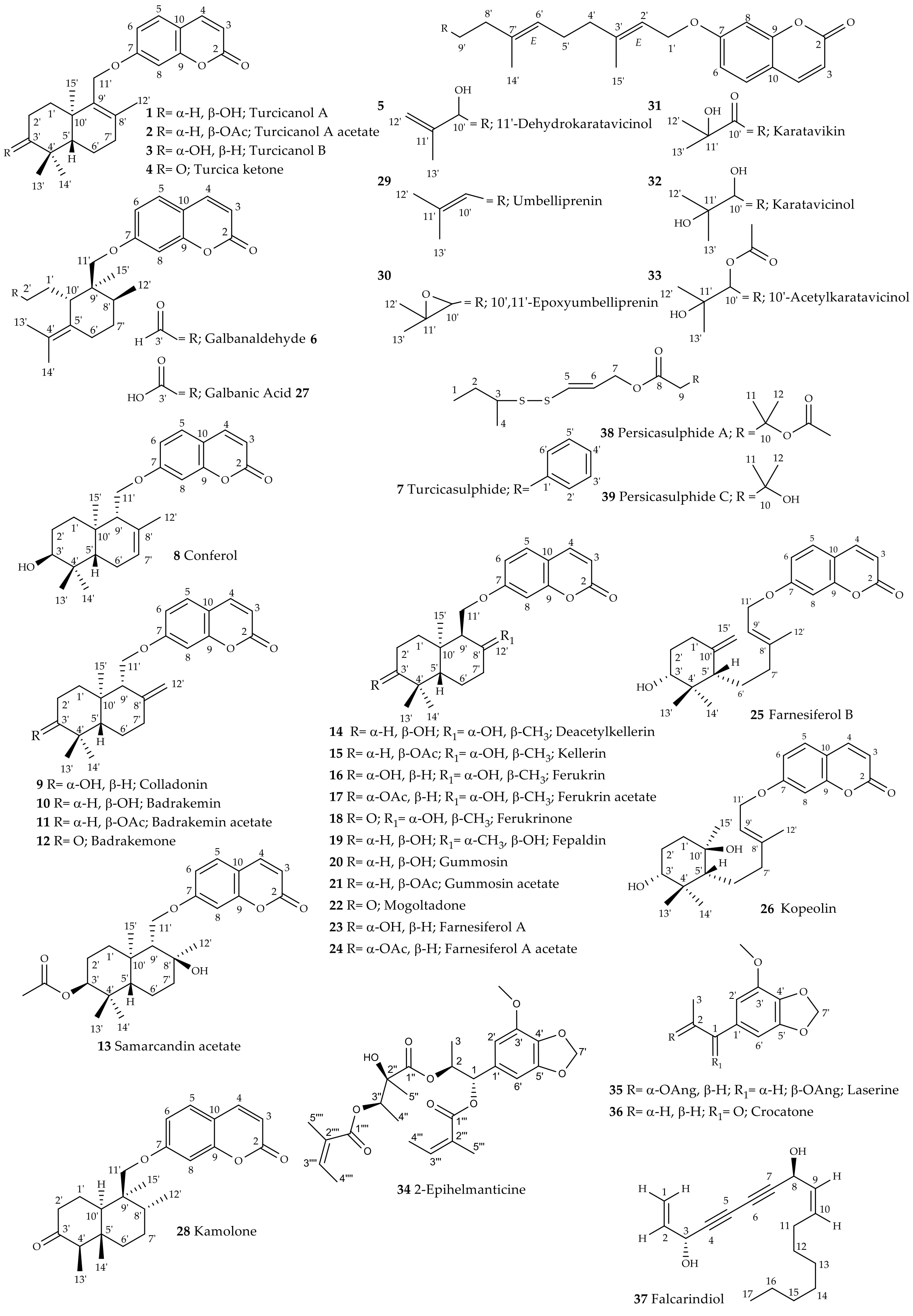

Figure S3 Structures of the compounds

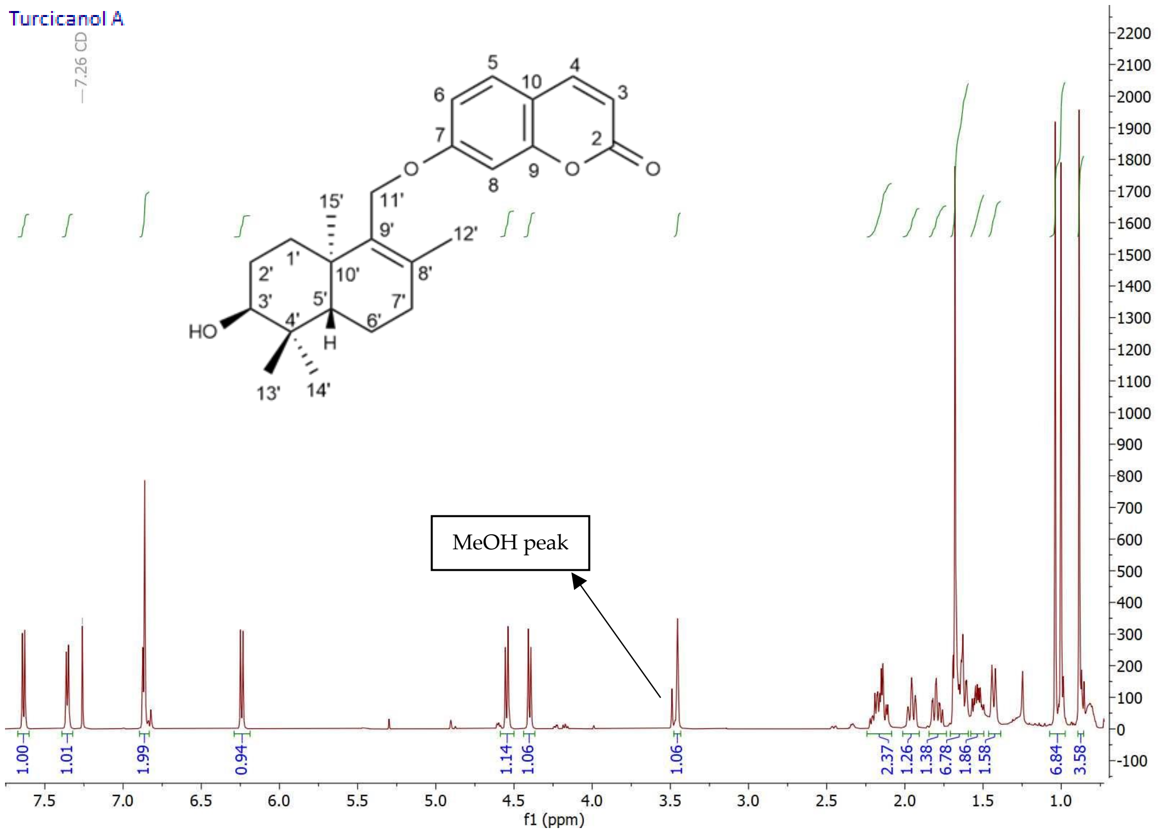

Figure S4 ^{1}H-NMR spectrum (600 MHz, $CDCl_3$) of turcicanol A (**1**)

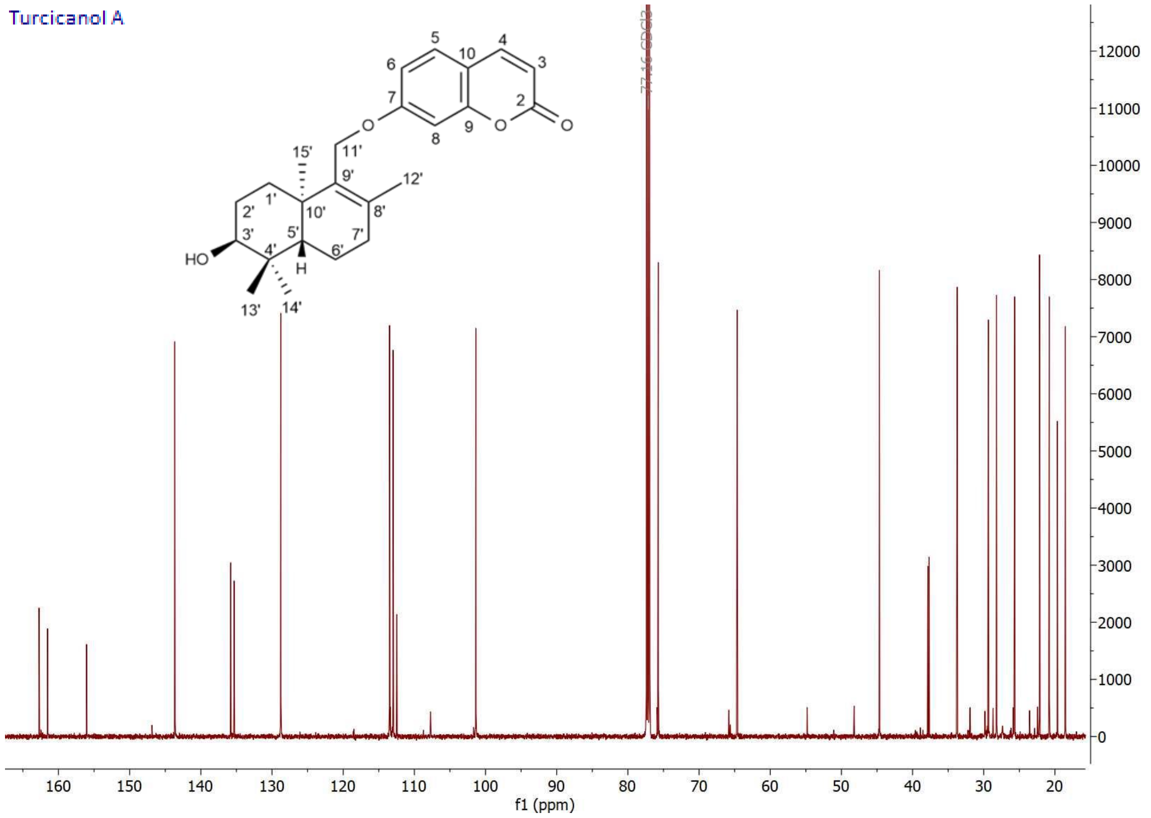

Figure S5 ^{13}C-NMR spectrum (150 MHz, $CDCl_3$) of turcicanol A (**1**)

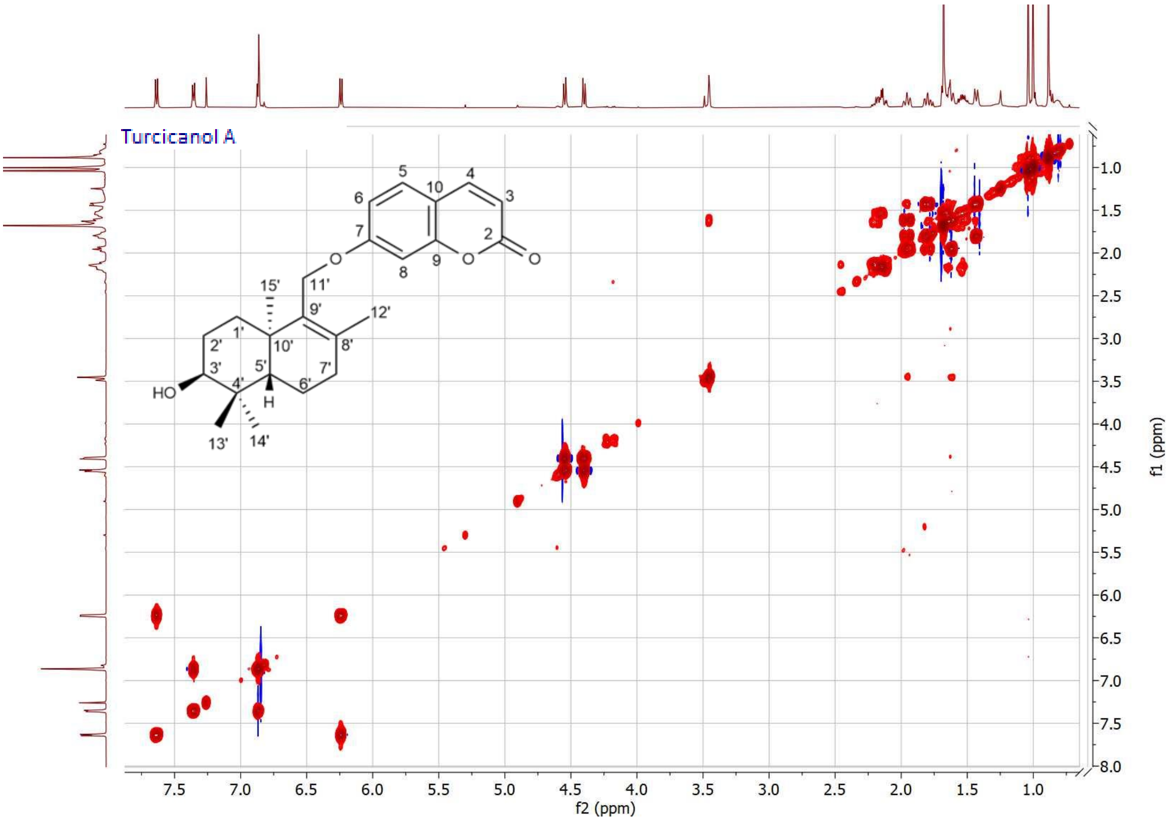

Figure S6 COSY spectrum of turcicanol A (**1**)

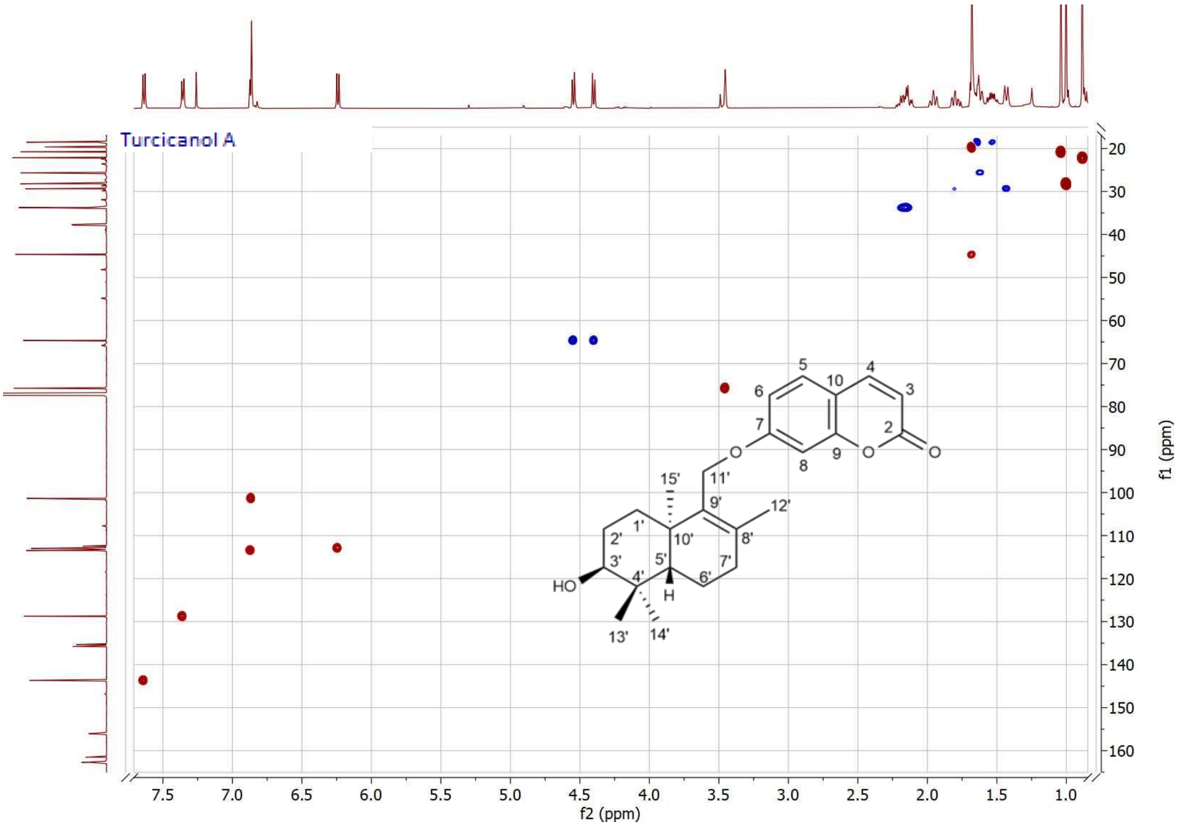

Figure S7 HSQC spectrum of turcicanol A (**1**)

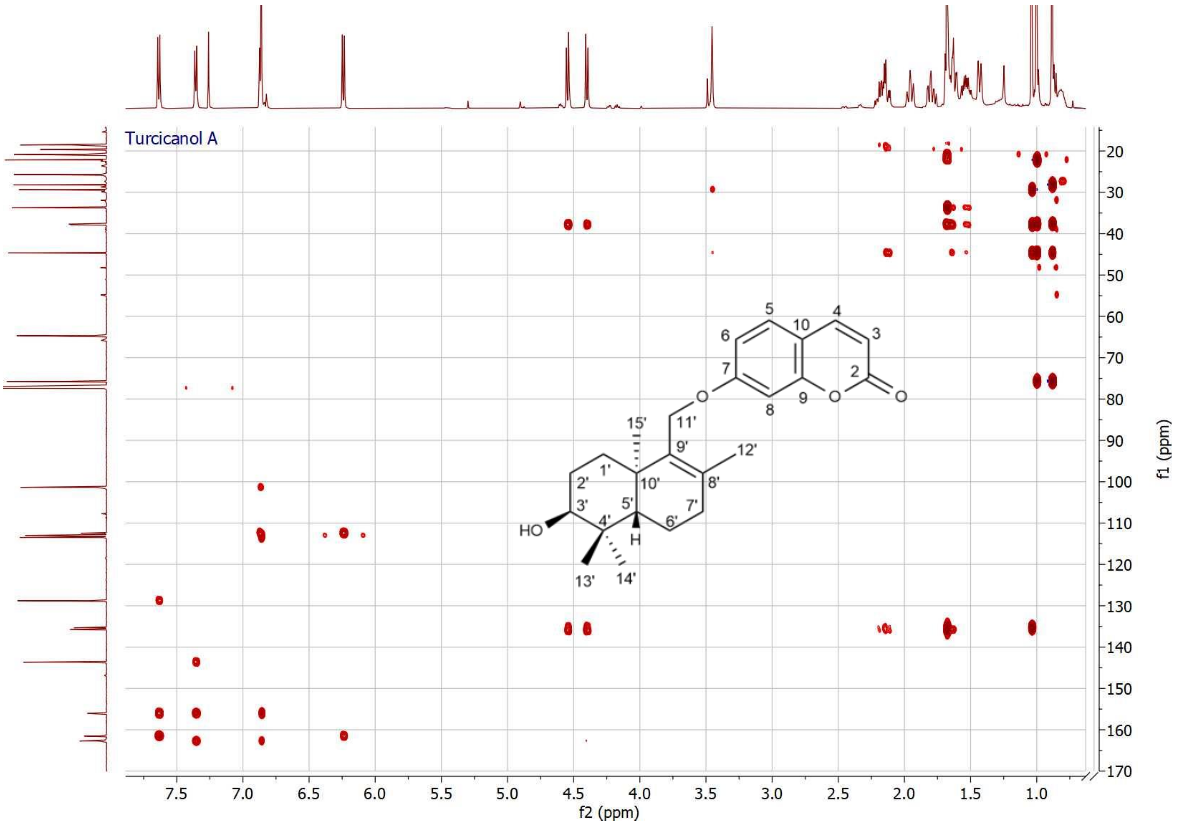

Figure S8 HMBC spectrum of turcicanol A (**1**)

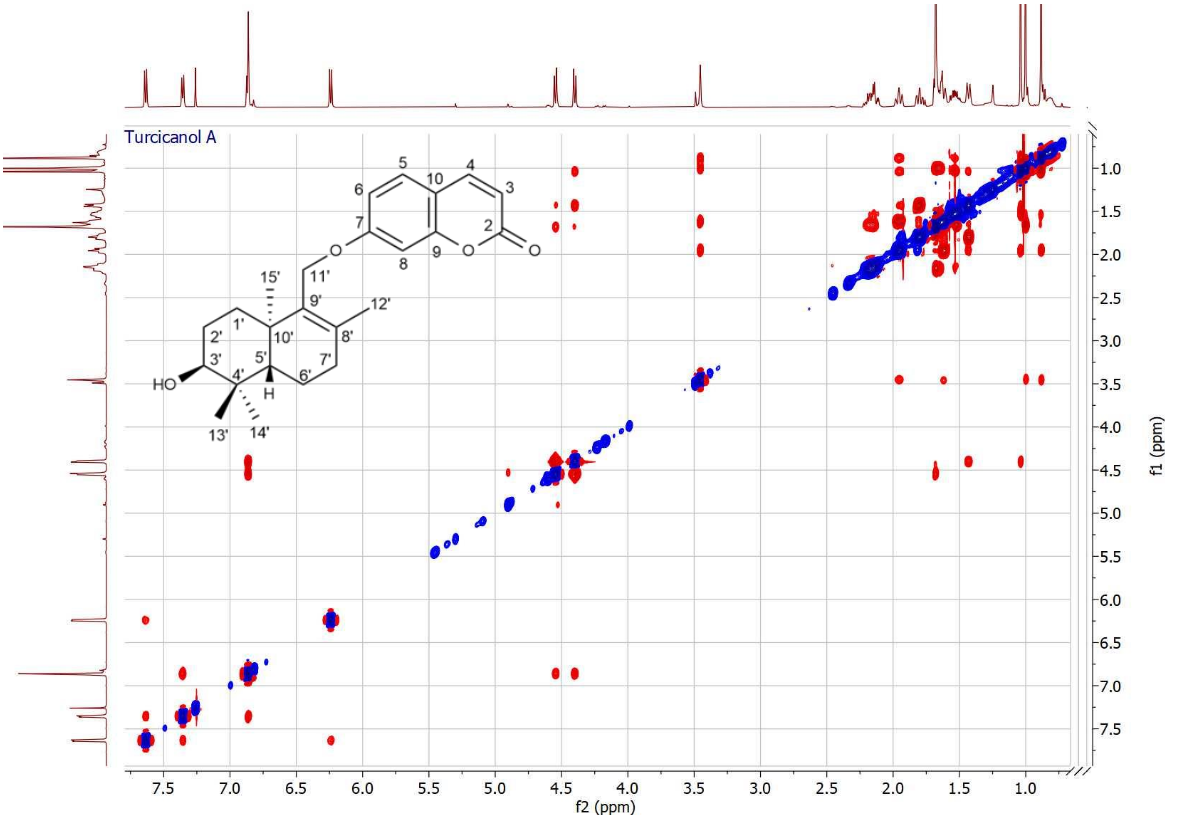

Figure S9 NOESY spectrum of turcicanol A (**1**)

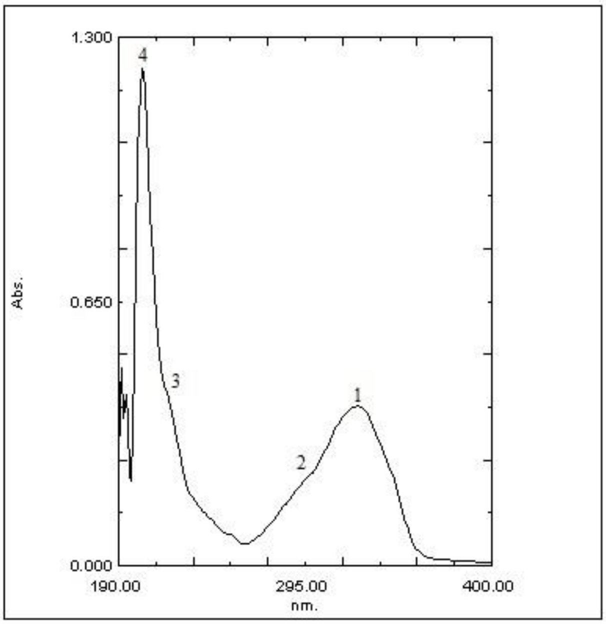

	Wavelength	Absorbance
1	324.00	0.392
2	295.00	0.212
3	218.00	0.410
4	203.00	1.224

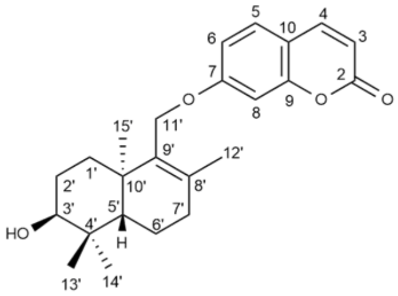

Figure S10 UV spectrum (MeOH) of turcicanol A (**1**)

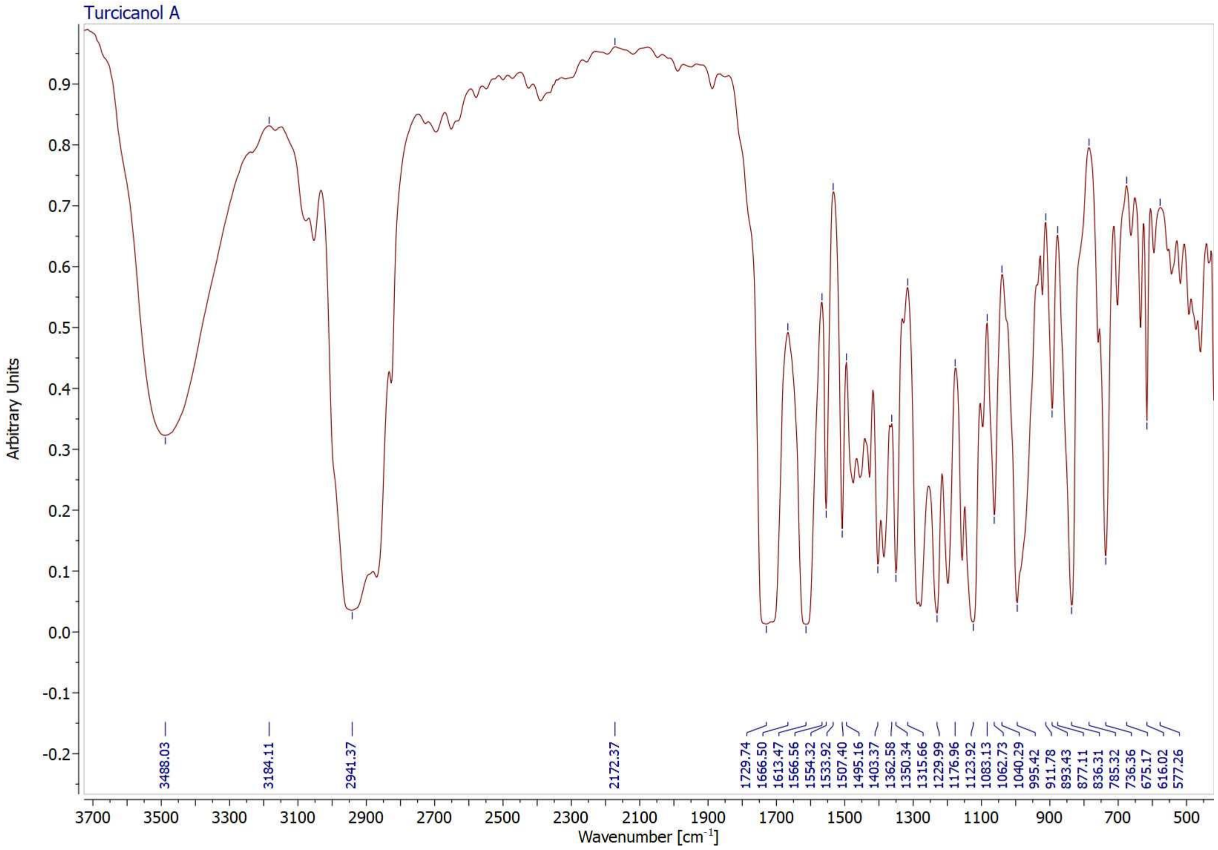

Figure S11 IR spectrum (NaCl) of turcicanol A (**1**)

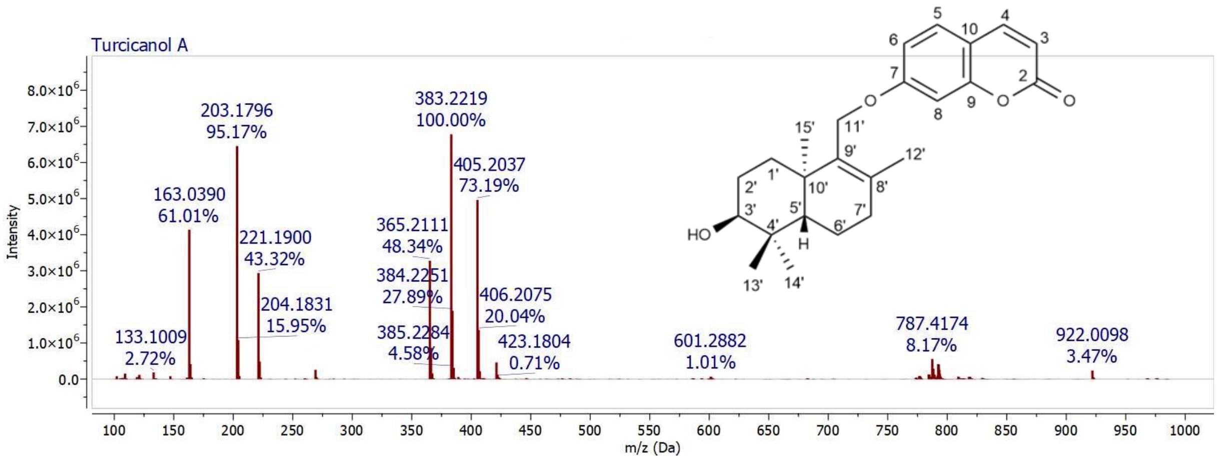

Figure S12 HRMS of turcicanol A (**1**)

m/z $[M+H]^+$ 383.2219 (calculated: 383.2222), $[M+Na]^+$ 405.2037

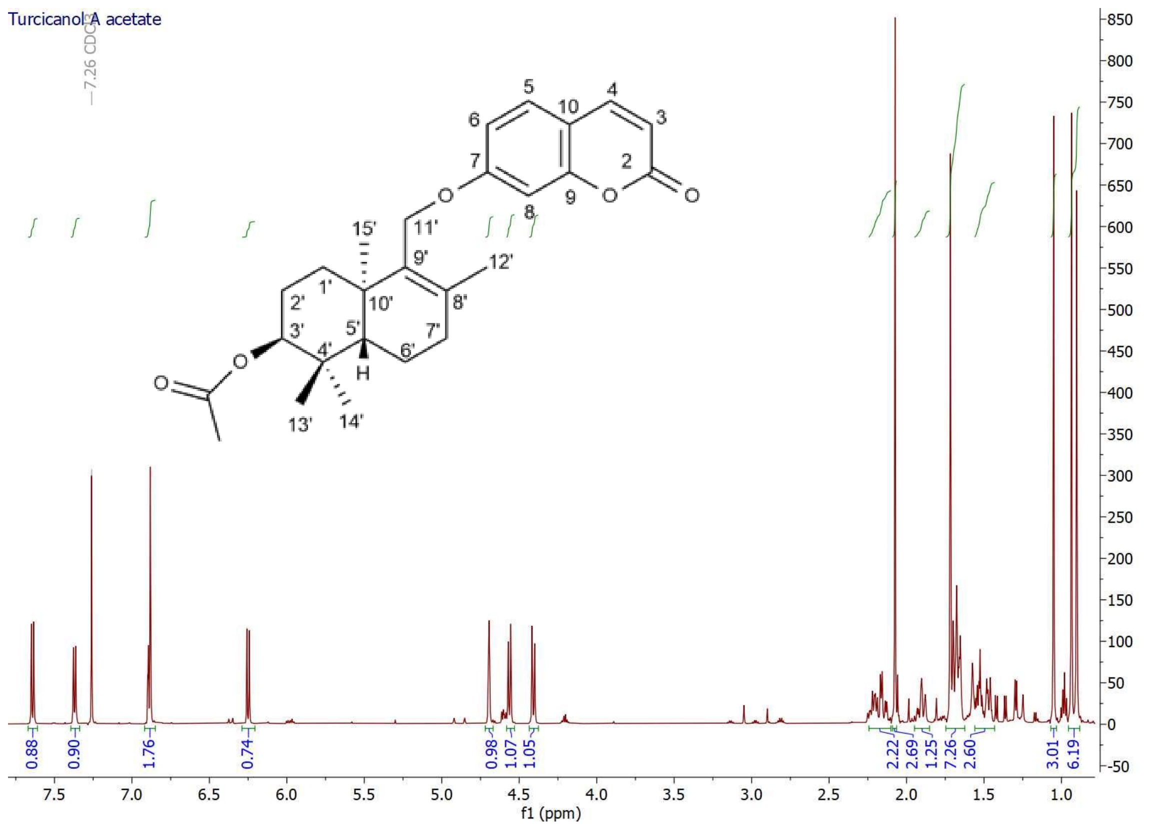

Figure S13 ^{1}H-NMR spectrum (600 MHz, $CDCl_3$) of turcicanol A acetate (**2**)

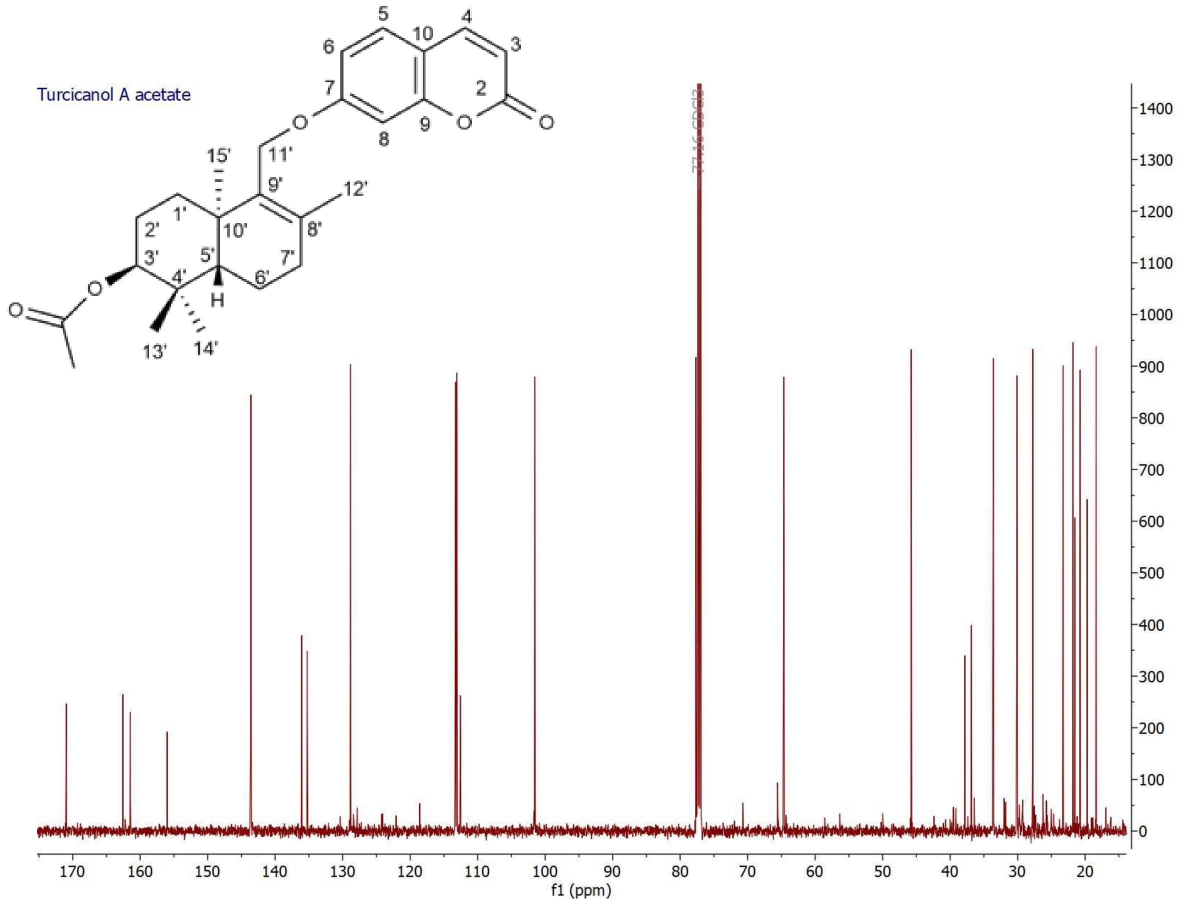

Figure S14 ^{13}C-NMR spectrum (150 MHz, $CDCl_3$) of turcicanol A acetate (**2**)

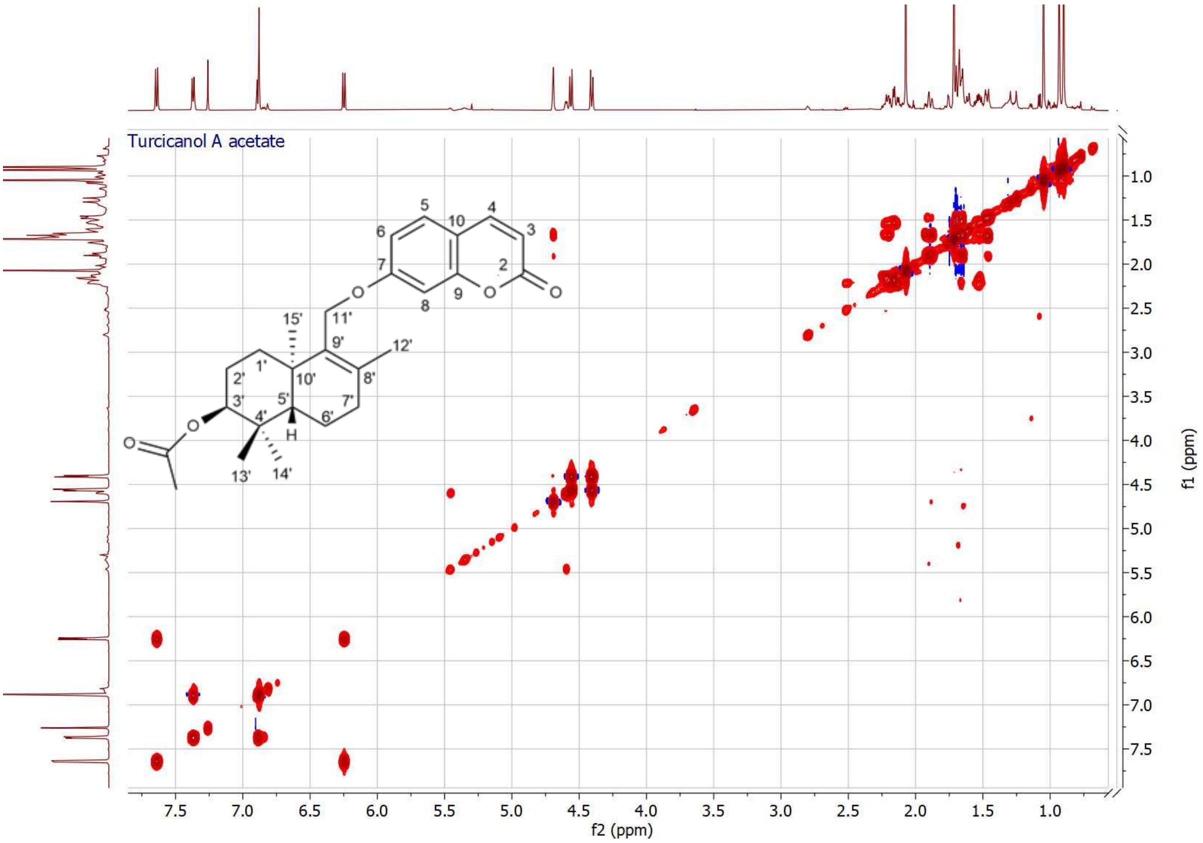

Figure S15 COSY spectrum of turcicanol A acetate (**2**)

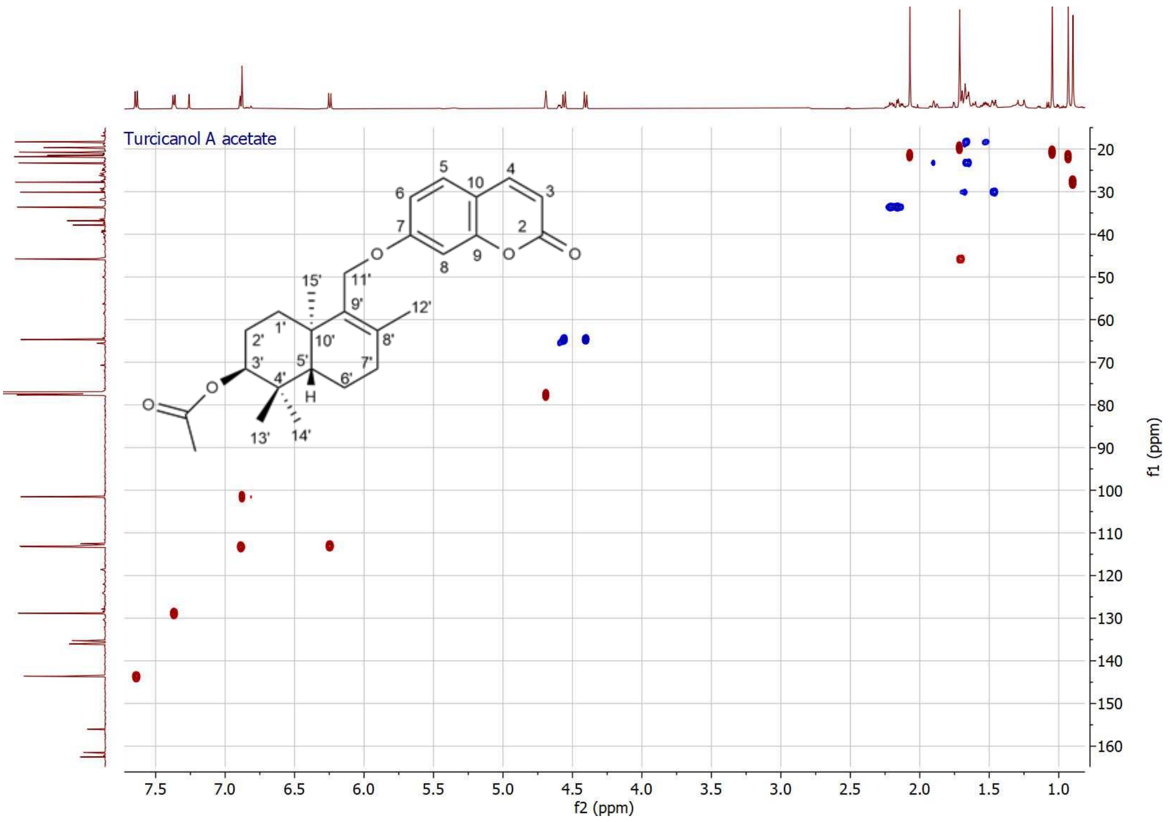

Figure S16 HSQC spectrum of turcicanol A acetate (**2**)

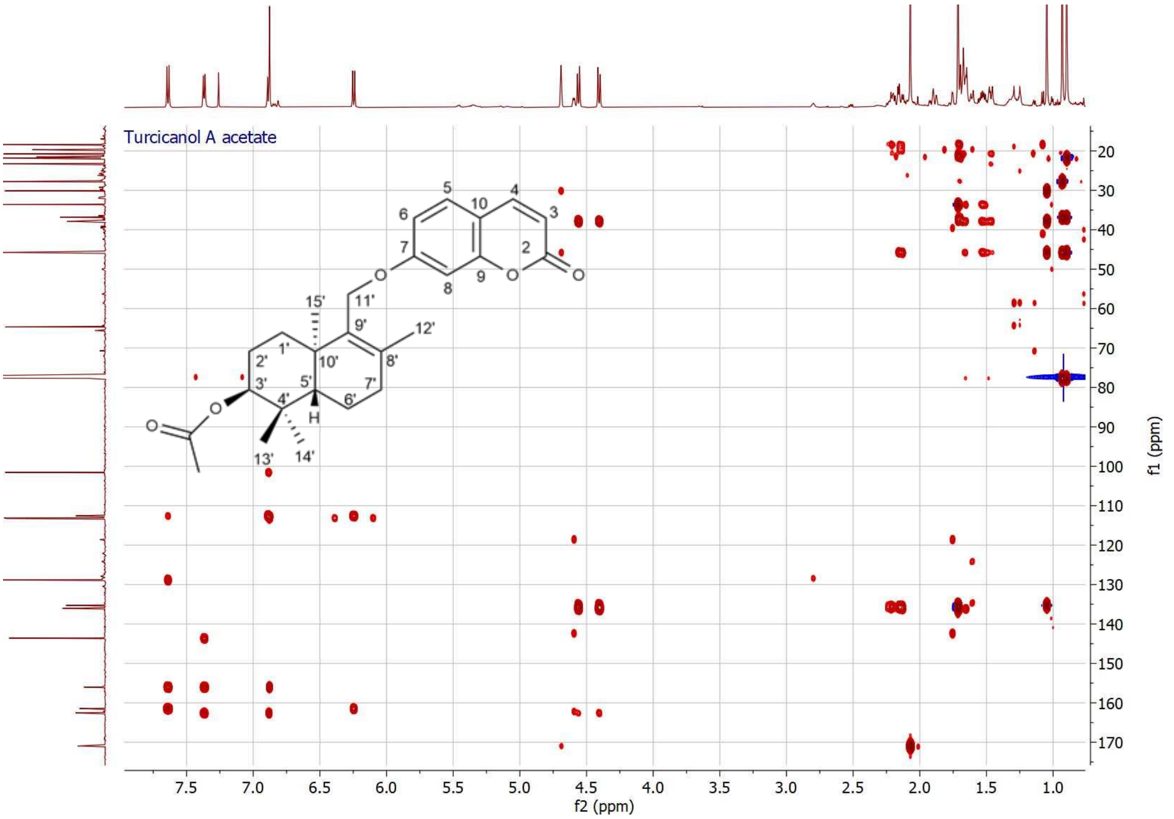

Figure S17 HMBC spectrum of turcicanol A acetate (**2**)

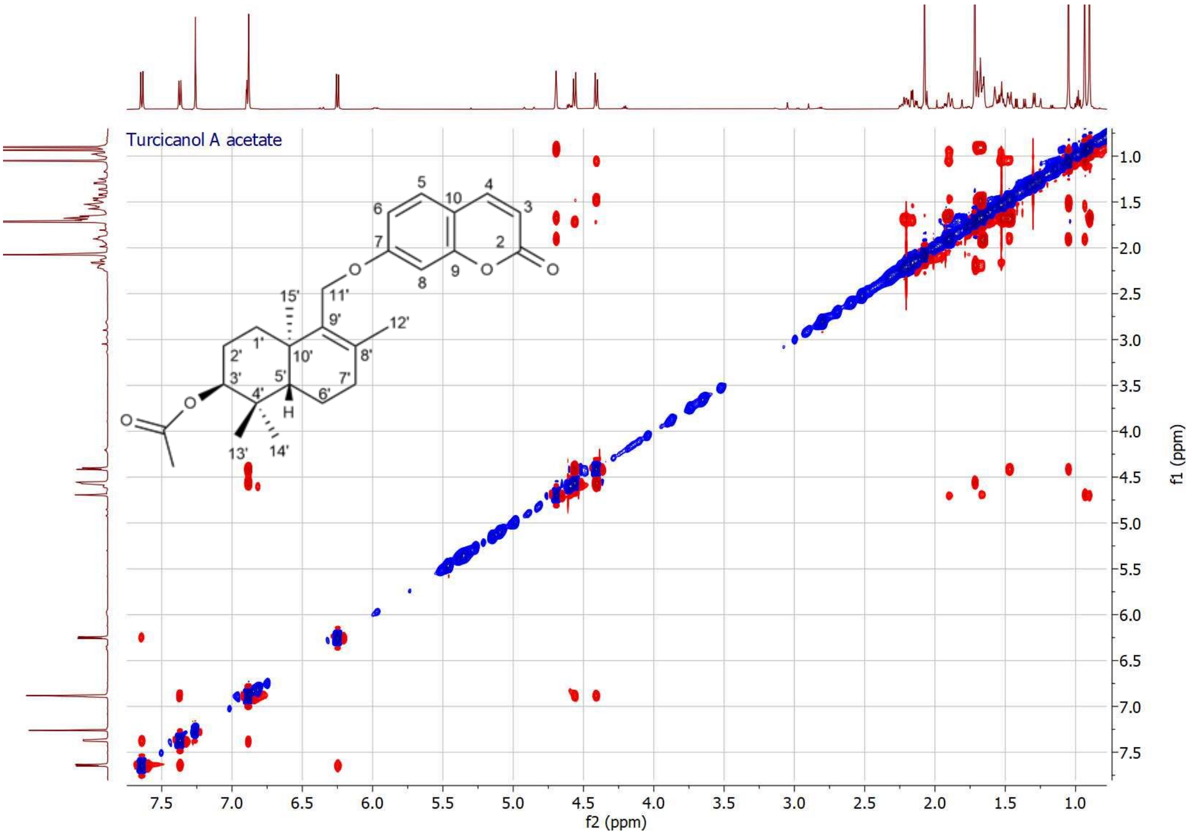

Figure S18 NOESY spectrum of turcicanol A acetate (**2**)

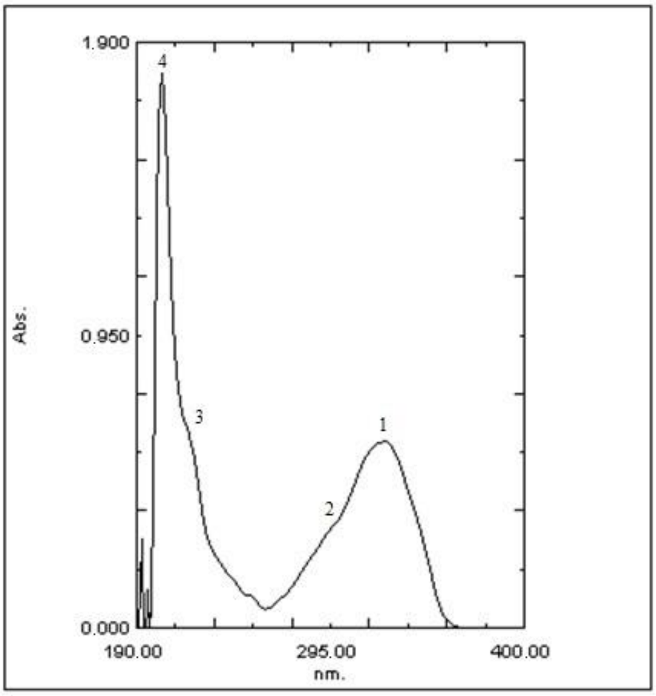

	Wavelength	Absorbance
1	324.00	0.605
2	295.00	0.320
3	219.00	0.601
4	204.00	1.799

Figure S19 UV spectrum (MeOH) of turcicanol A acetate (**2**)

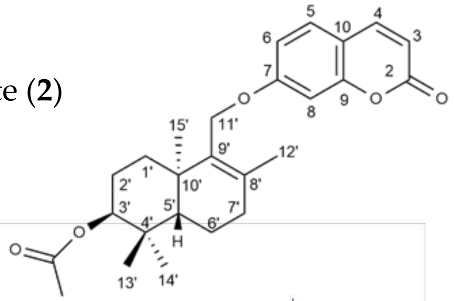

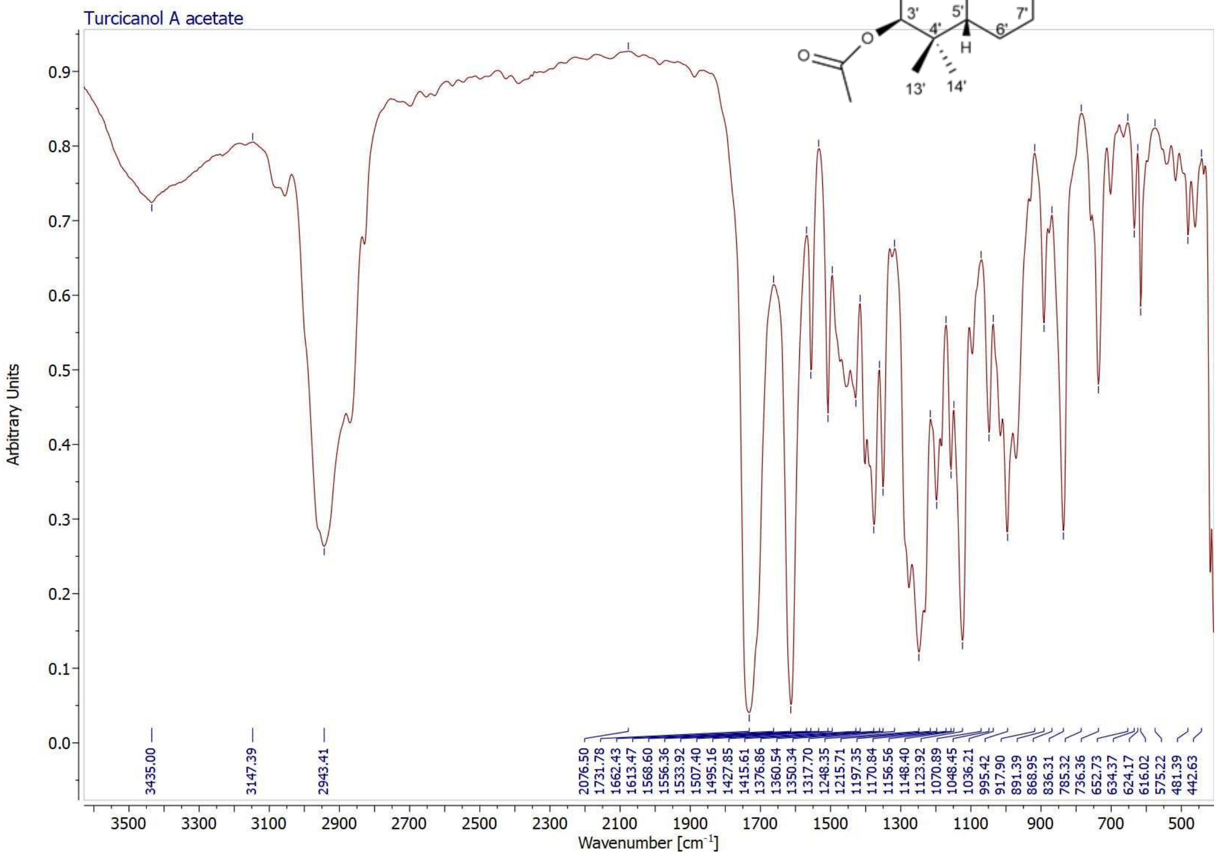

Figure S20 IR spectrum (NaCl) of turcicanol A acetate (**2**)

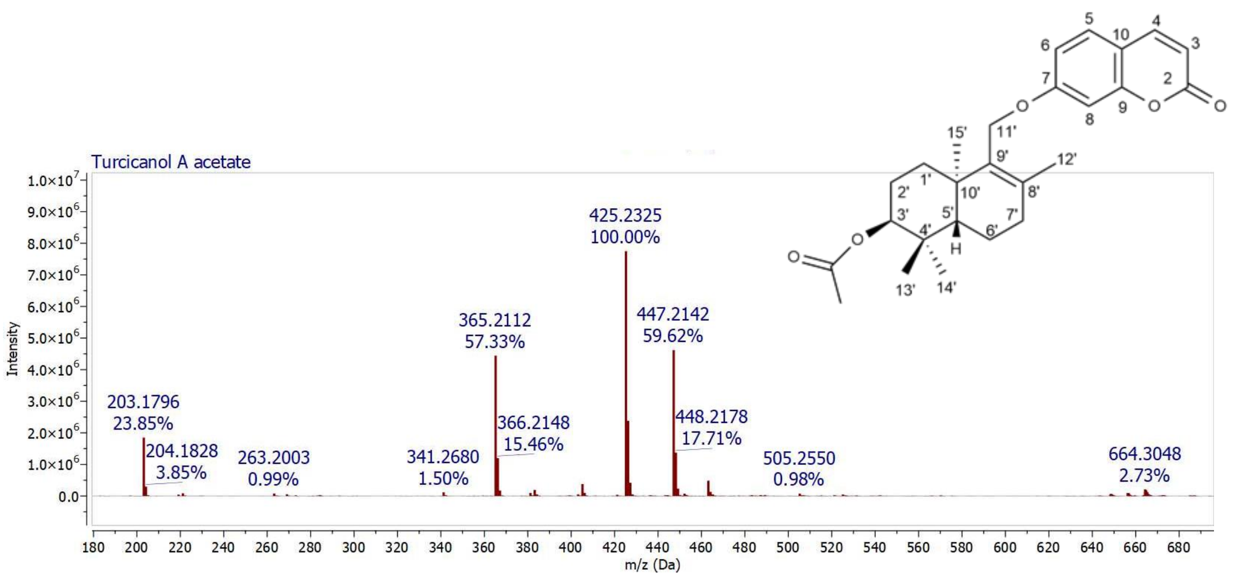

Figure S21 HRMS of turcicanol A acetate (**2**)

m/z $[M+H]^+$ 425.2325 (calculated: 425.2328), $[M+Na]^+$ 447.2142

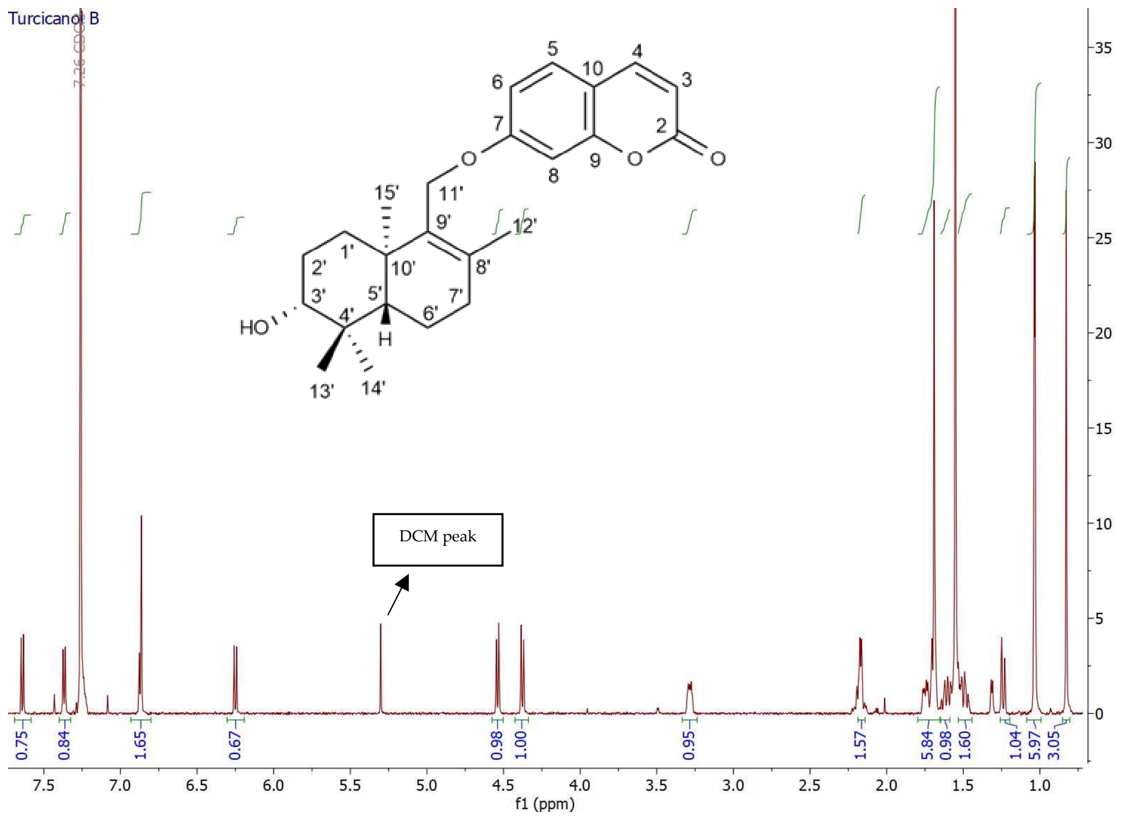

Figure S22 ^{1}H-NMR spectrum (600 MHz, $CDCl_3$) of turcicanol B (**3**)

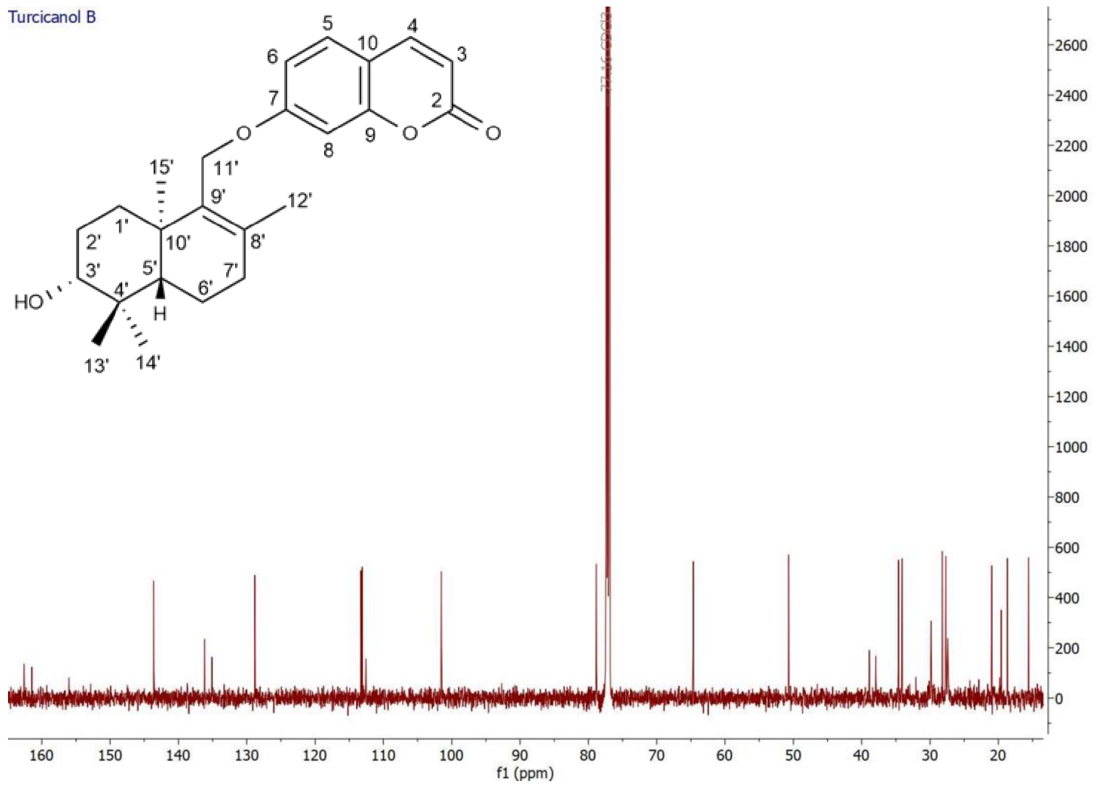

Figure S23 ^{13}C-NMR spectrum (150 MHz, $CDCl_3$) of turcicanol B (**3**)

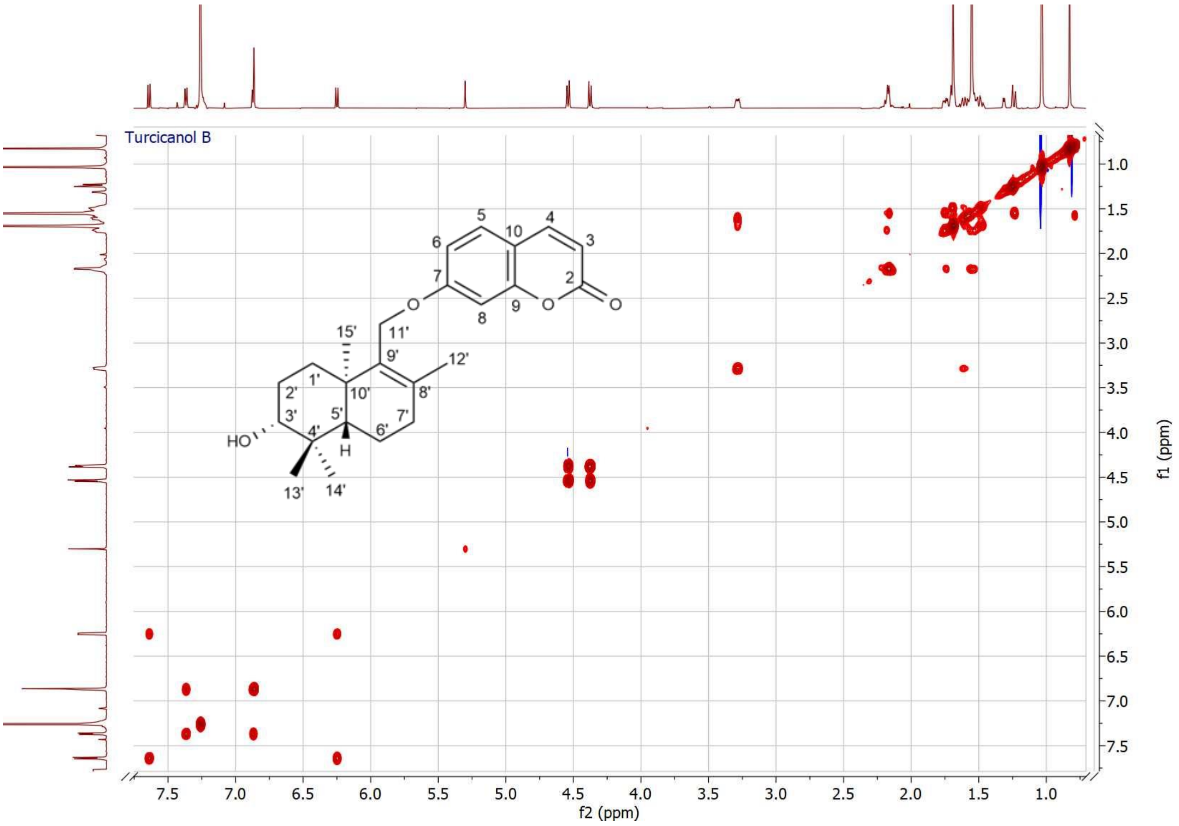

Figure S24 COSY spectrum of turcicanol B (**3**)

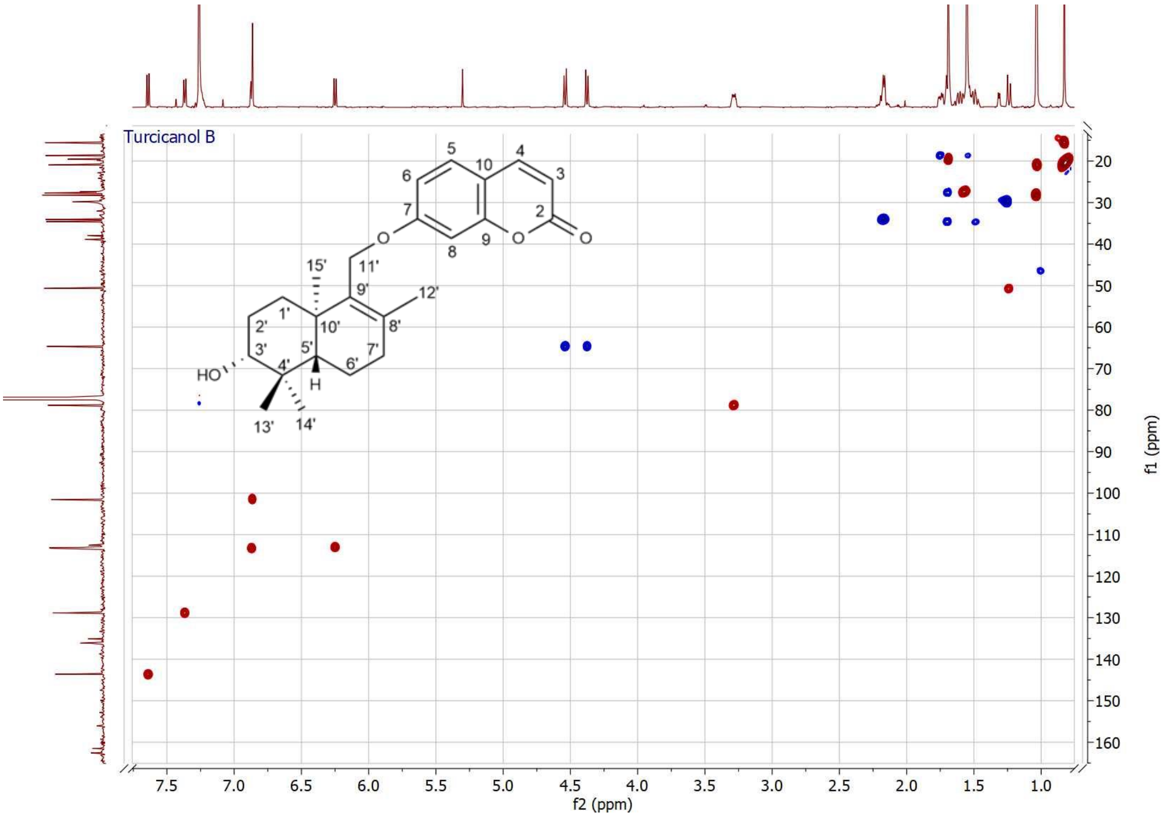

Figure S25 HSQC spectrum of turcicanol B (**3**)

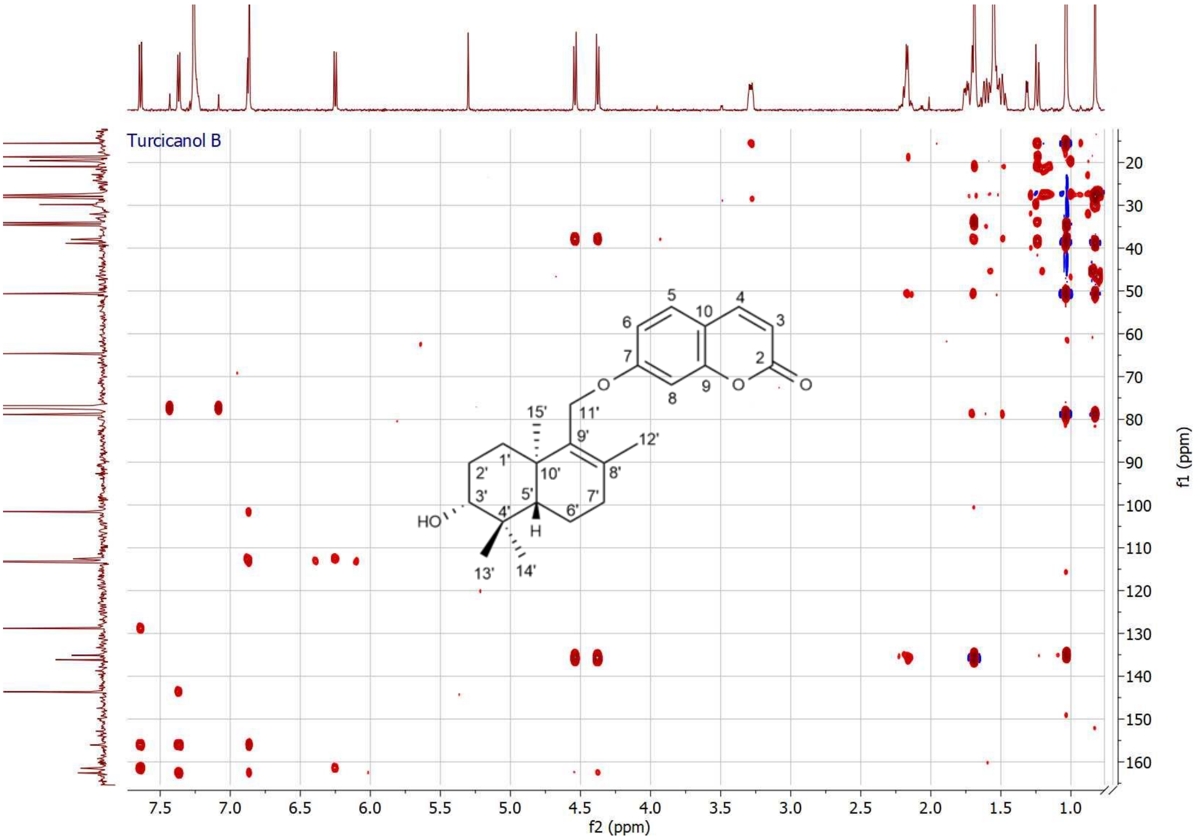

Figure S26 HMBC spectrum of turcicanol B (**3**)

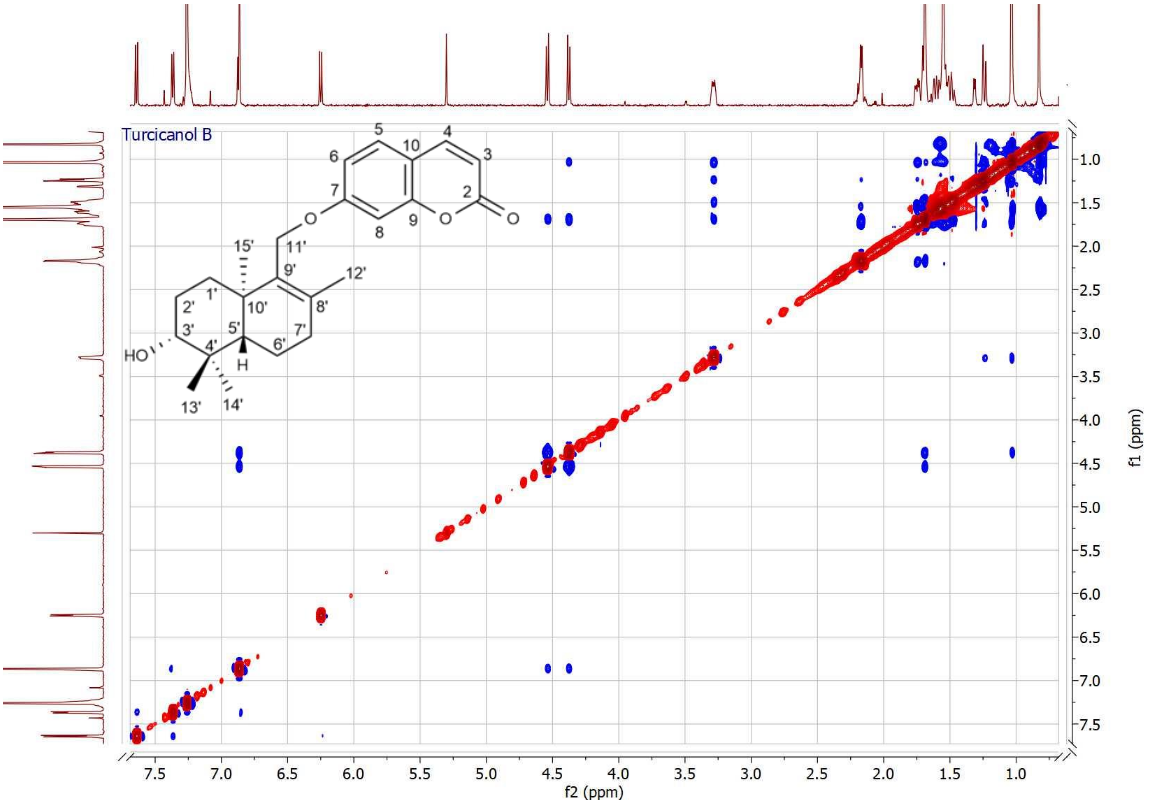

Figure S27 NOESY spectrum of turcicanol B (**3**)

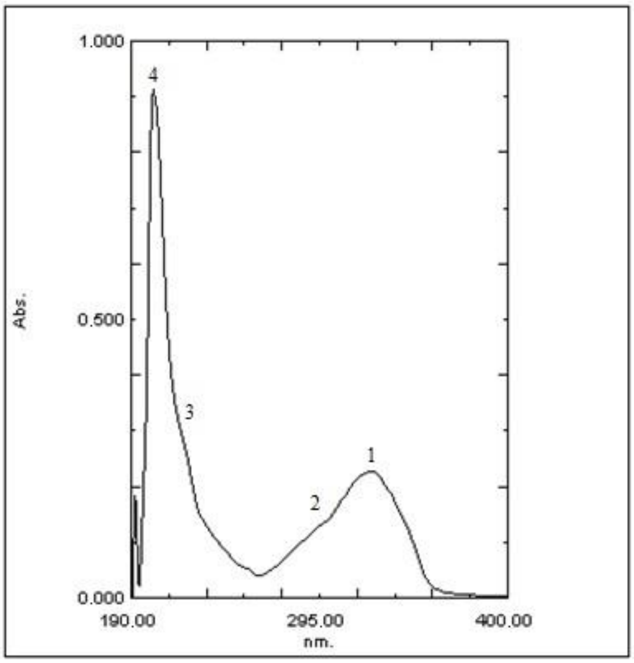

	Wavelength	Absorbance
1	324.00	0.227
2	296.00	0.132
3	220.00	0.275
4	203.00	0.913

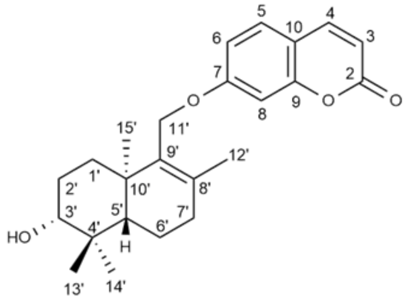

Figure S28 UV spectrum (MeOH) of turcicanol B (**3**)

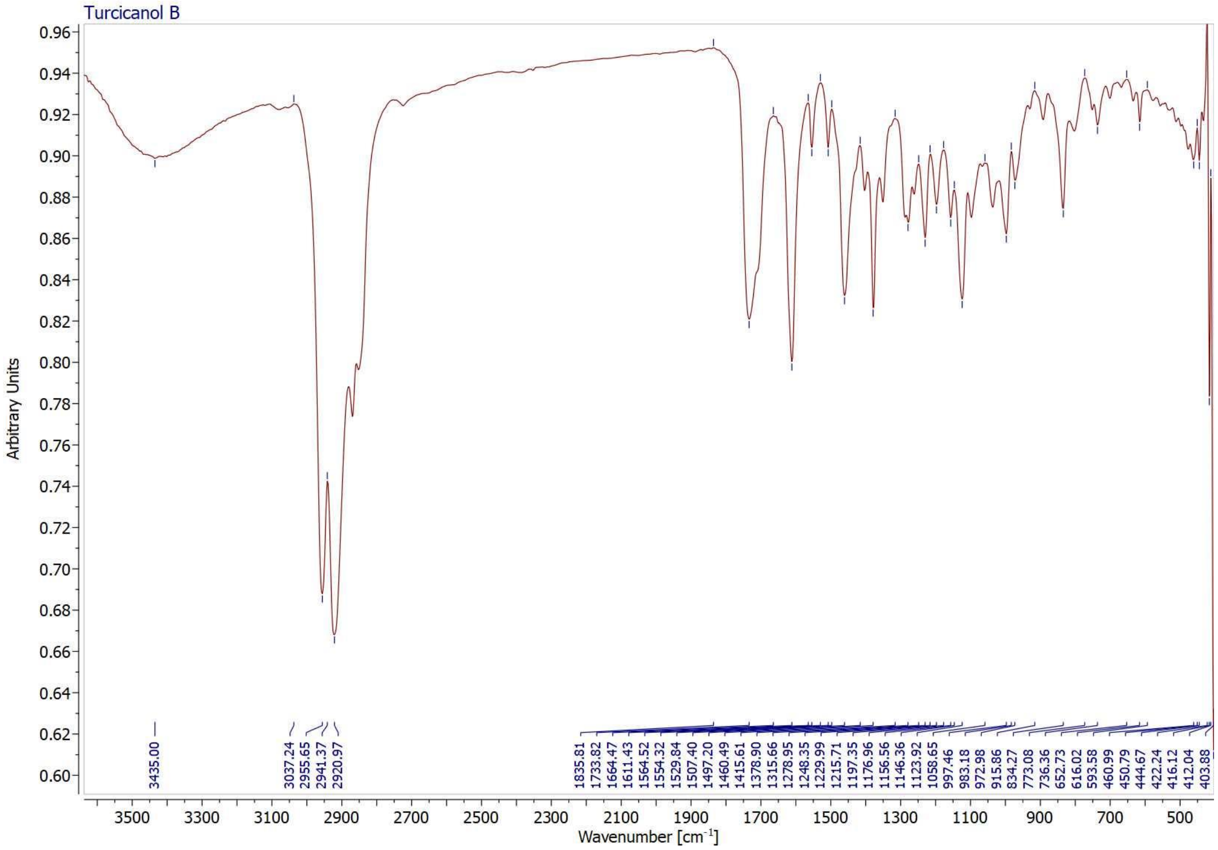

Figure S29 IR spectrum (NaCl) of turcicanol B (**3**)

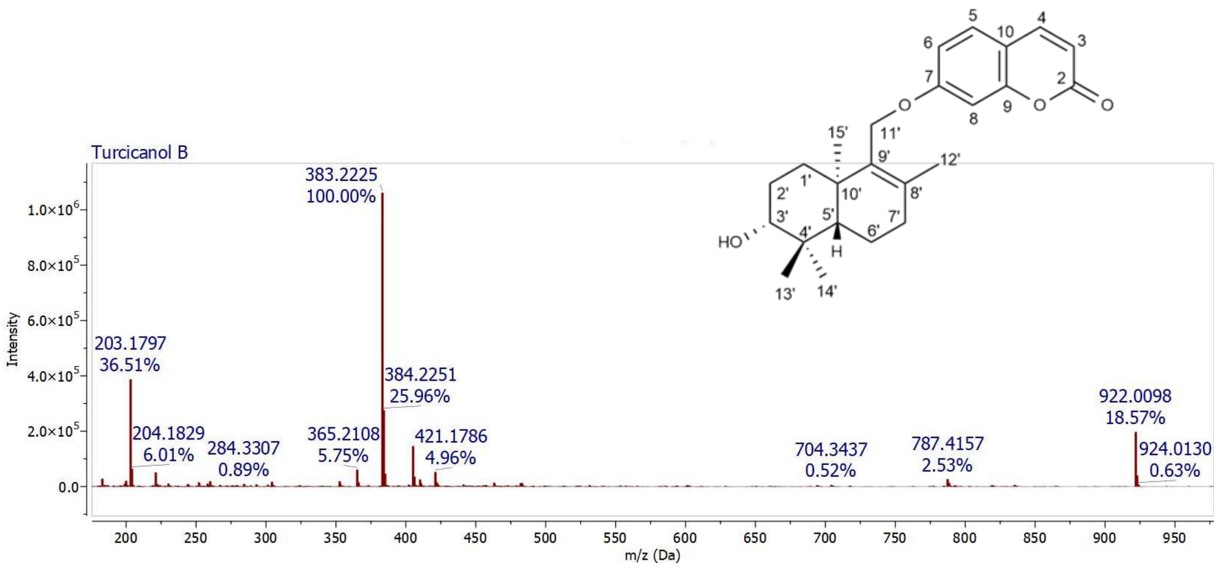

Figure S30 HRMS of turcicanol B (**3**)

m/z $[M+H]^+$ 383.2225 (calculated: 383.2222)

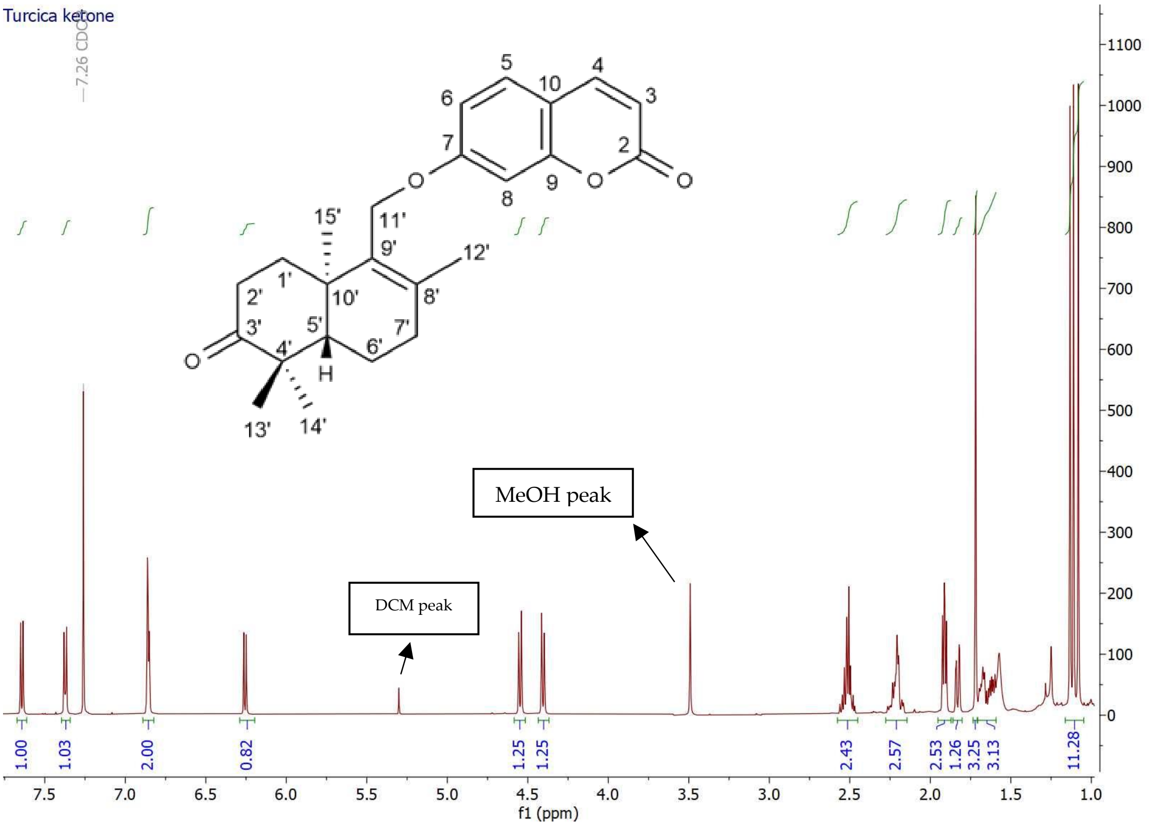

Figure S31 ^{1}H-NMR spectrum (600 MHz, $CDCl_3$) of turcica ketone (**4**)

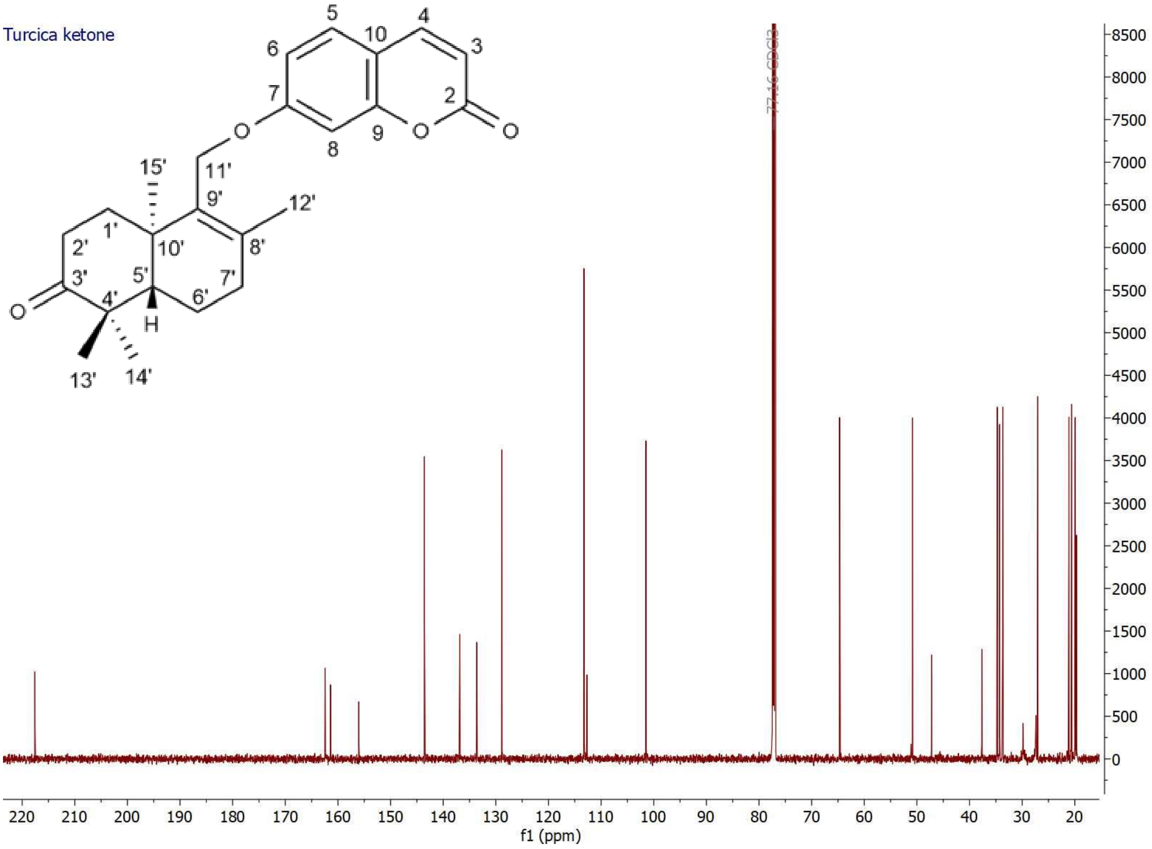

Figure S32 ^{13}C-NMR spectrum (150 MHz, $CDCl_3$) of turcica ketone (**4**)

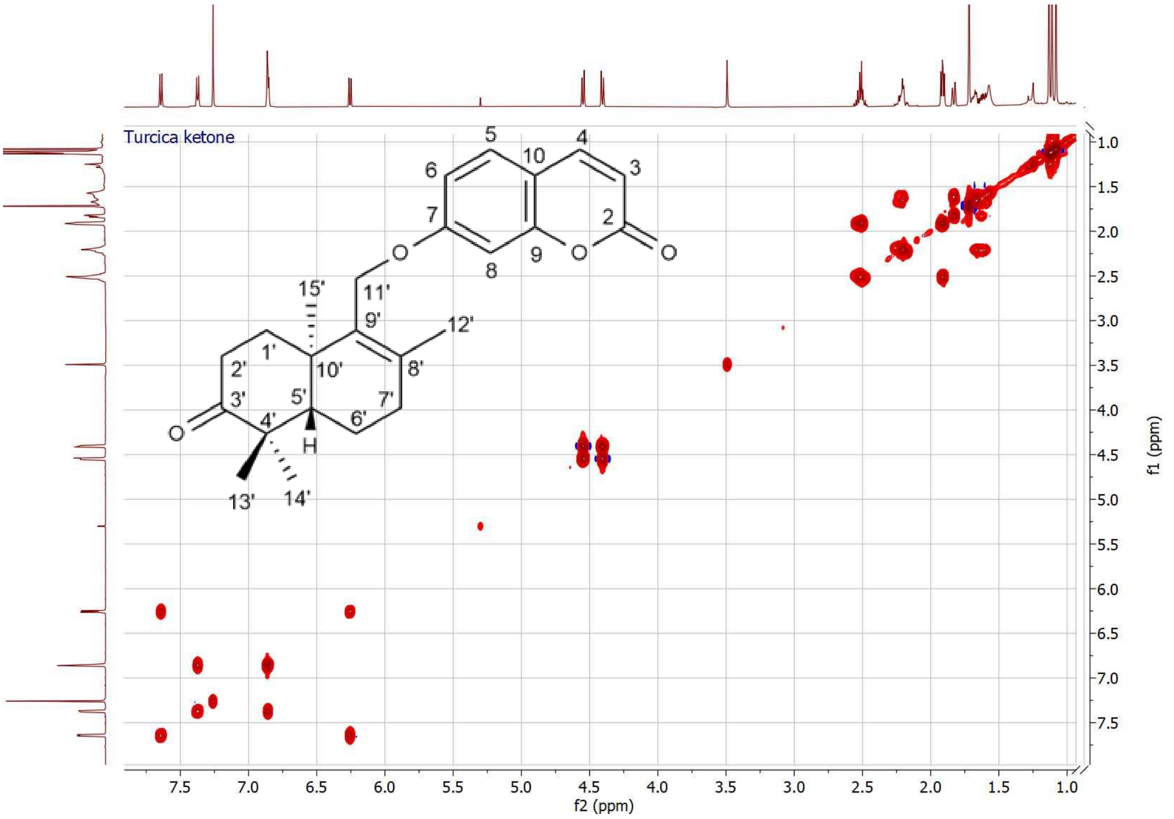

Figure S33 COSY spectrum of turcica ketone (**4**)

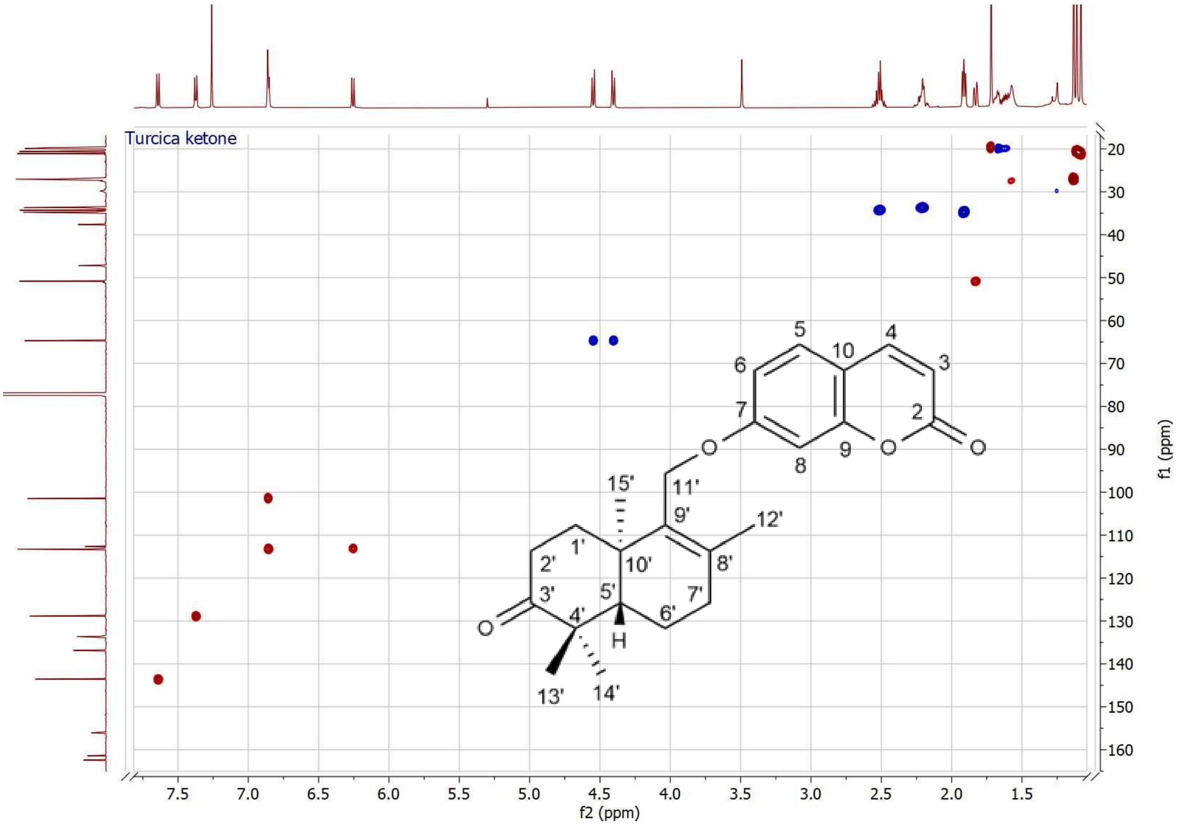

Figure S34 HSQC spectrum of turcica ketone (**4**)

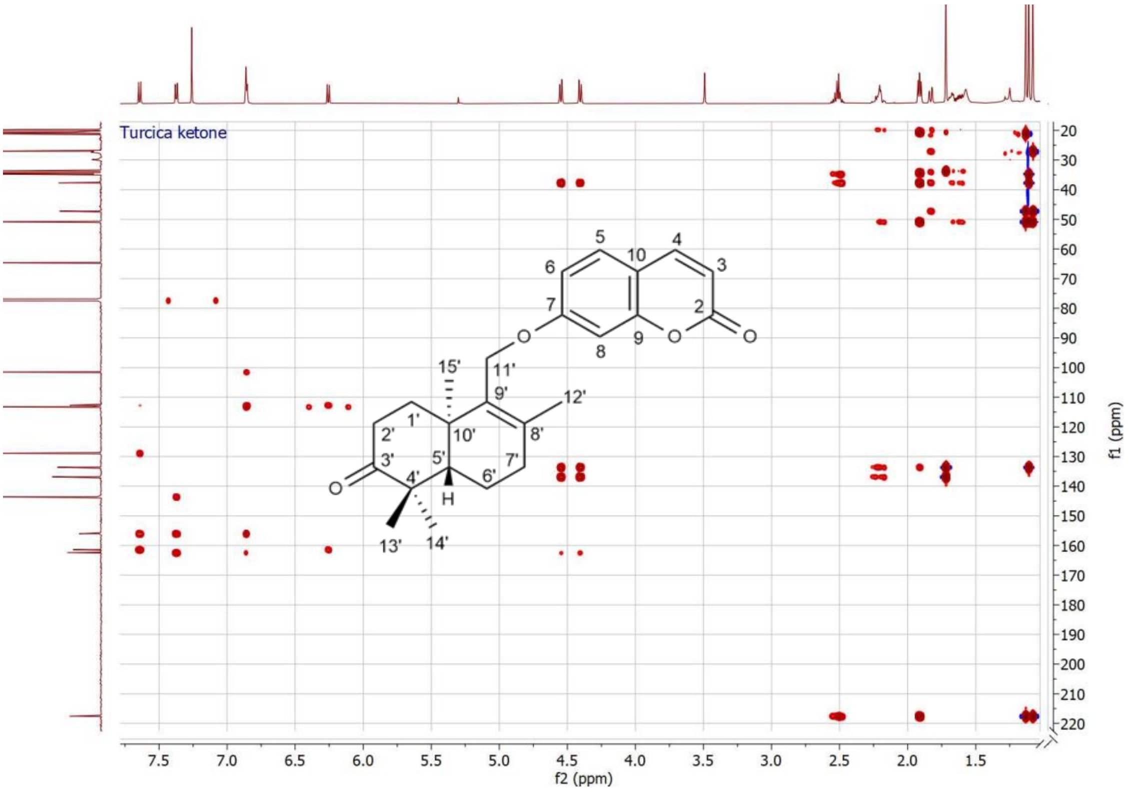

Figure S35 HMBC spectrum of turcica ketone (**4**)

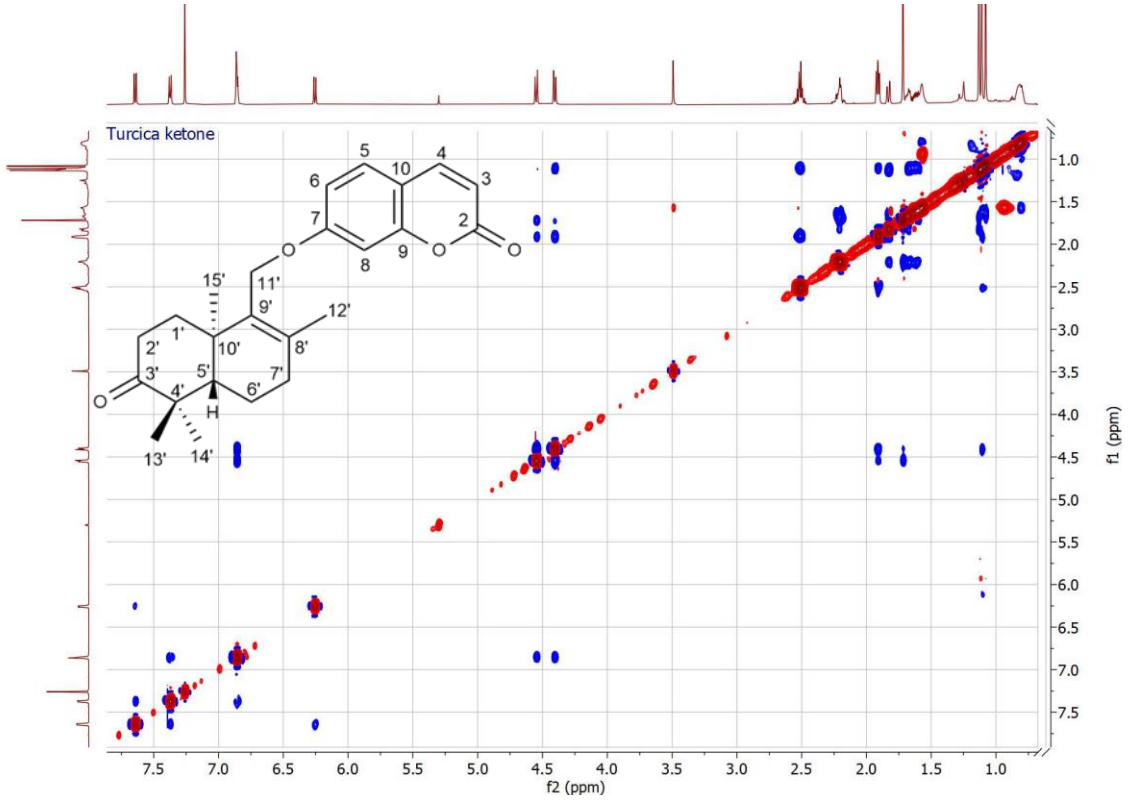

Figure S36 NOESY spectrum of turcica ketone (**4**)

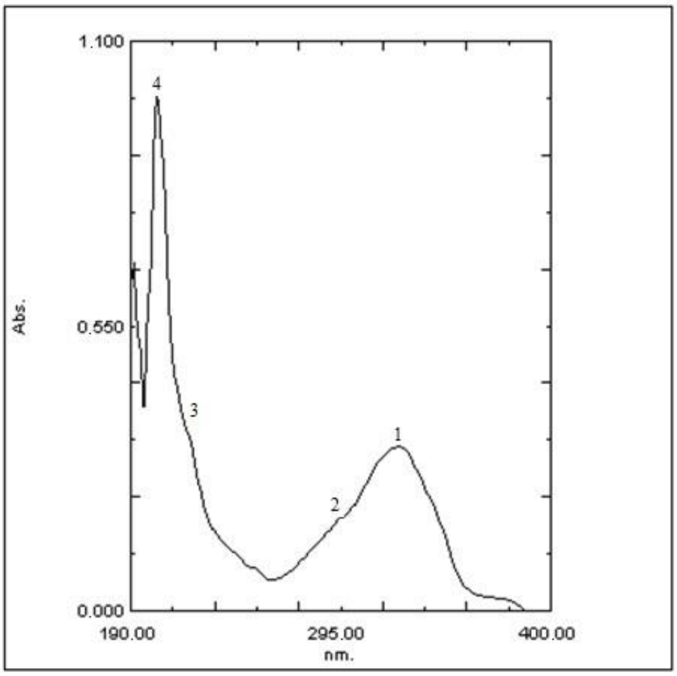

	Wavelength	Absorbance
1	324.00	0.317
2	295.00	0.179
3	217.00	0.364
4	203.00	0.994

Figure S37 UV spectrum (MeOH) of turcica ketone (**4**)

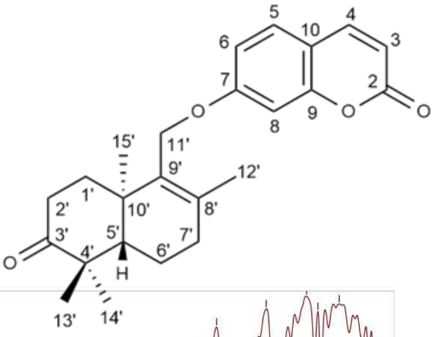

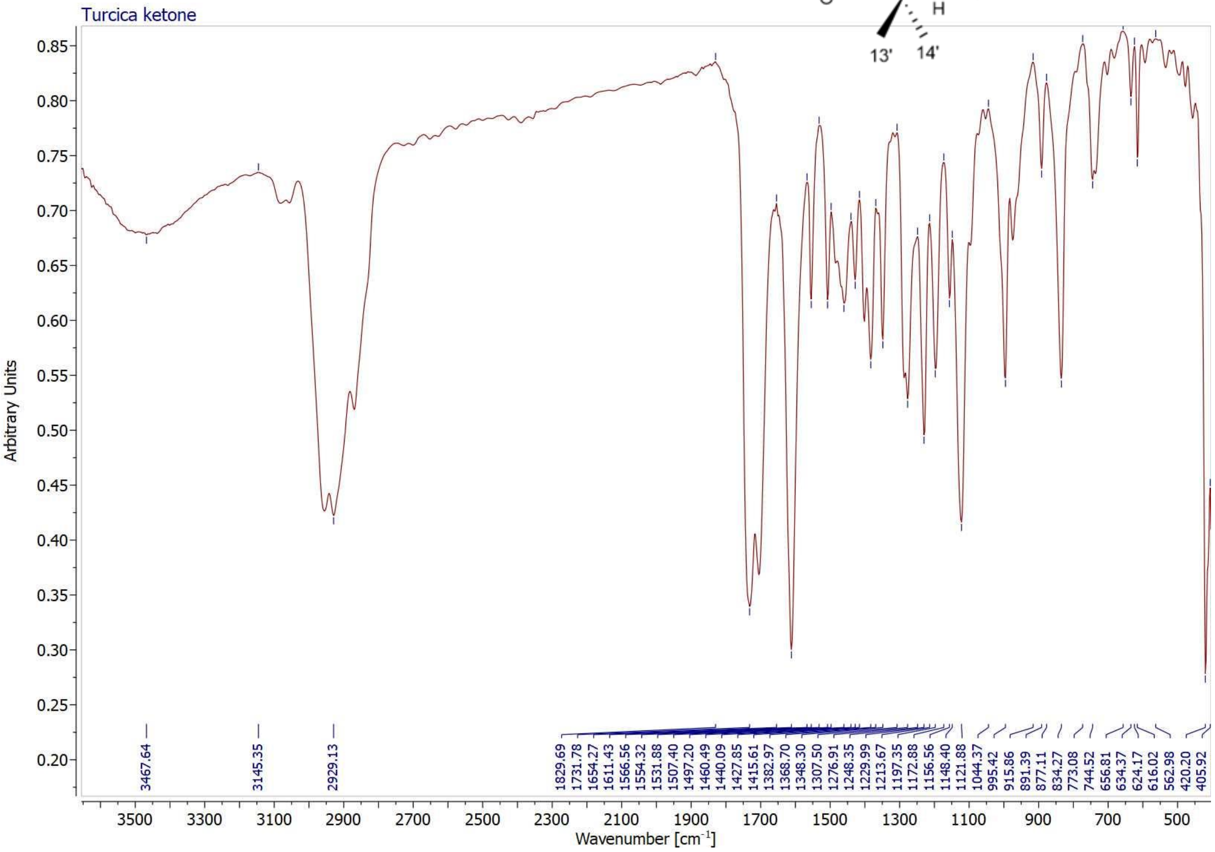

Figure S38 IR spectrum (NaCl) of turcica ketone (**4**)

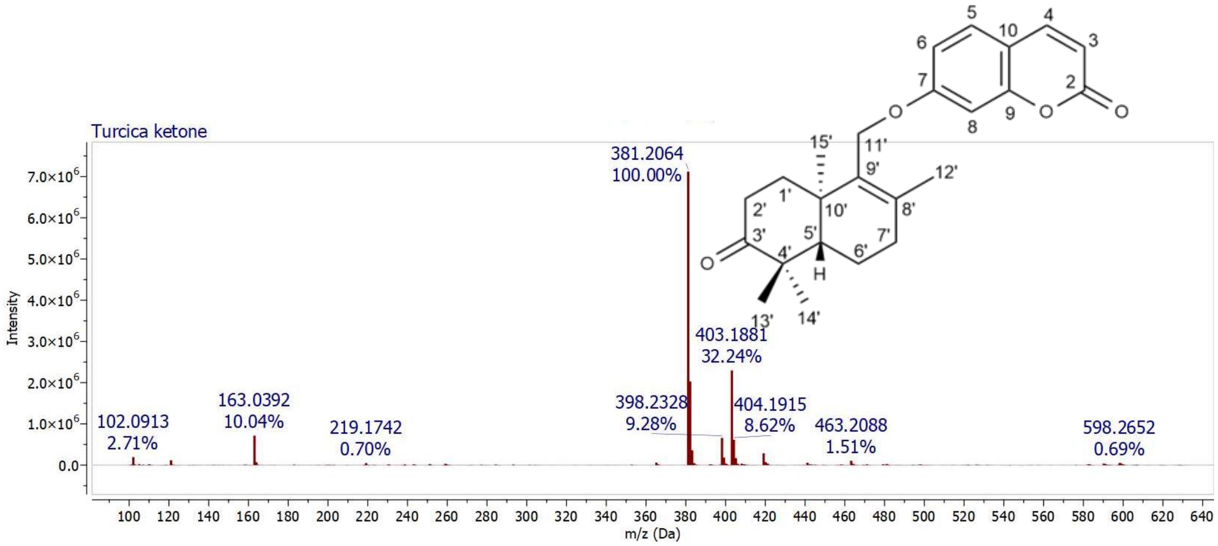

Figure S39 HRMS of turcica ketone (**4**)

m/z $[M+H]^+$ 381.2064 (calculated: 381.2066)

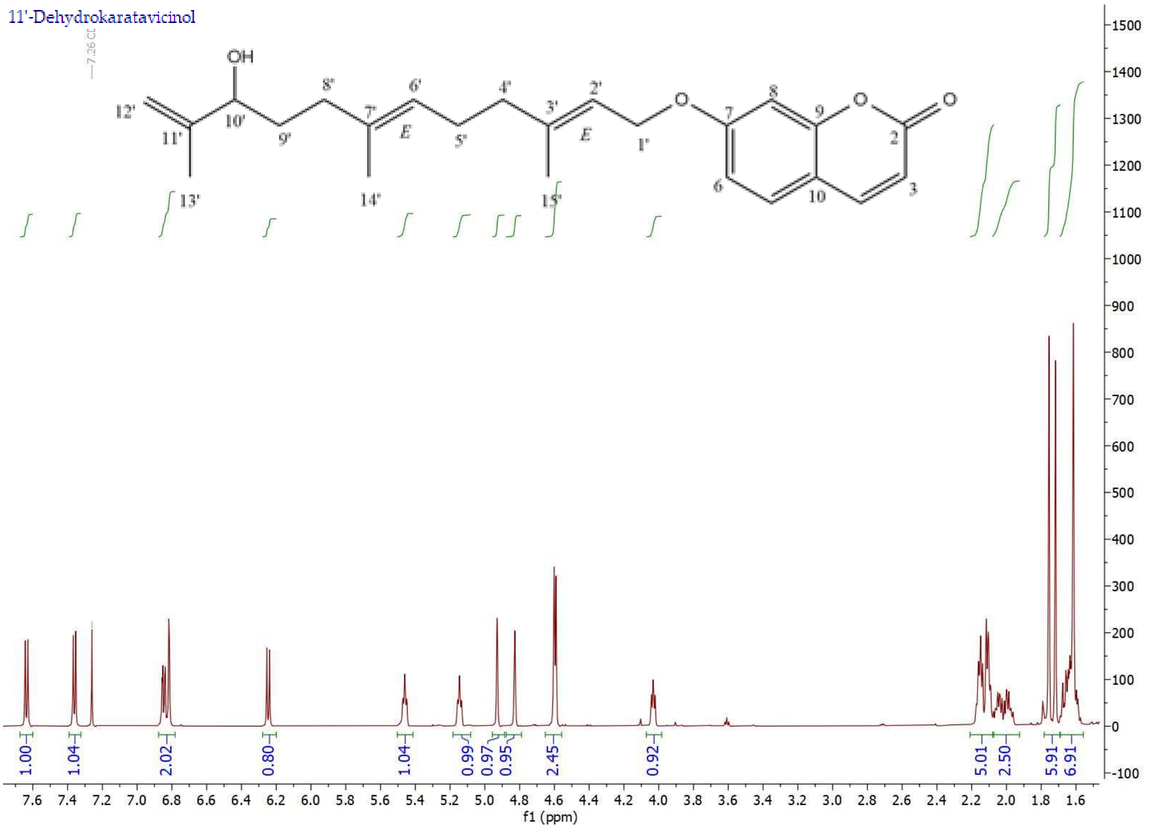

Figure S40 ^{1}H-NMR spectrum (600 MHz, $CDCl_3$) of 11'-dehydrokaratavicinol (**5**)

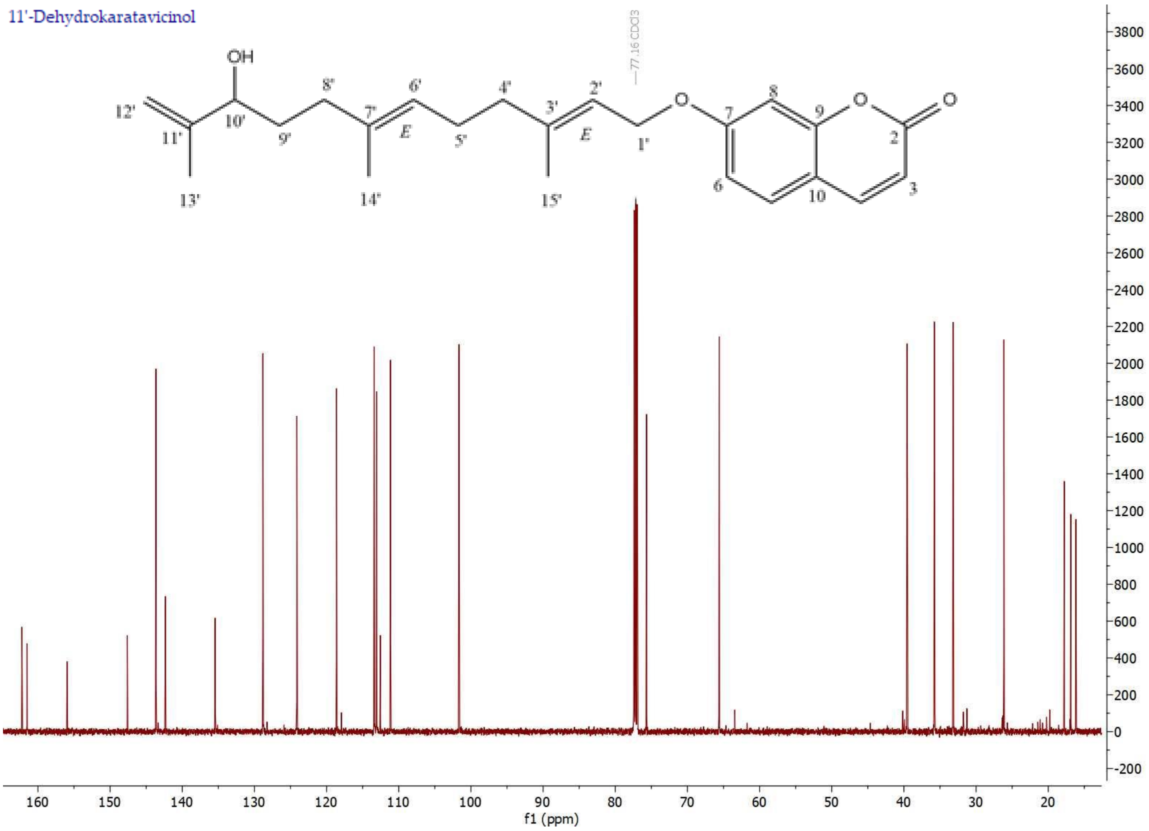

Figure S41 ^{13}C-NMR spectrum (150 MHz, $CDCl_3$) of 11'-dehydrokaratavicinol (**5**)

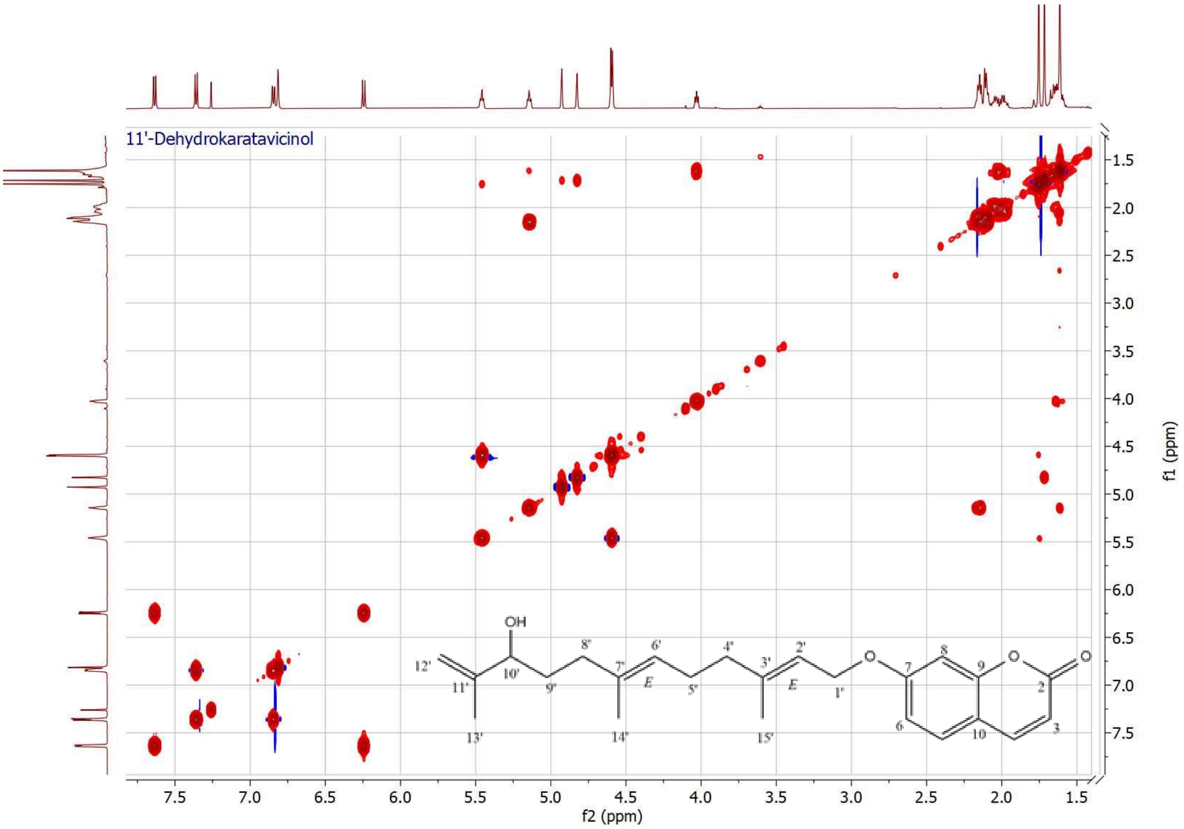

Figure S42 COSY spectrum of 11'-dehydrokaratavicinol (**5**)

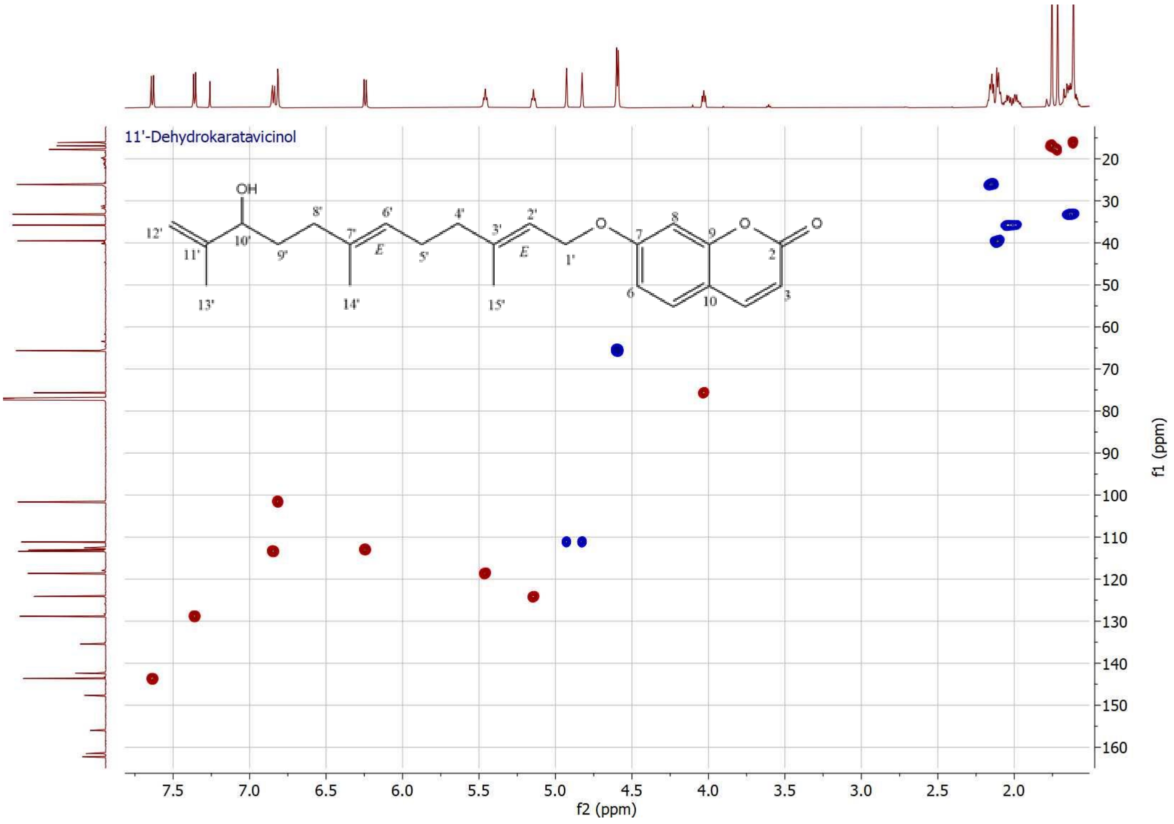

Figure S43 HSQC spectrum of 11'-dehydrokaratavicinol (**5**)

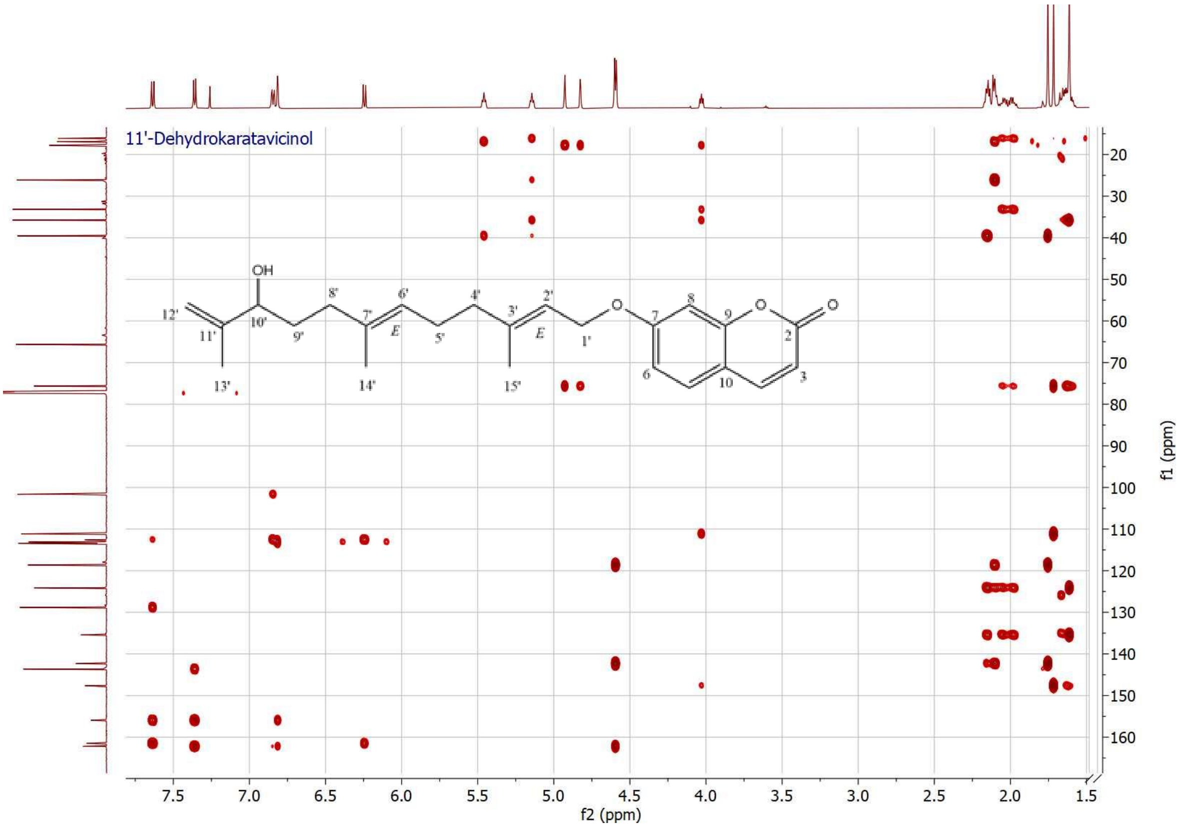

Figure S44 HMBC spectrum of 11'-dehydrokaratavicinol (**5**)

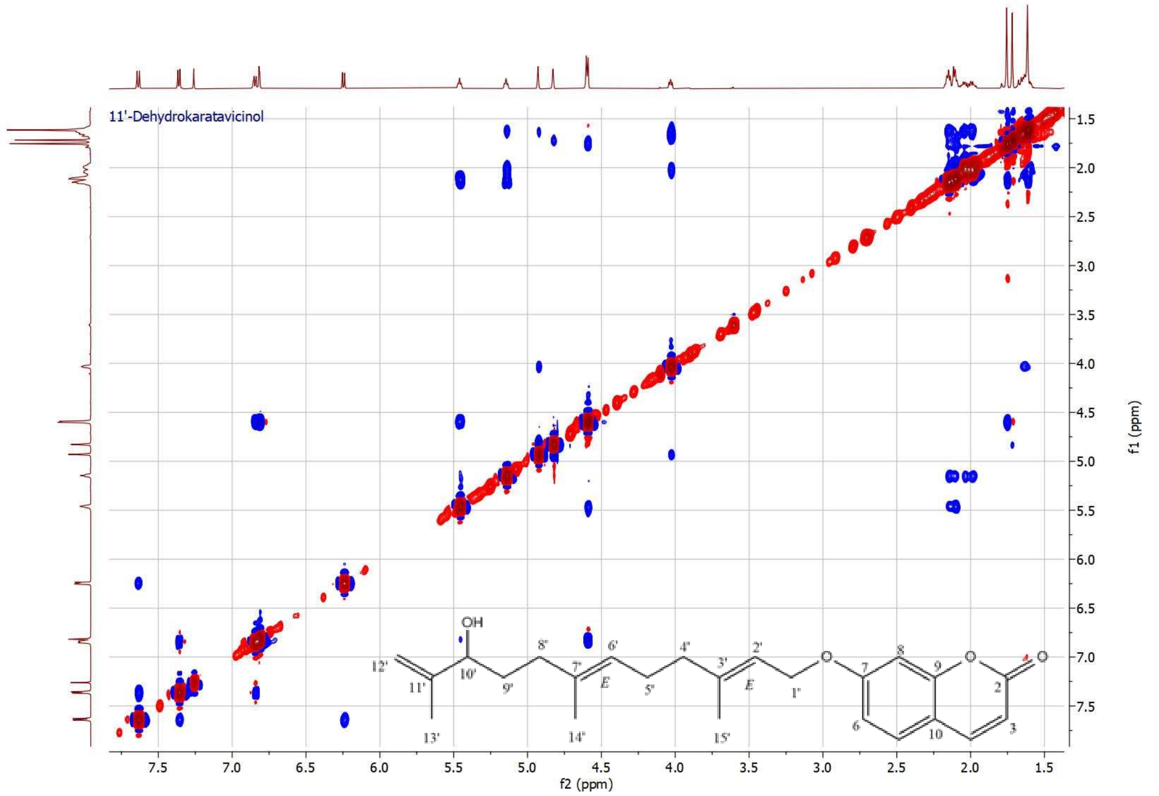

Figure S45 NOESY spectrum of 11'-dehydrokaratavicinol (**5**)

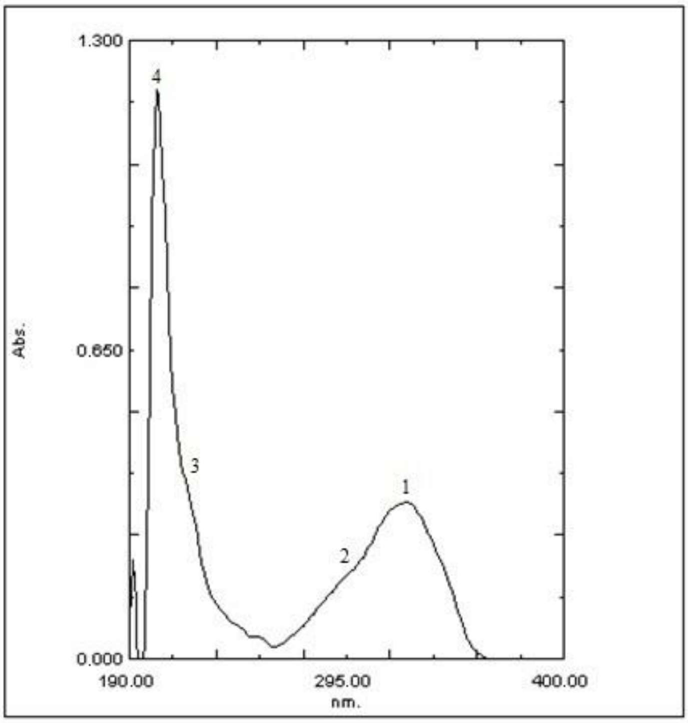

	Wavelength	Absorbance
1	324.00	0.329
2	299.00	0.191
3	215.00	0.415
4	203.00	1.195

Figure S46 UV spectrum (MeOH) of 11'-dehydrokaratavicinol (**5**)

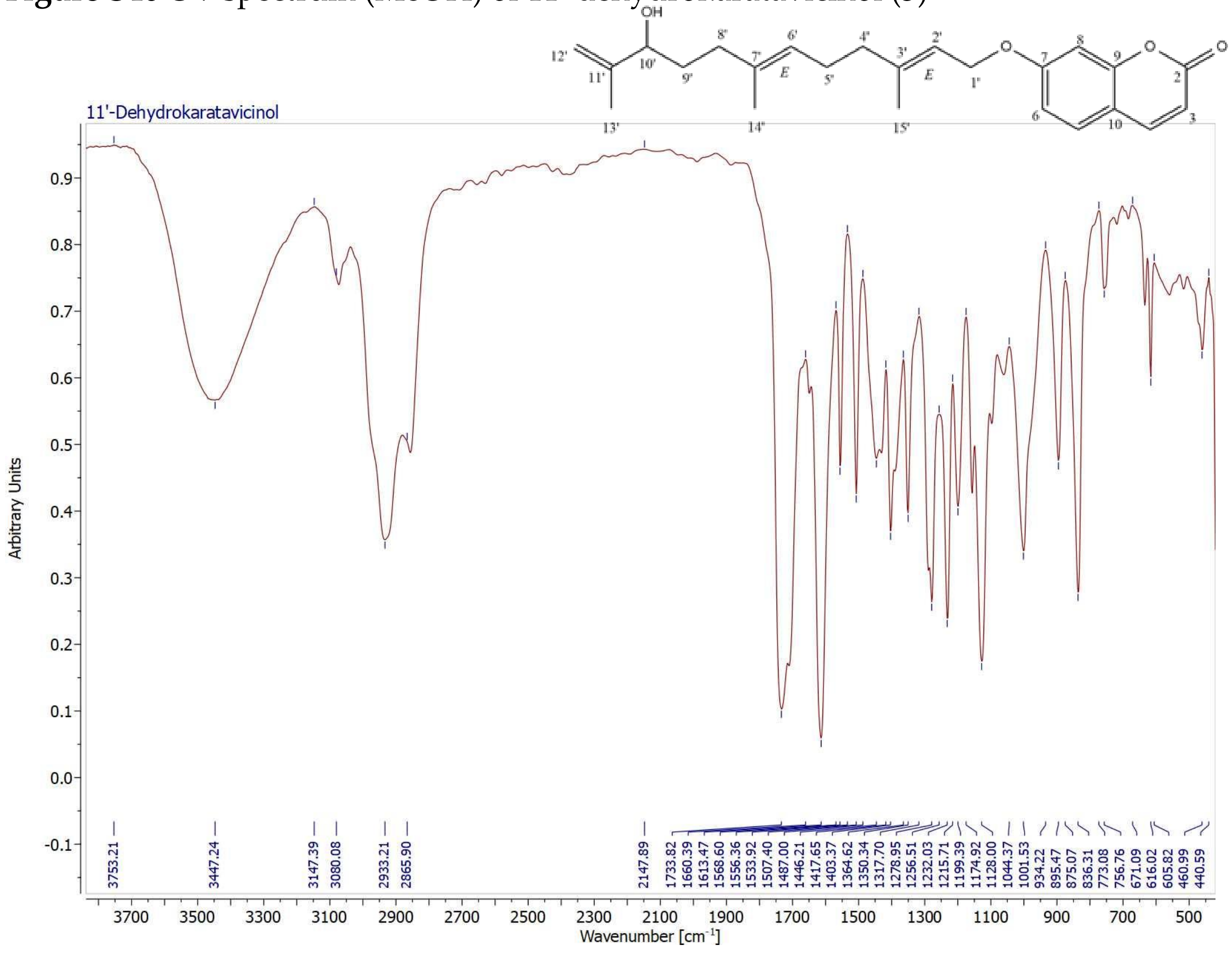

Figure S47 IR spectrum (NaCl) of 11'-dehydrokaratavicinol (**5**)

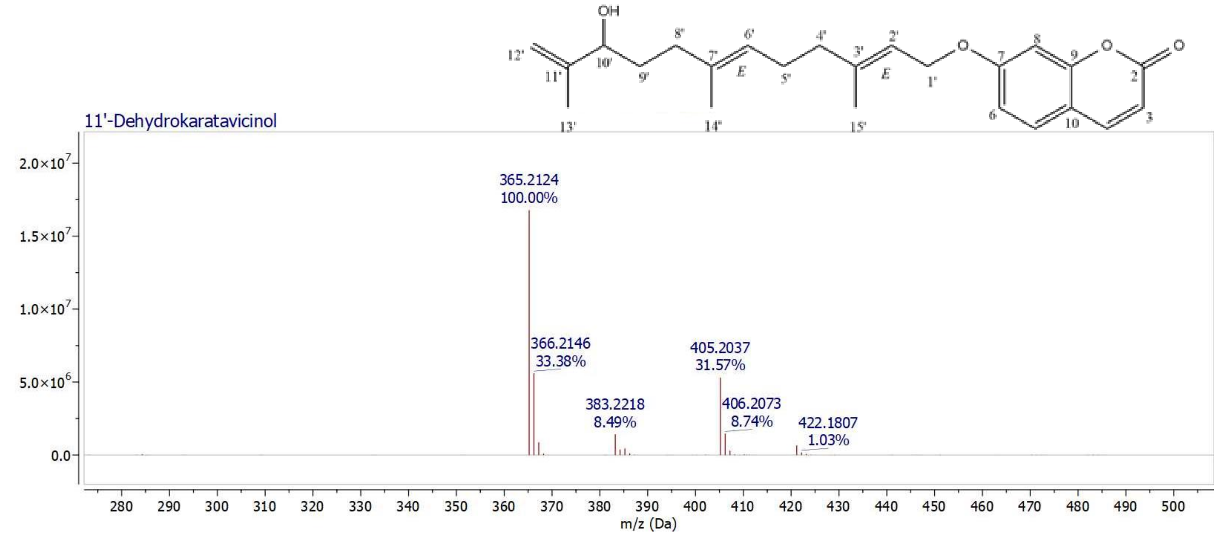

Figure S48 HRMS of 11'-dehydrokaratavicinol (**5**)

m/z $[M+Na]^+$ 405.2037 (calculated: 405.2042)

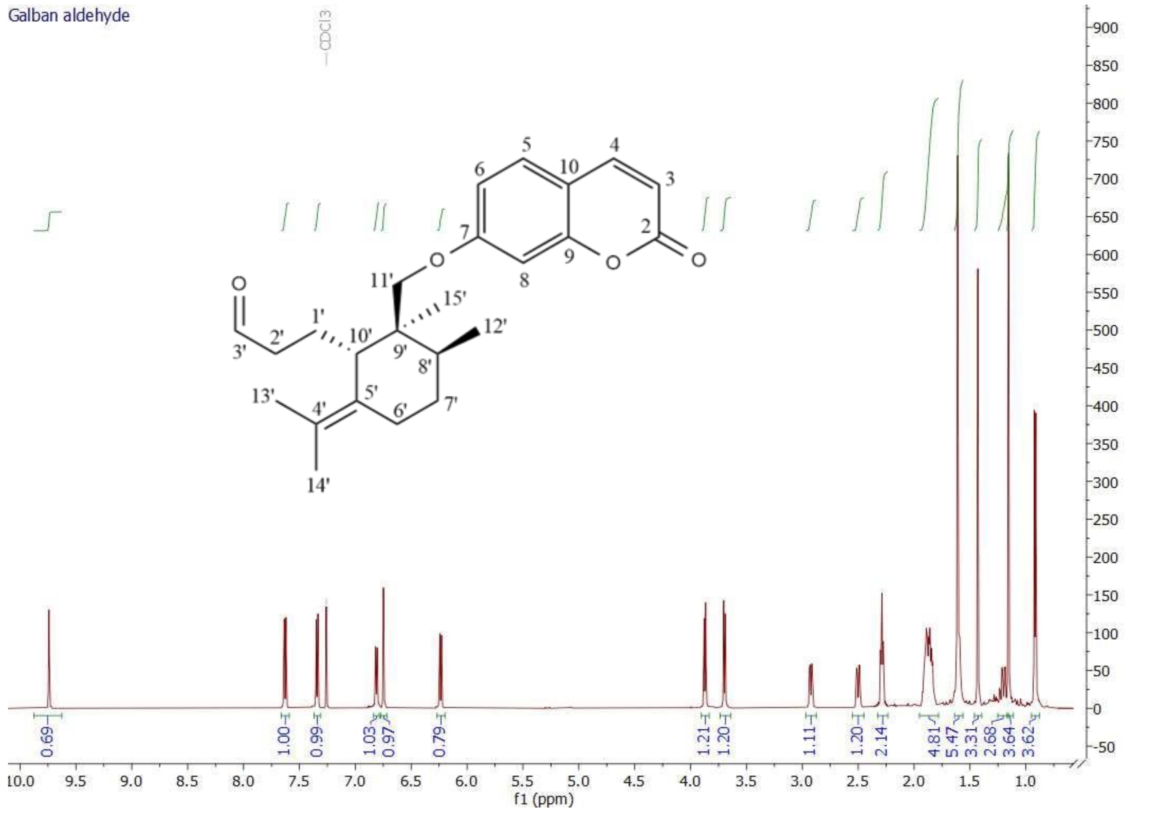

Figure S49 ^{1}H-NMR spectrum (600 MHz, $CDCl_3$) of galbanaldehyde (**6**)

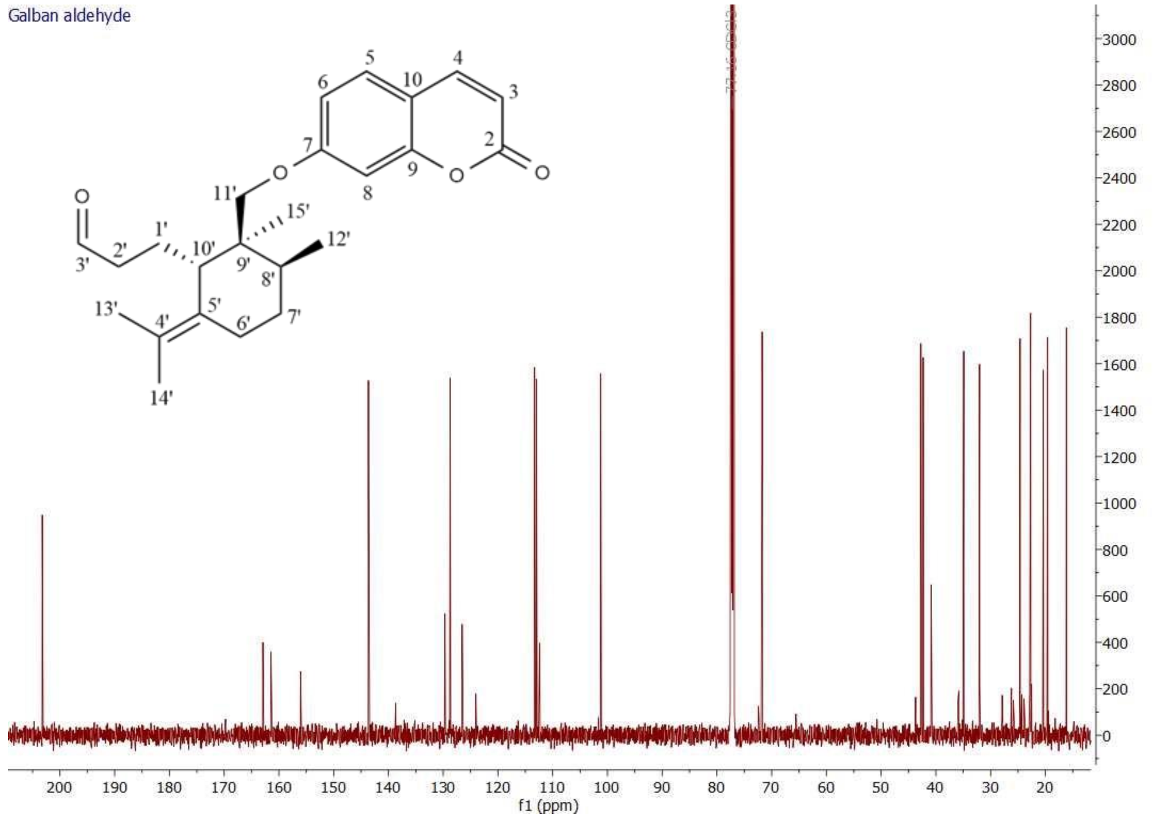

Figure S50 ^{13}C-NMR spectrum (150 MHz, $CDCl_3$) of galbanaldehyde (**6**)

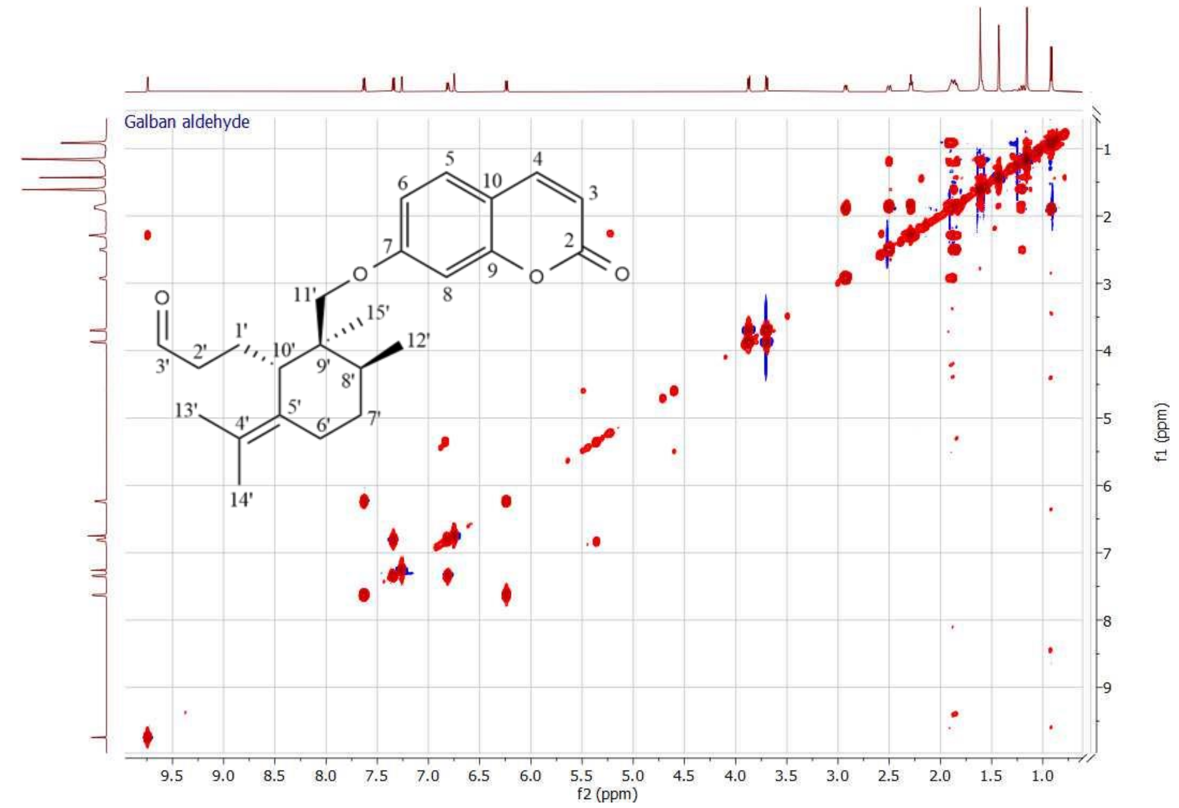

Figure S51 COSY spectrum of galbanaldehyde (**6**)

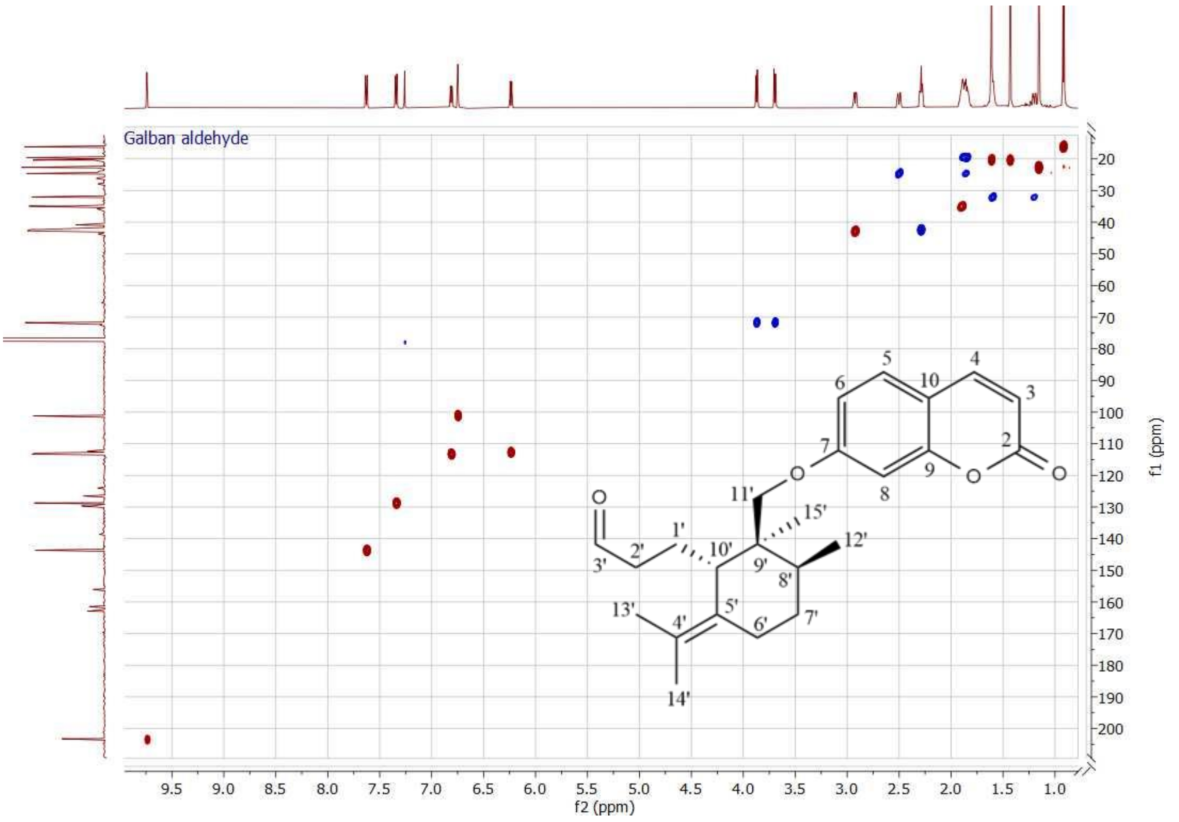

Figure S52 HSQC spectrum of galbanaldehyde (**6**)

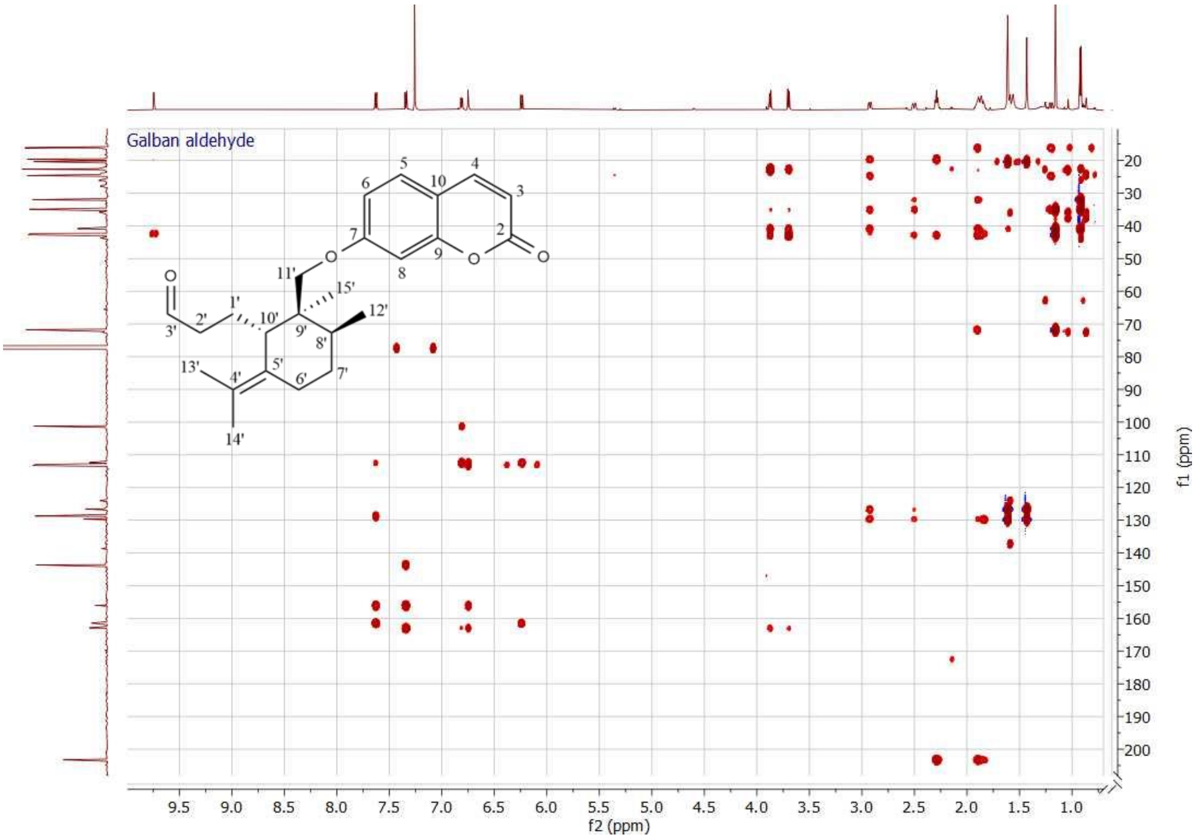

Figure S53 HMBC spectrum of galbanaldehyde (**6**)

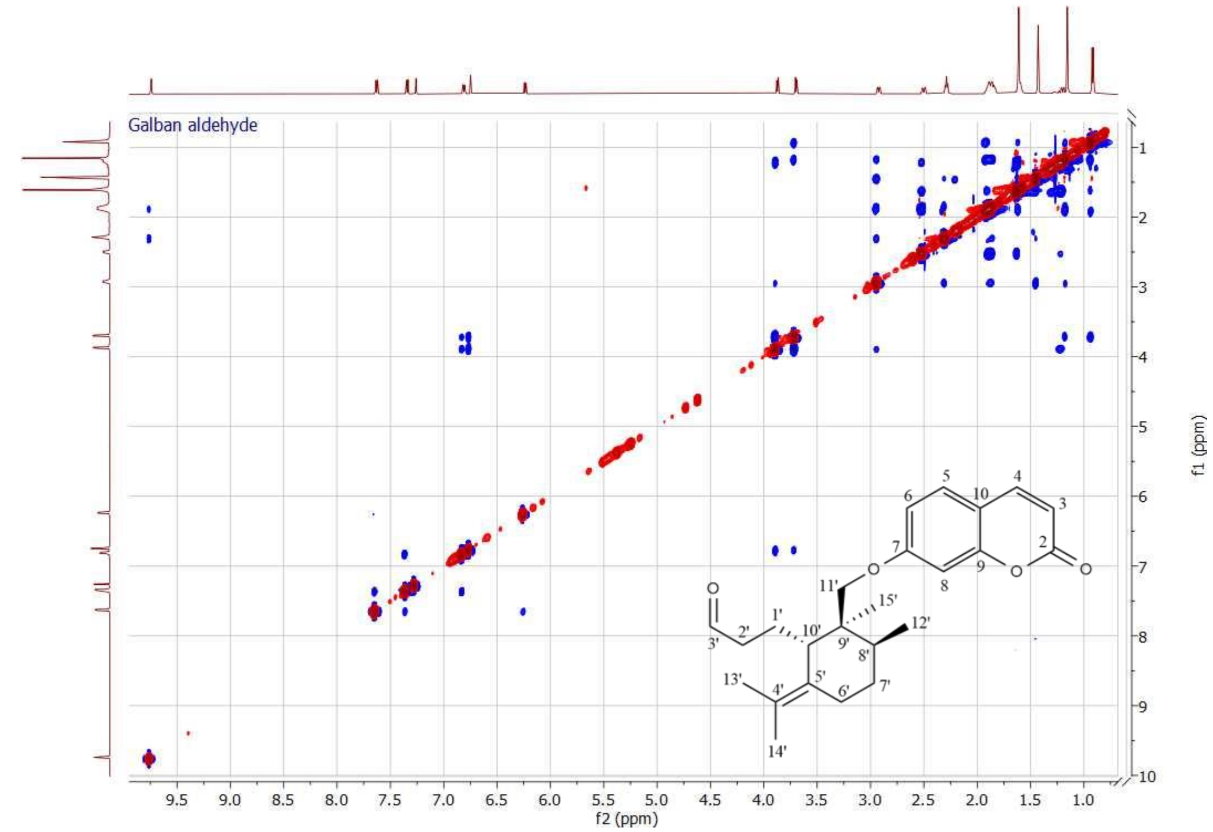

Figure S54 NOESY spectrum of galbanaldehyde (**6**)

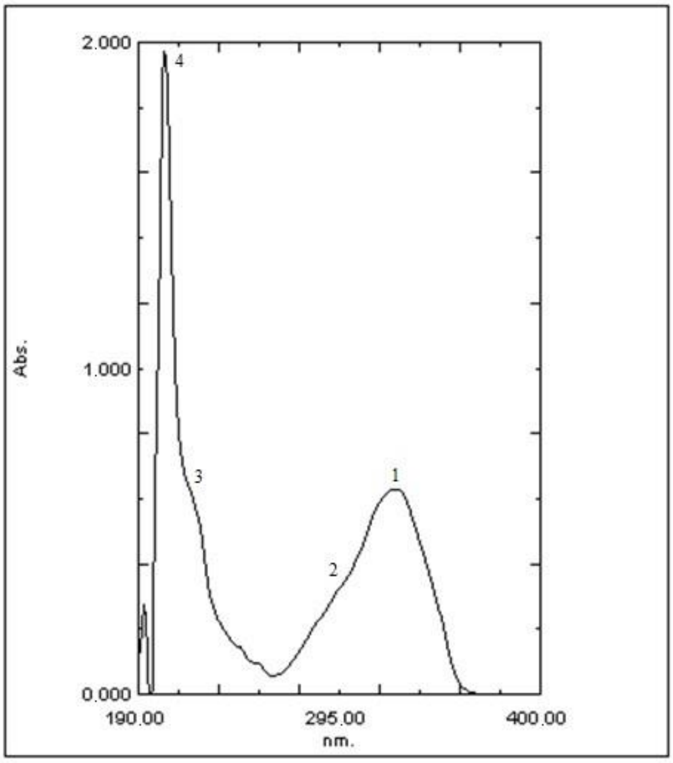

	Wavelength	Absorbance
1	324.00	0.629
2	295.00	0.325
3	218.00	0.615
4	203.00	1.974

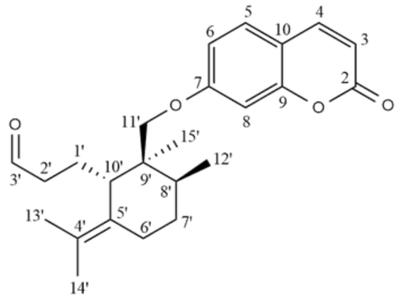

Figure S55 UV spectrum (MeOH) of galbanaldehyde (**6**)

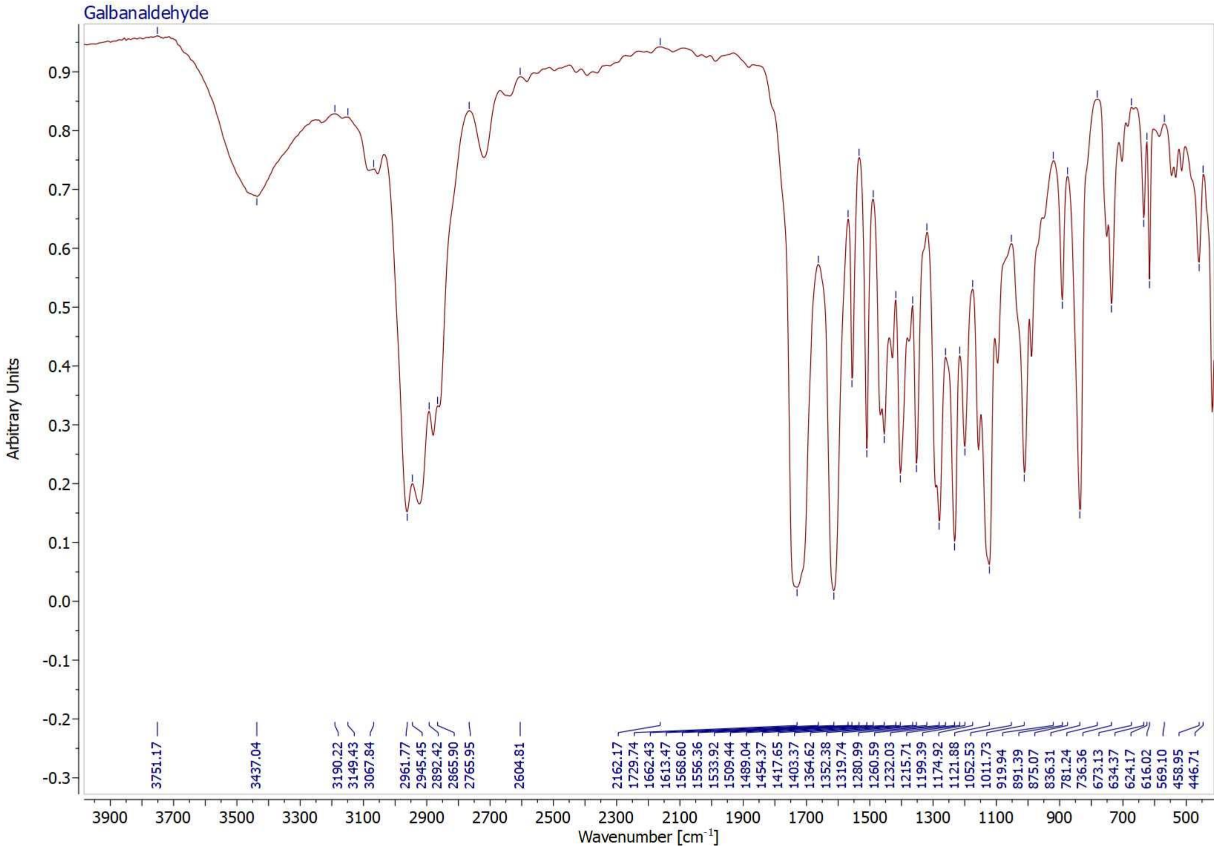

Figure S56 IR spectrum (NaCl) of galbanaldehyde (**6**)

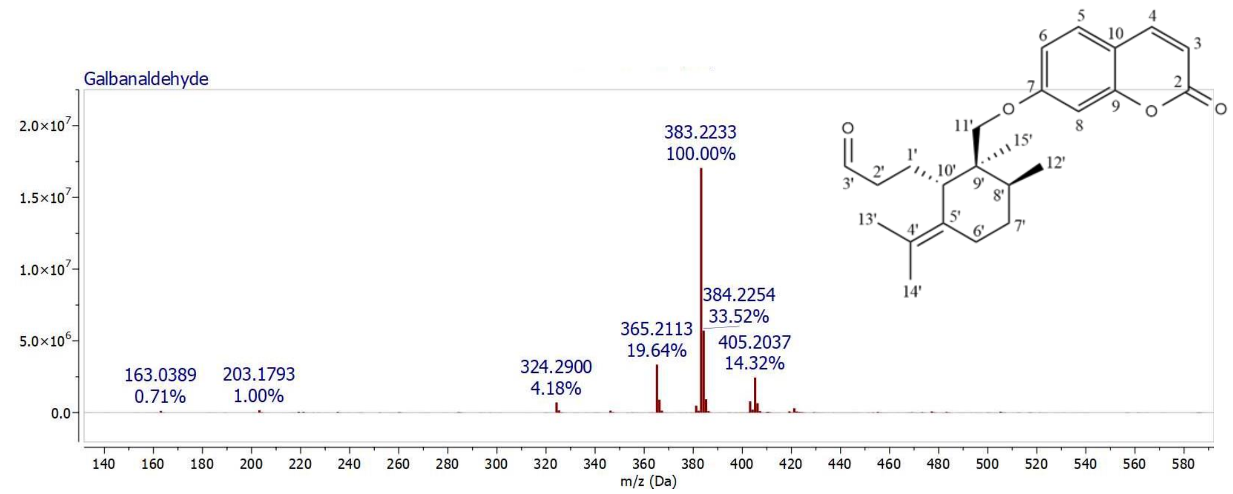

Figure S57 HRMS of galbanaldehyde (**6**)

m/z $[M+H]^+$ 383.2233 (calculated: 383.2222)

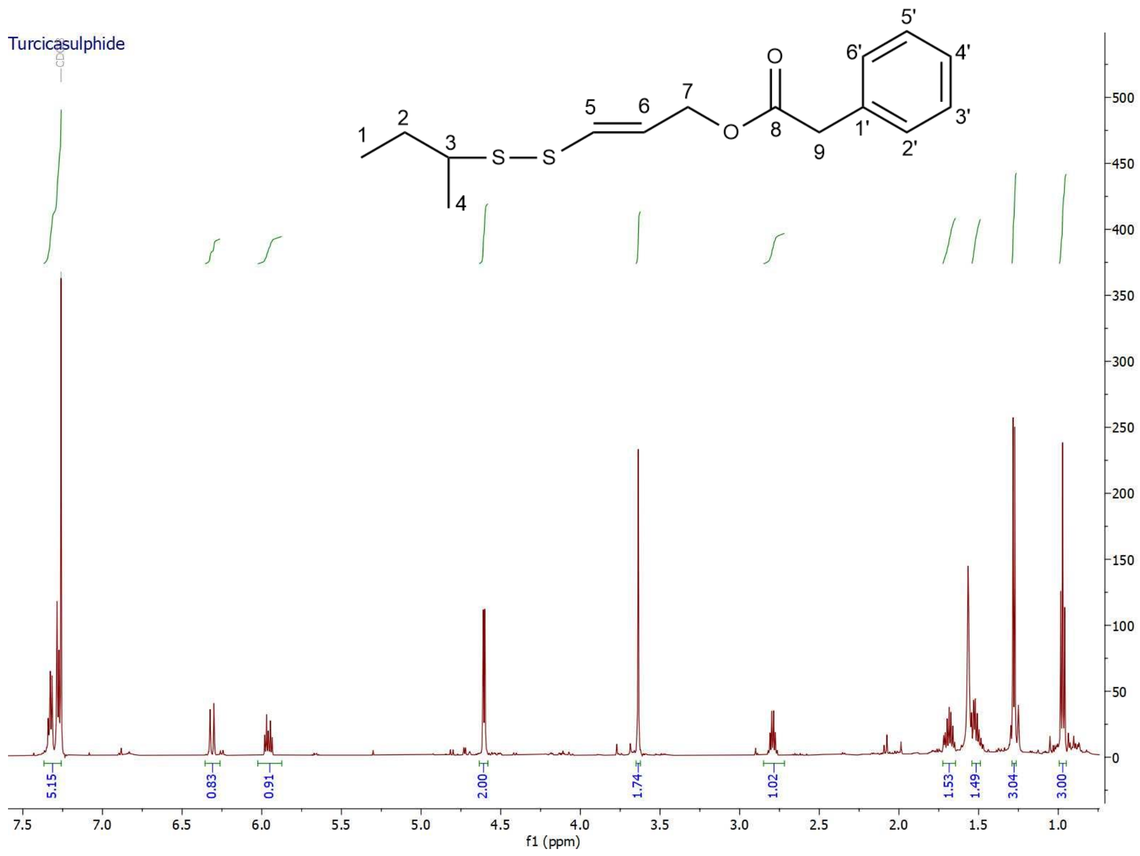

Figure S58 ^{1}H-NMR spectrum (600 MHz, $CDCl_3$) of turcicasulphide (**7**)

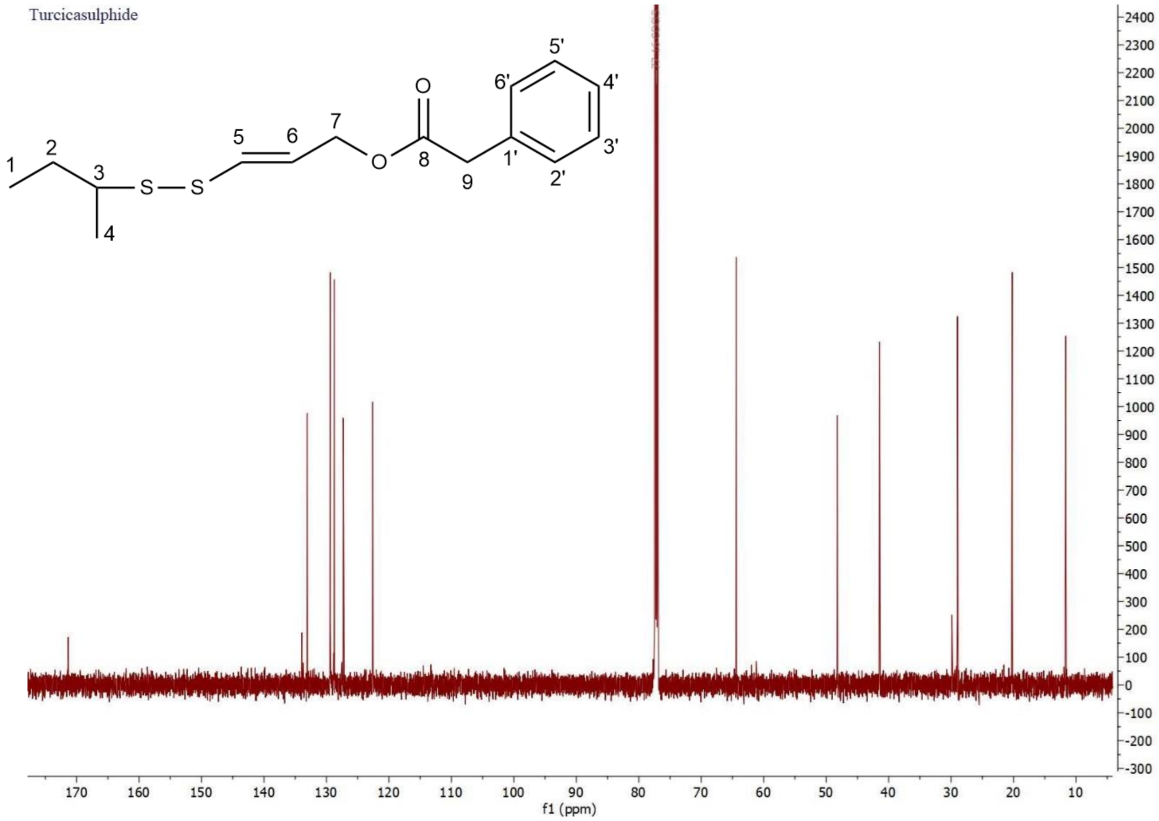

Figure S59 ^{13}C-NMR spectrum (150 MHz, $CDCl_3$) of turcicasulphide (**7**)

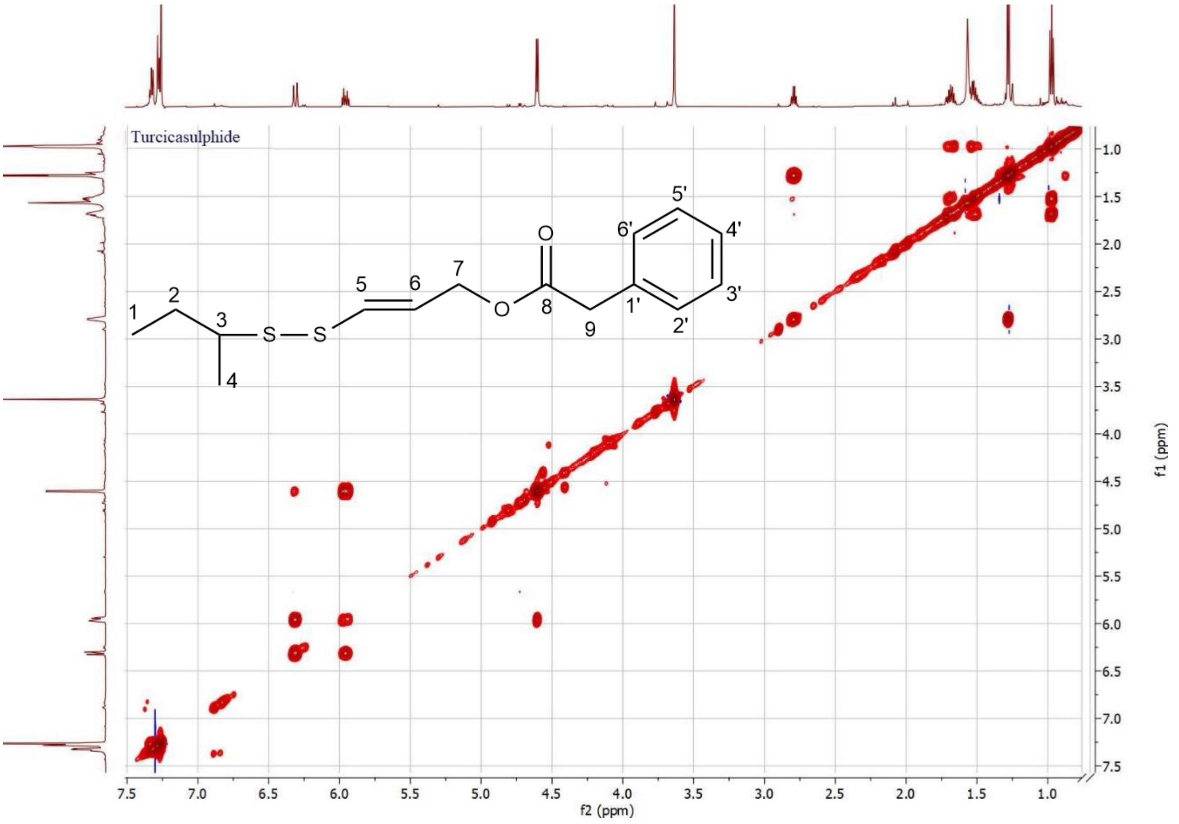

Figure S60 COSY spectrum of turcicasulphide (**7**)

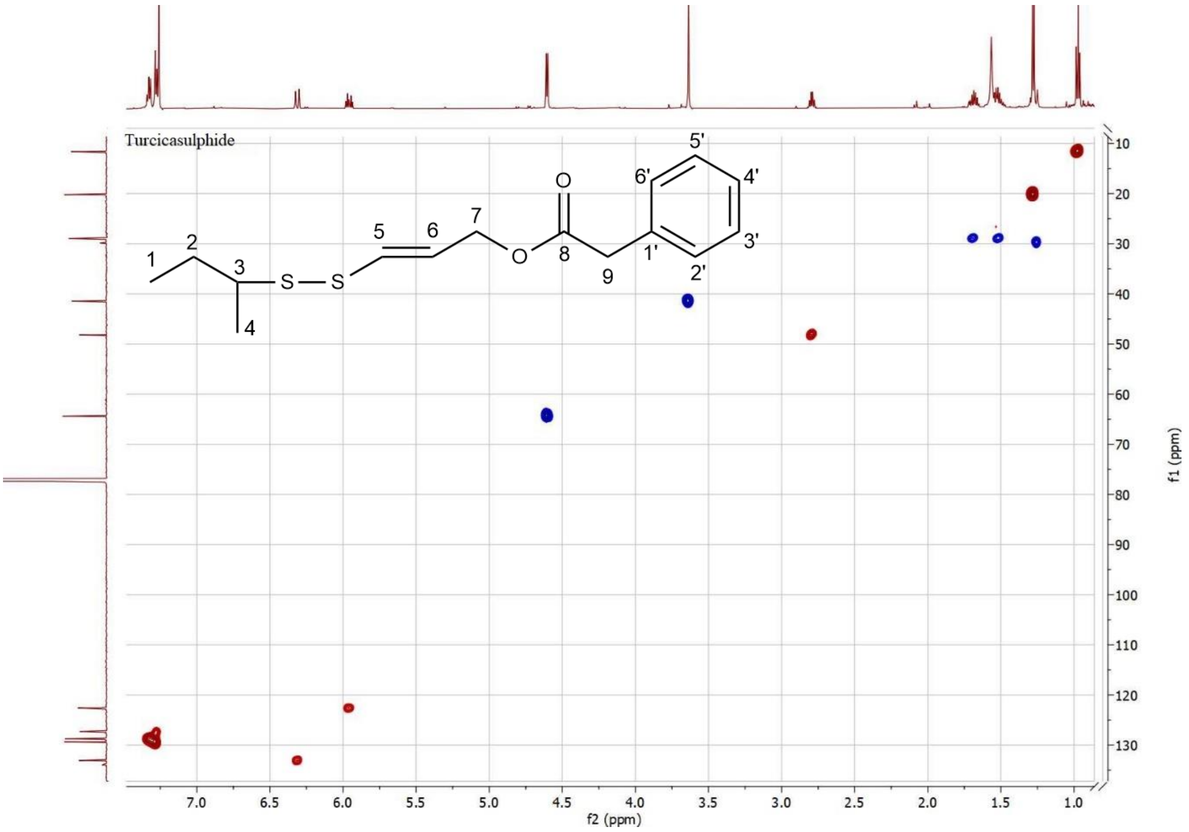

Figure S61 HSQC spectrum of turcicasulfide (**7**)

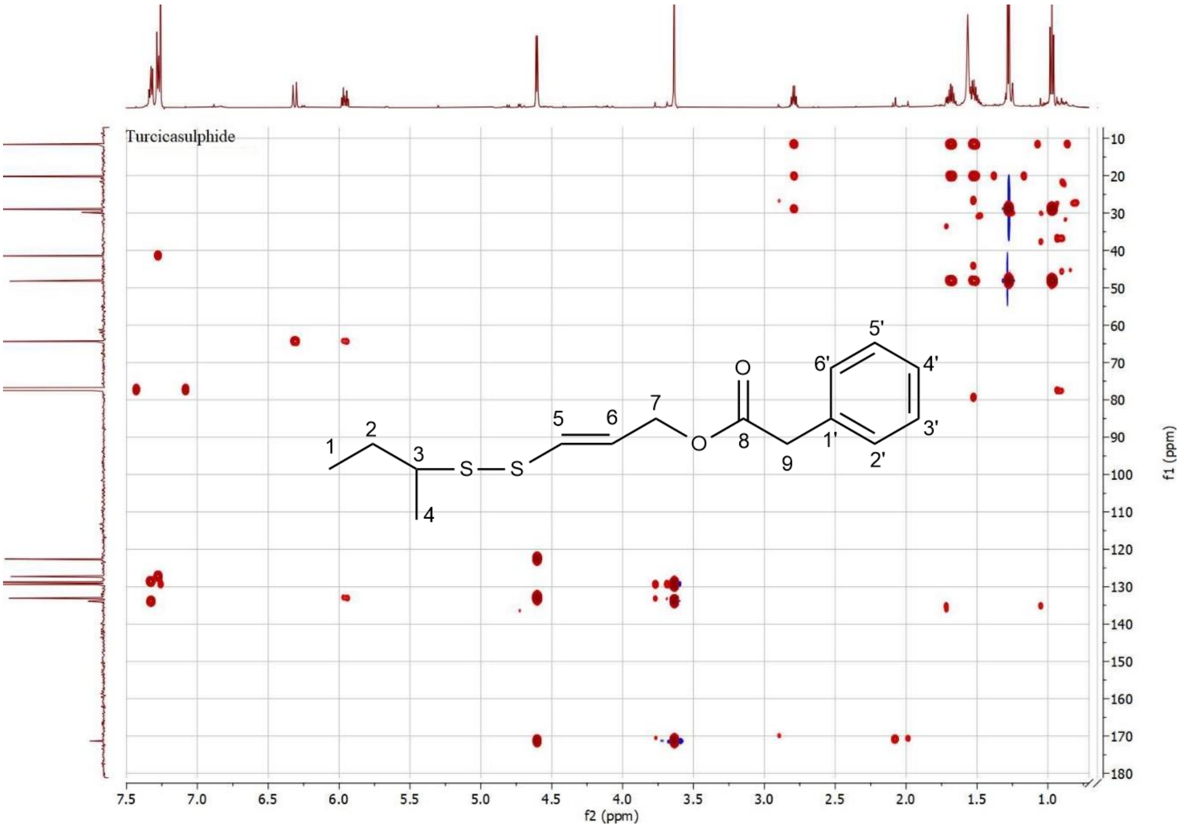

Figure S62 HMBC spectrum of turcicasulphide (**7**)

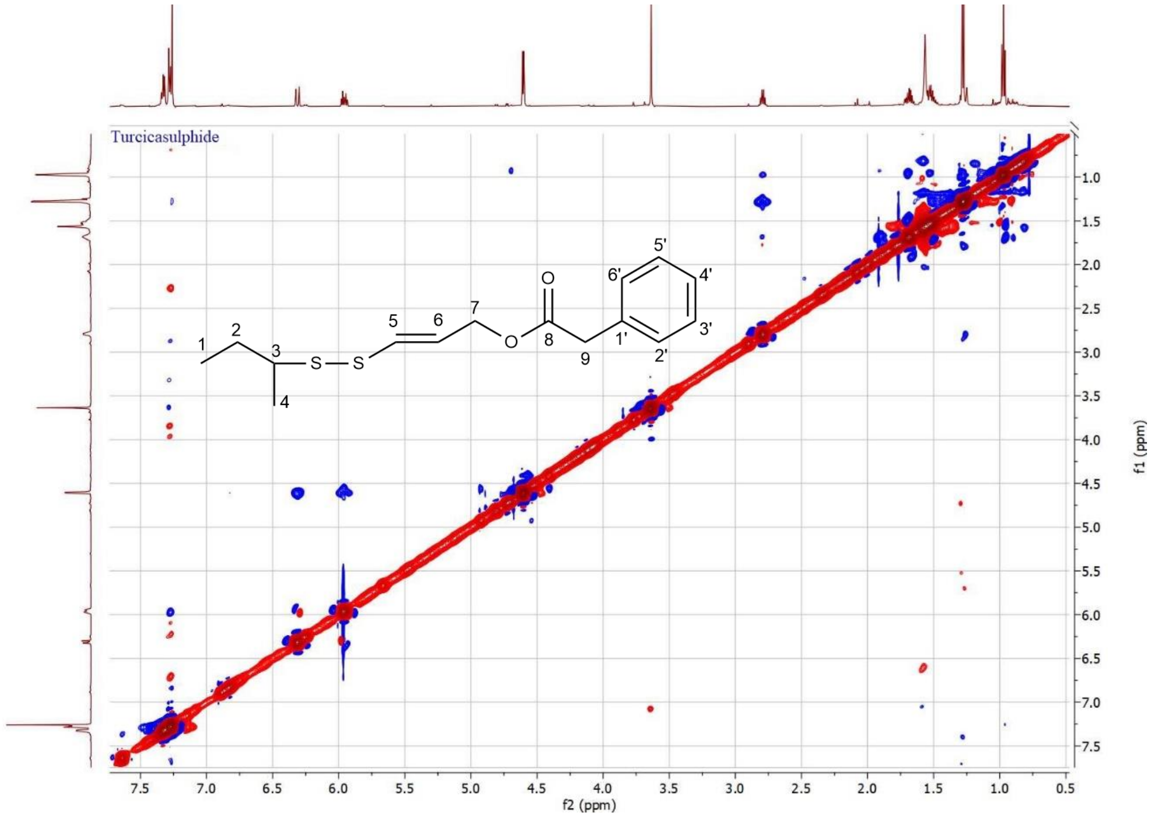

Figure S63 NOESY spectrum of turcicasulphide (**7**)

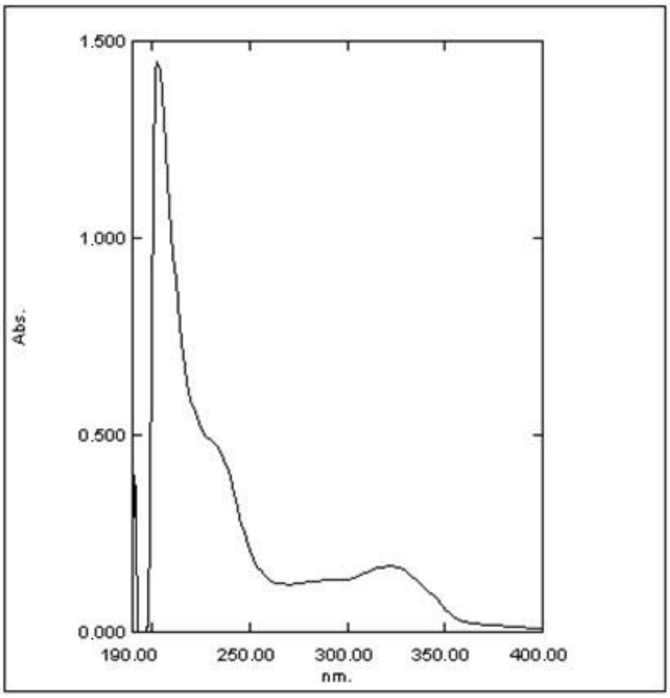

	Wavelength	Absorbance
1	322.00	0.167
2	234.00	0.469
3	203.00	1.445

Figure S64 UV spectrum (MeOH) of turcicasulphide (**7**)

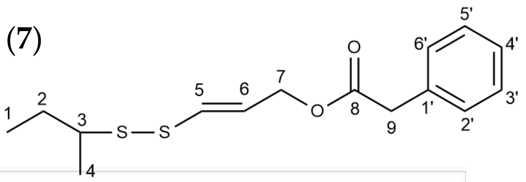

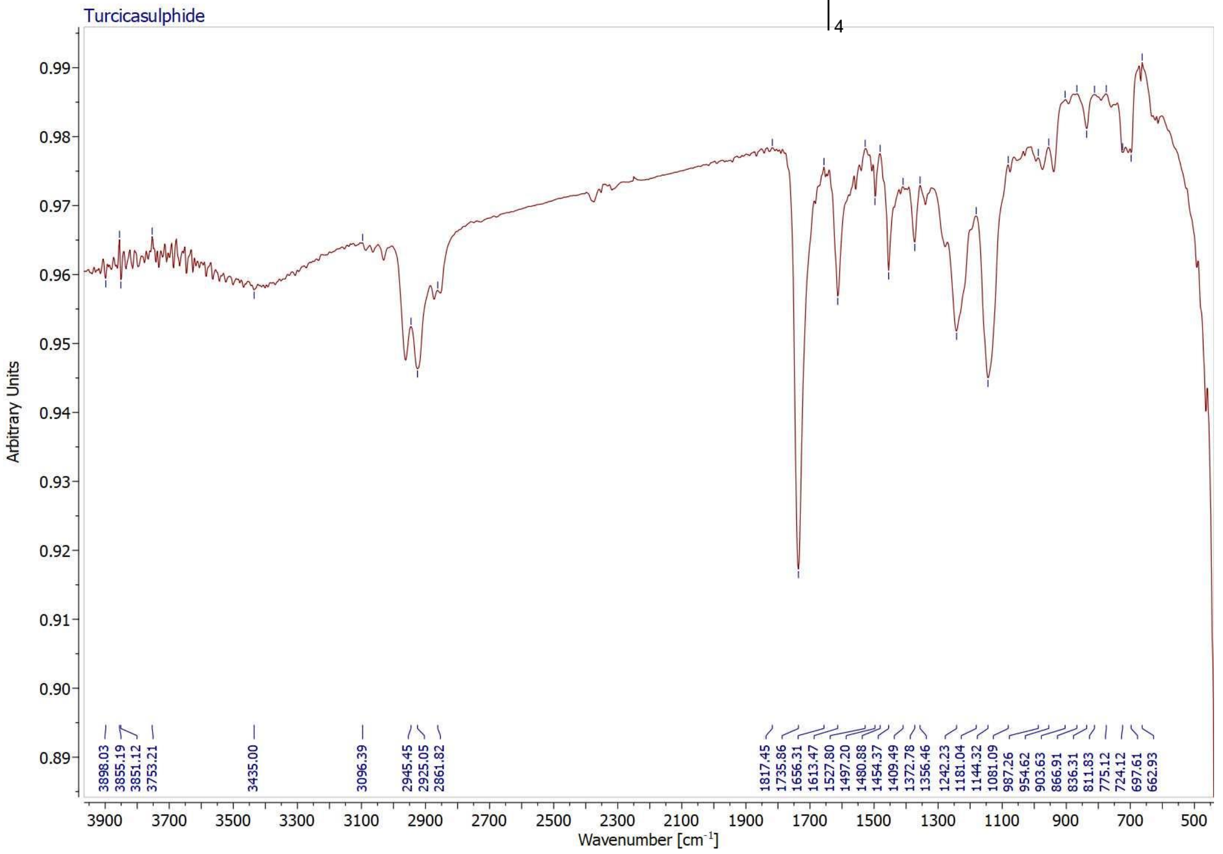

Figure S65 IR spectrum (NaCl) of turcicasulphide (**7**)

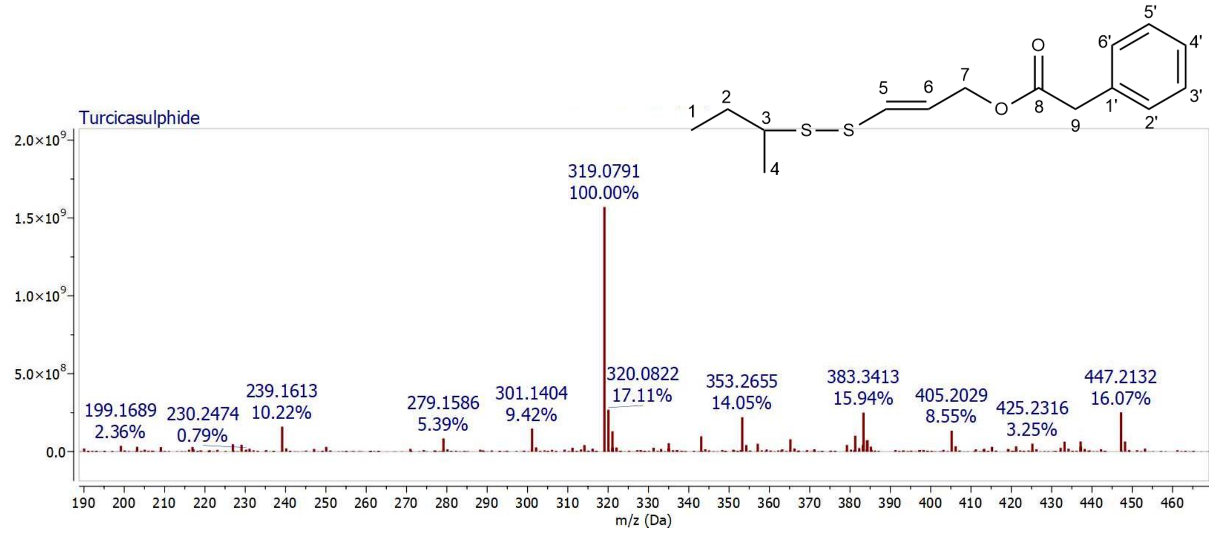

Figure S66 HRMS of turcicasulphide (**7**)

m/z $[M+Na]^+$ 319.0791 (calculated: 319.0802)

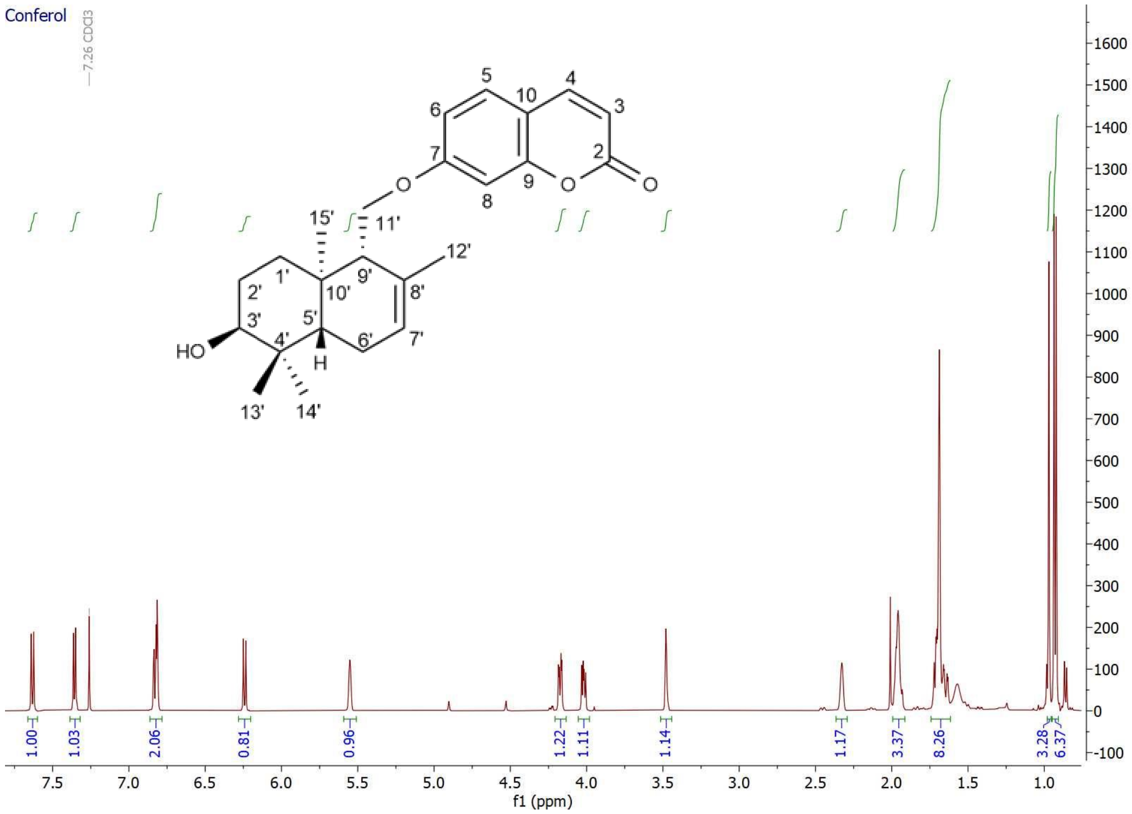

Figure S67 ^{1}H-NMR spectrum (600 MHz, $CDCl_3$) of conferol (**8**)

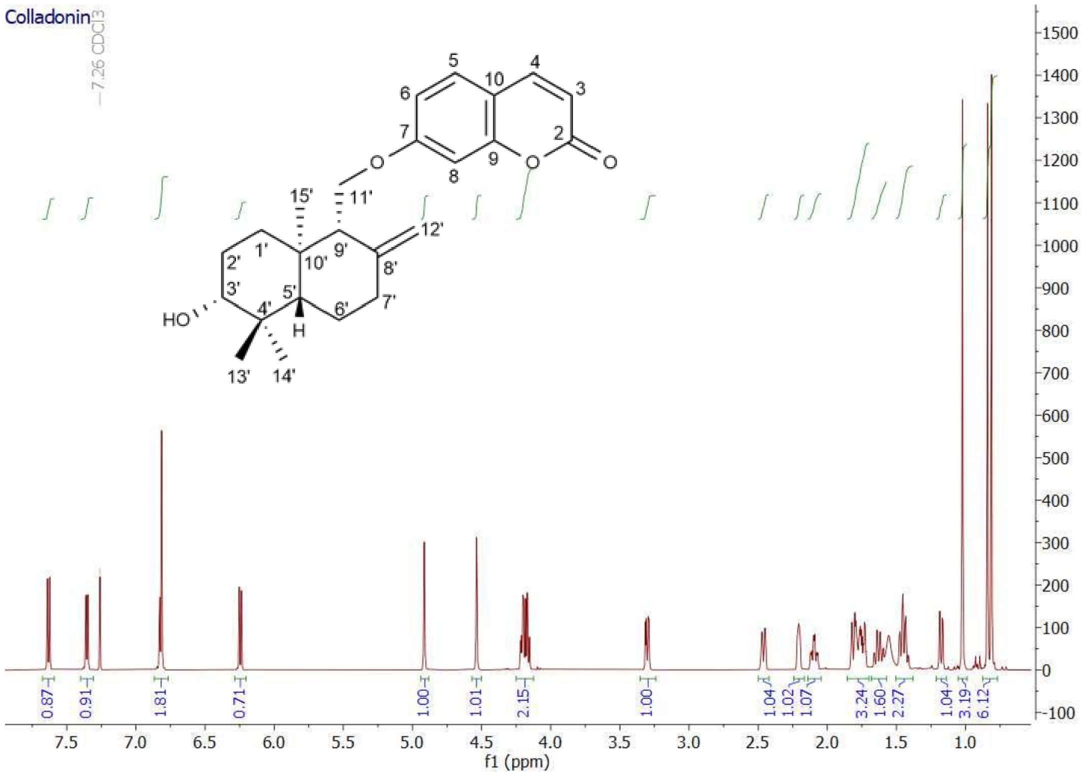

Figure S68 ^{1}H-NMR spectrum (600 MHz, $CDCl_3$) of colladonin (**9**)

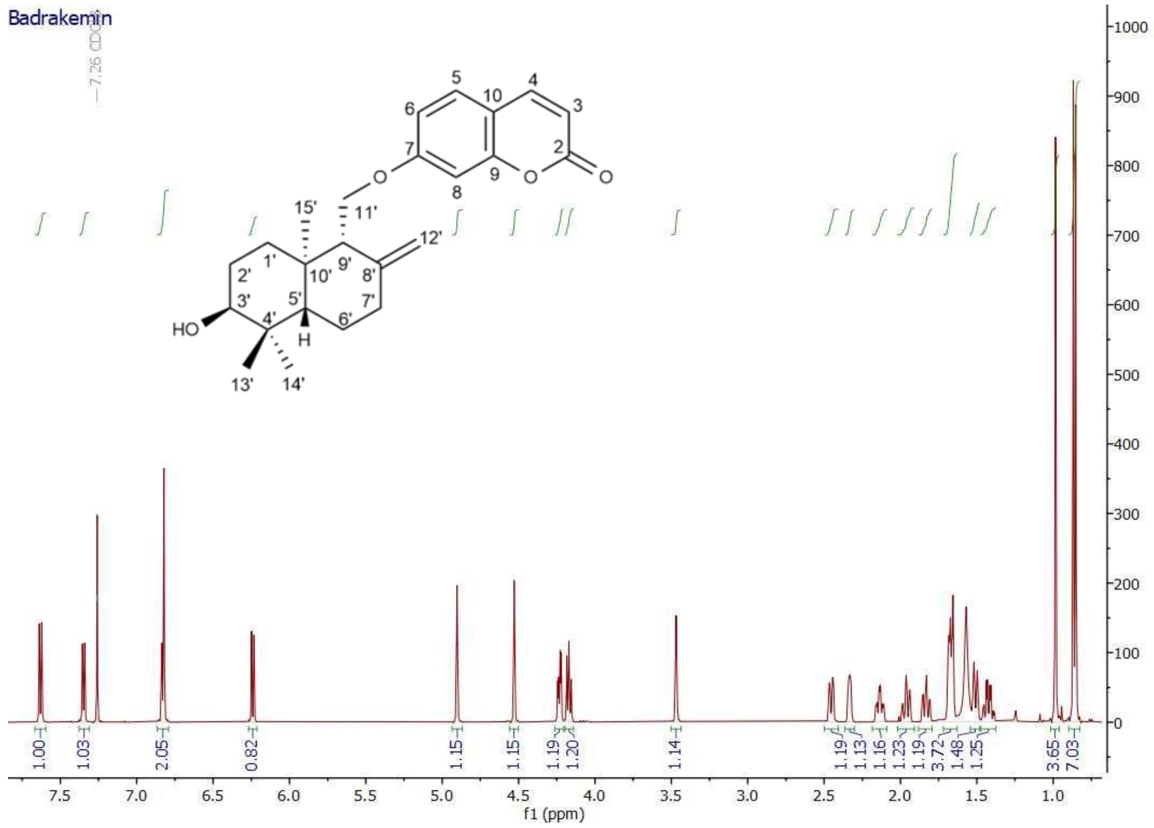

Figure S69 ^{1}H-NMR spectrum (600 MHz, $CDCl_3$) of badrakemin (**10**)

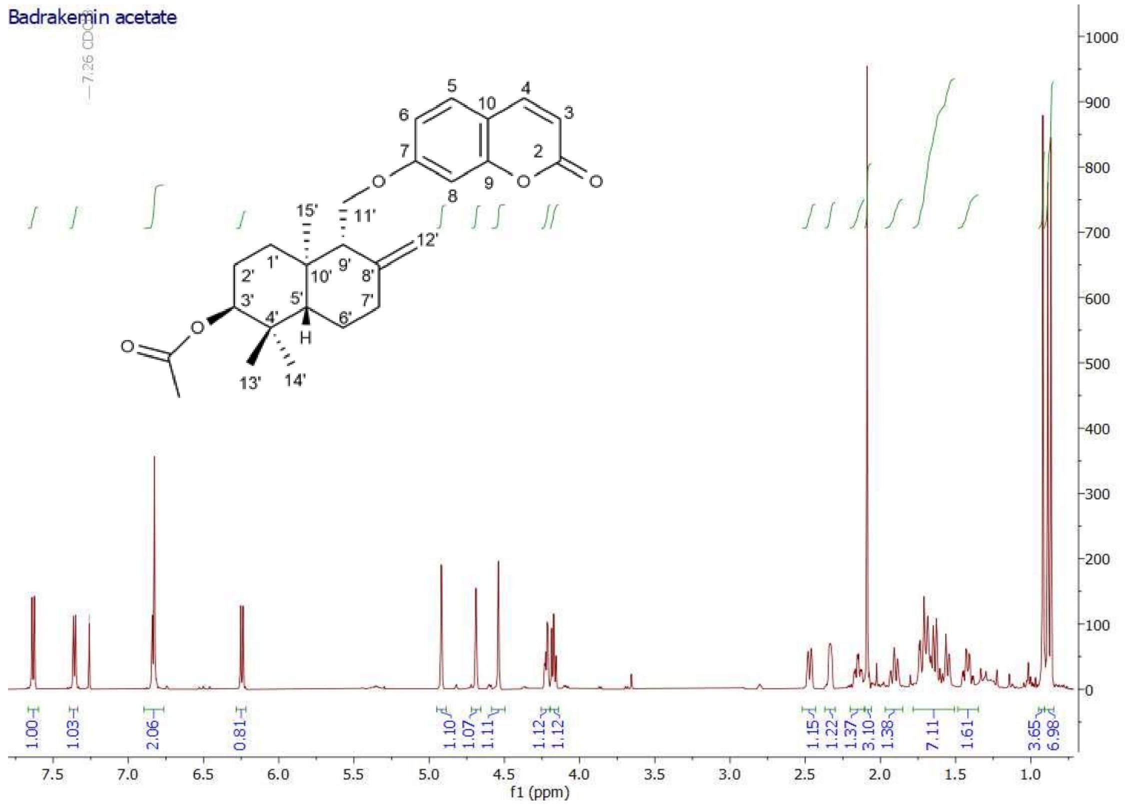

Figure S70 ^{1}H-NMR spectrum (600 MHz, $CDCl_3$) of badrakemin acetate (**11**)

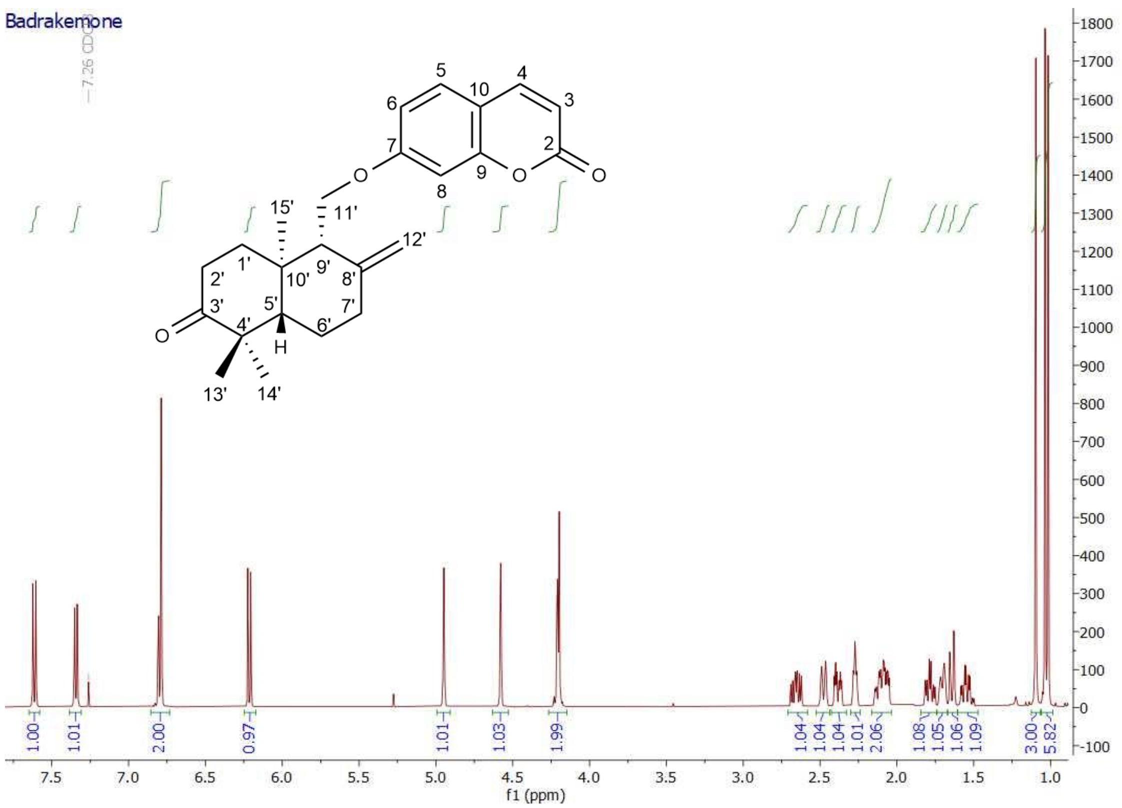

Figure S71 ^{1}H-NMR spectrum (600 MHz, $CDCl_3$) of badrakemone (**12**)

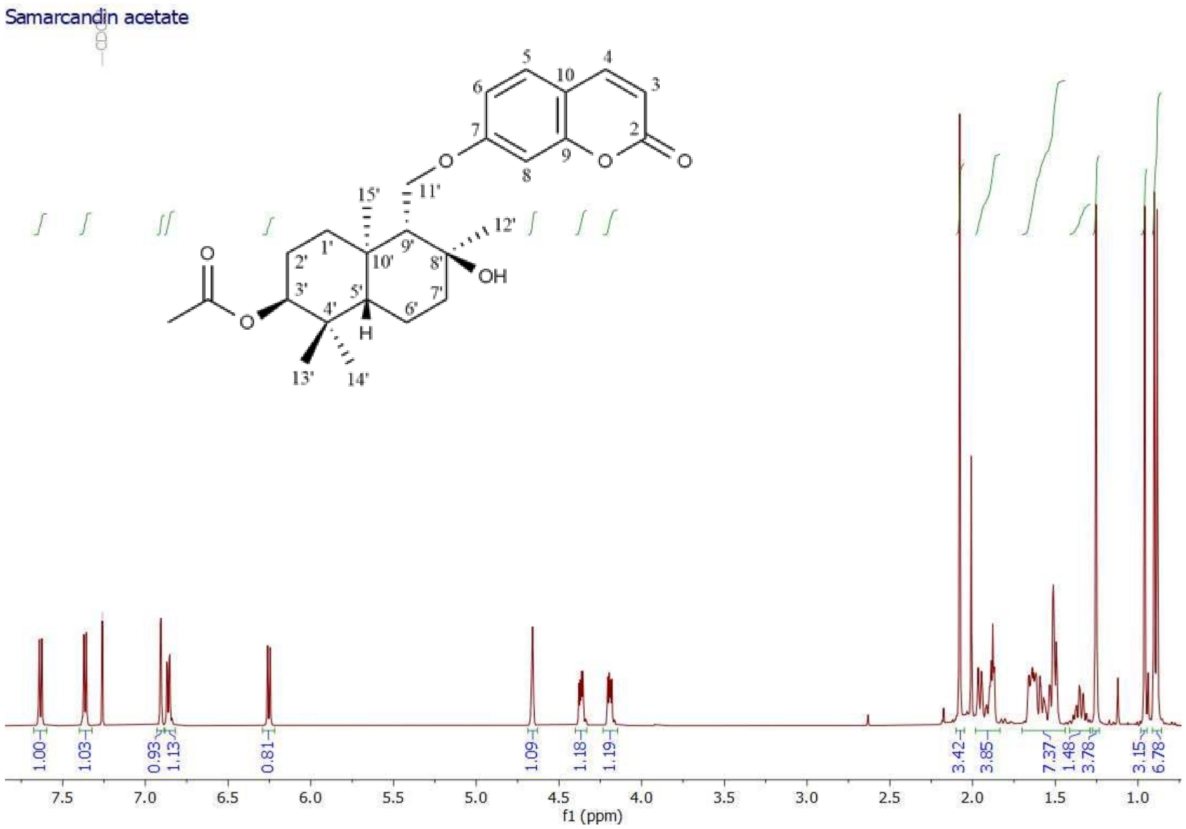

Figure S72 ^{1}H-NMR spectrum (600 MHz, $CDCl_3$) of samarcandin acetate (**13**)

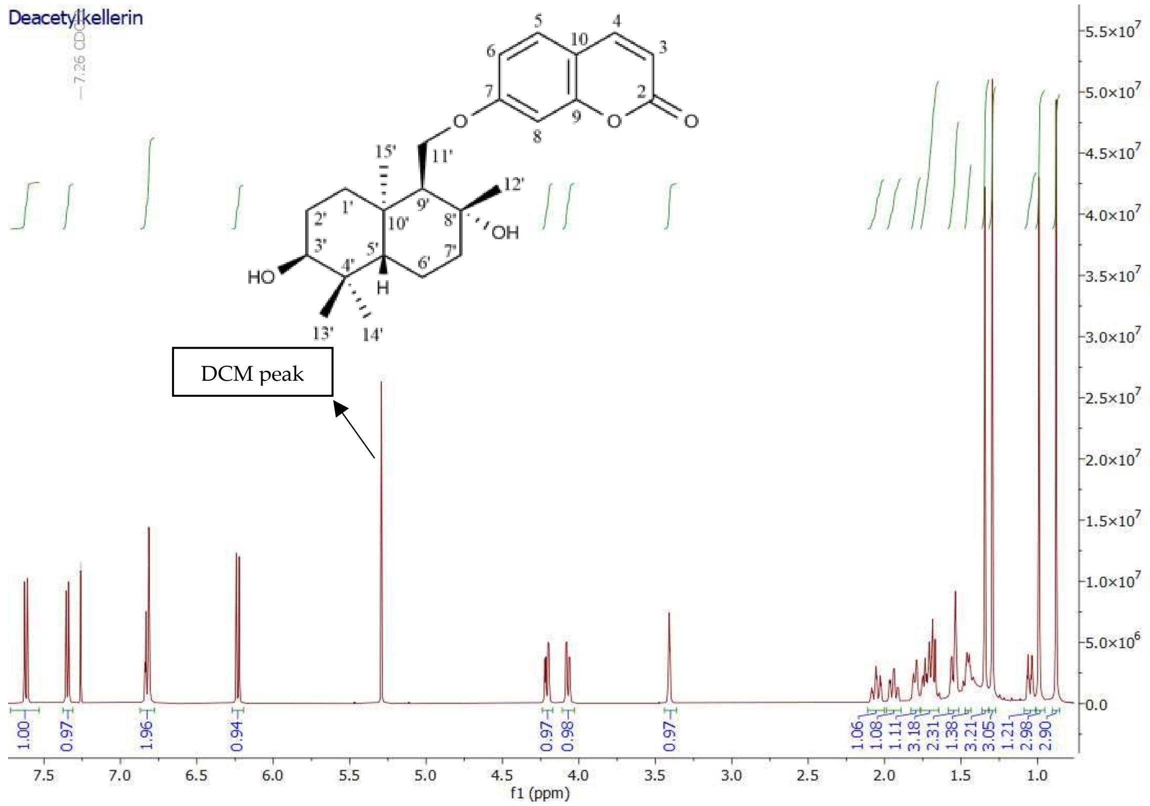

Figure S73 ^{1}H-NMR spectrum (600 MHz, $CDCl_3$) of deacetylkellerin (**14**)

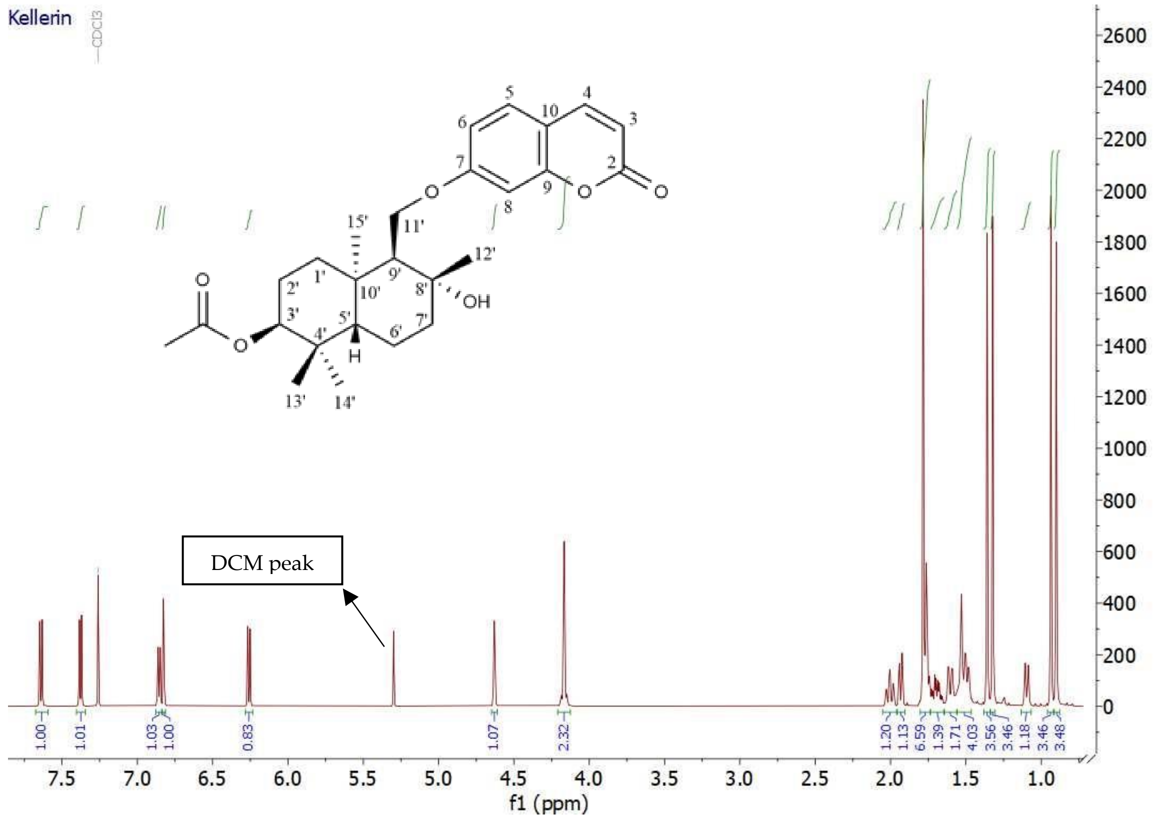

Figure S74 ^{1}H-NMR spectrum (600 MHz, $CDCl_3$) of kellerin (**15**)

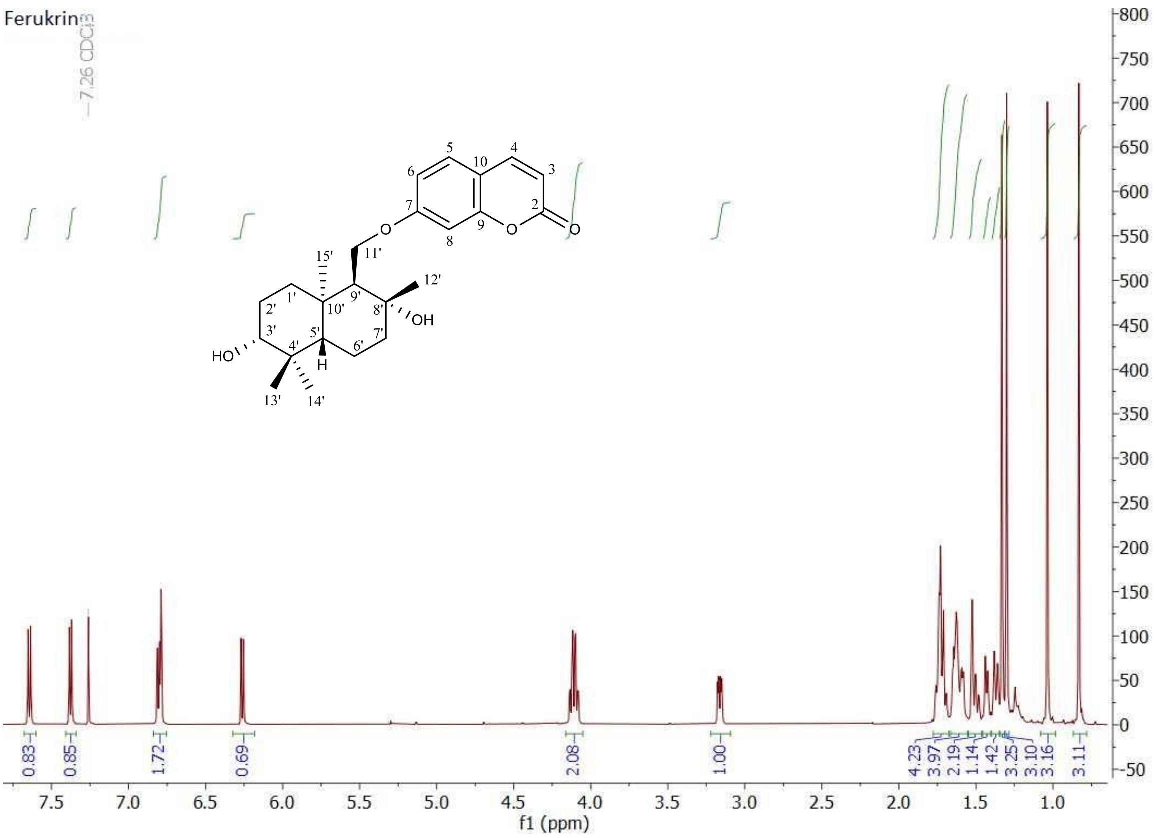

Figure S75 ^{1}H-NMR spectrum (600 MHz, $CDCl_3$) of ferukrin (**16**)

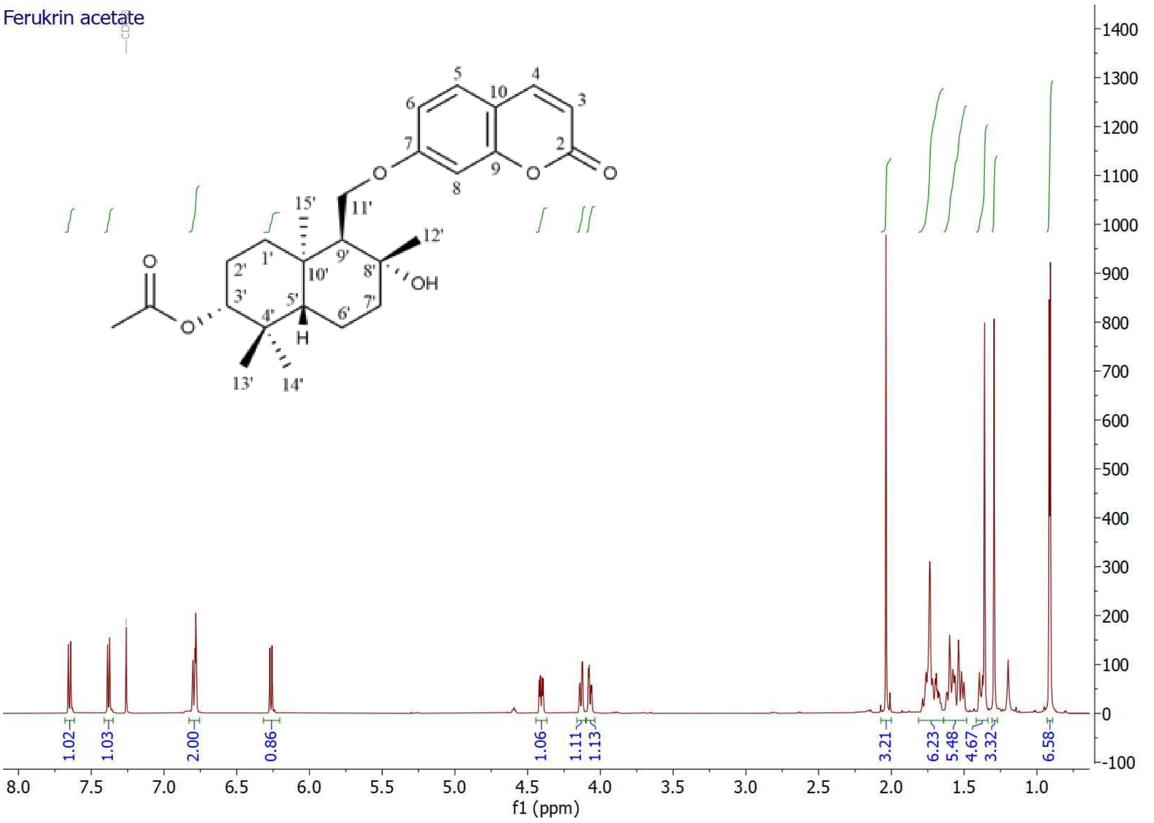

Figure S76 ^{1}H-NMR spectrum (600 MHz, $CDCl_3$) of ferukrin acetate (**17**)

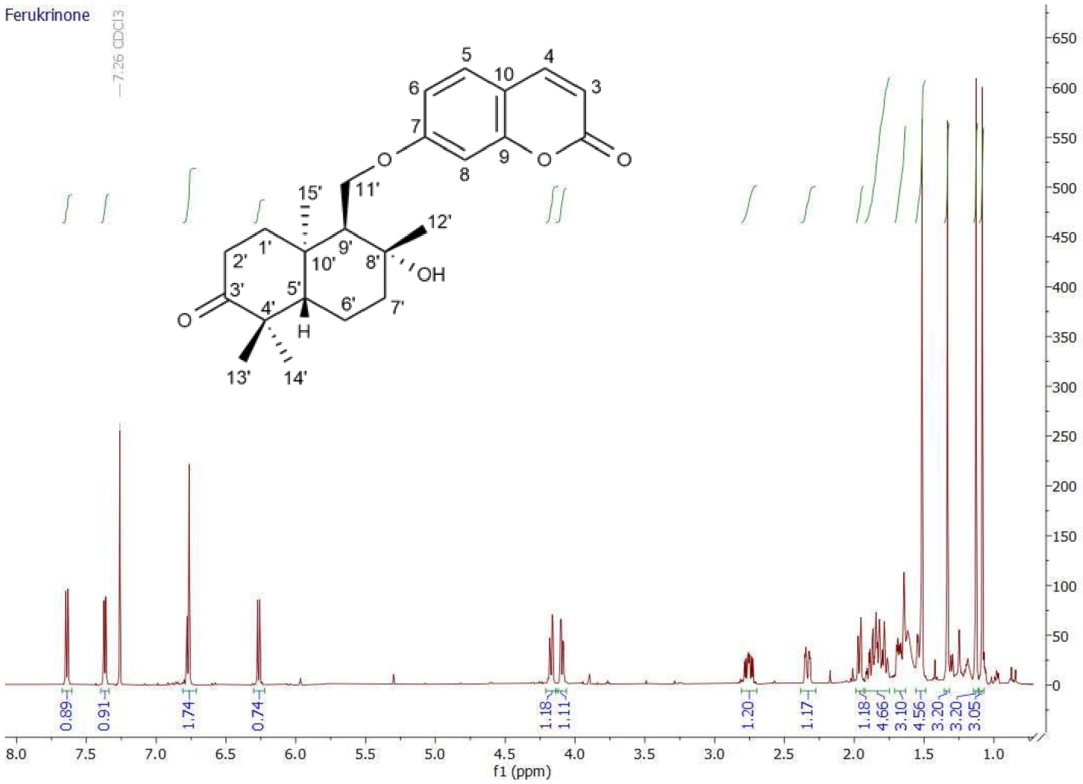

Figure S77 ^{1}H-NMR spectrum (600 MHz, $CDCl_3$) of ferukrinone (**18**)

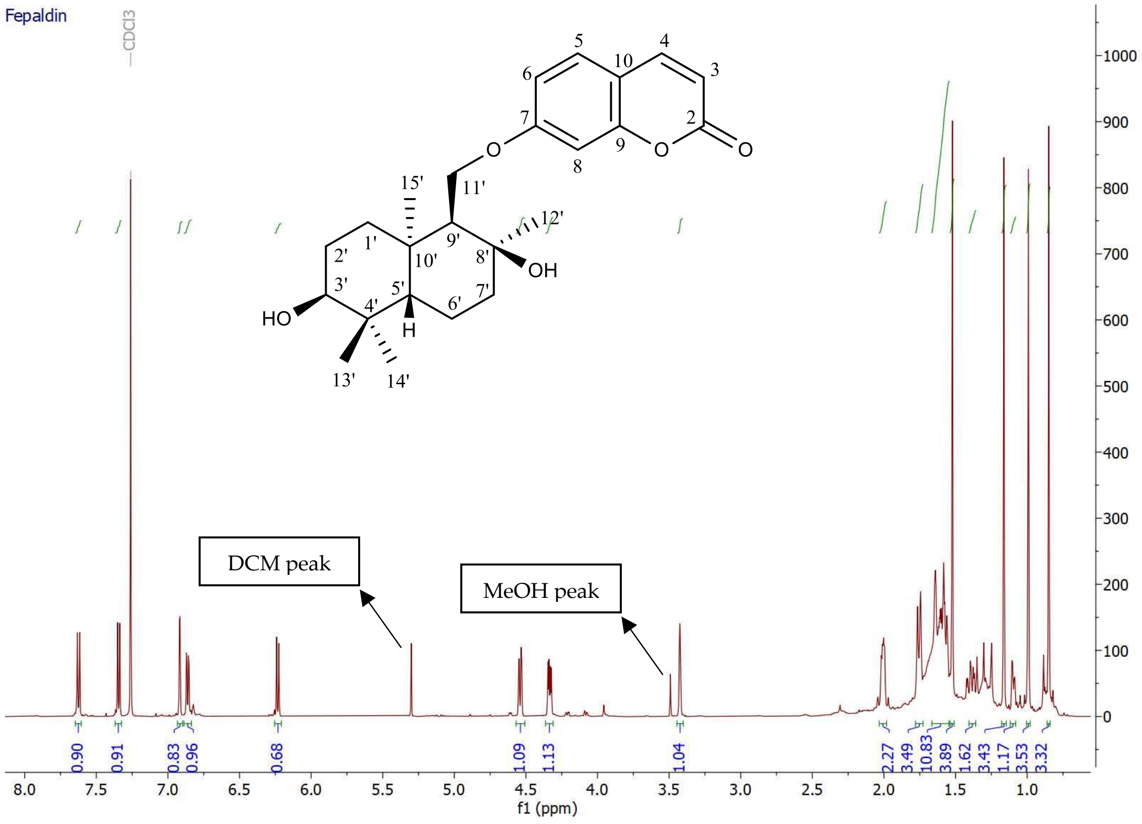

Figure S78 ^{1}H-NMR spectrum (600 MHz, $CDCl_3$) of fepaldin (**19**)

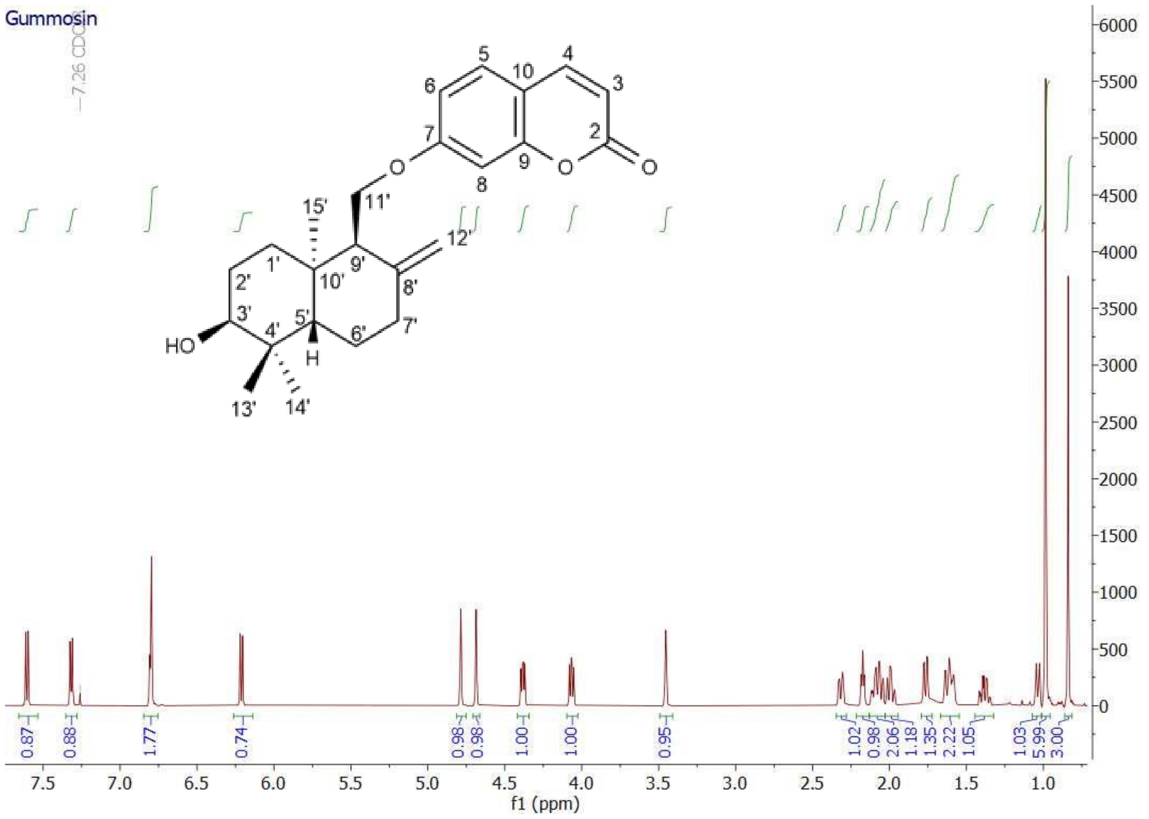

Figure S79 ^{1}H-NMR spectrum (600 MHz, $CDCl_3$) of gummosin (**20**)

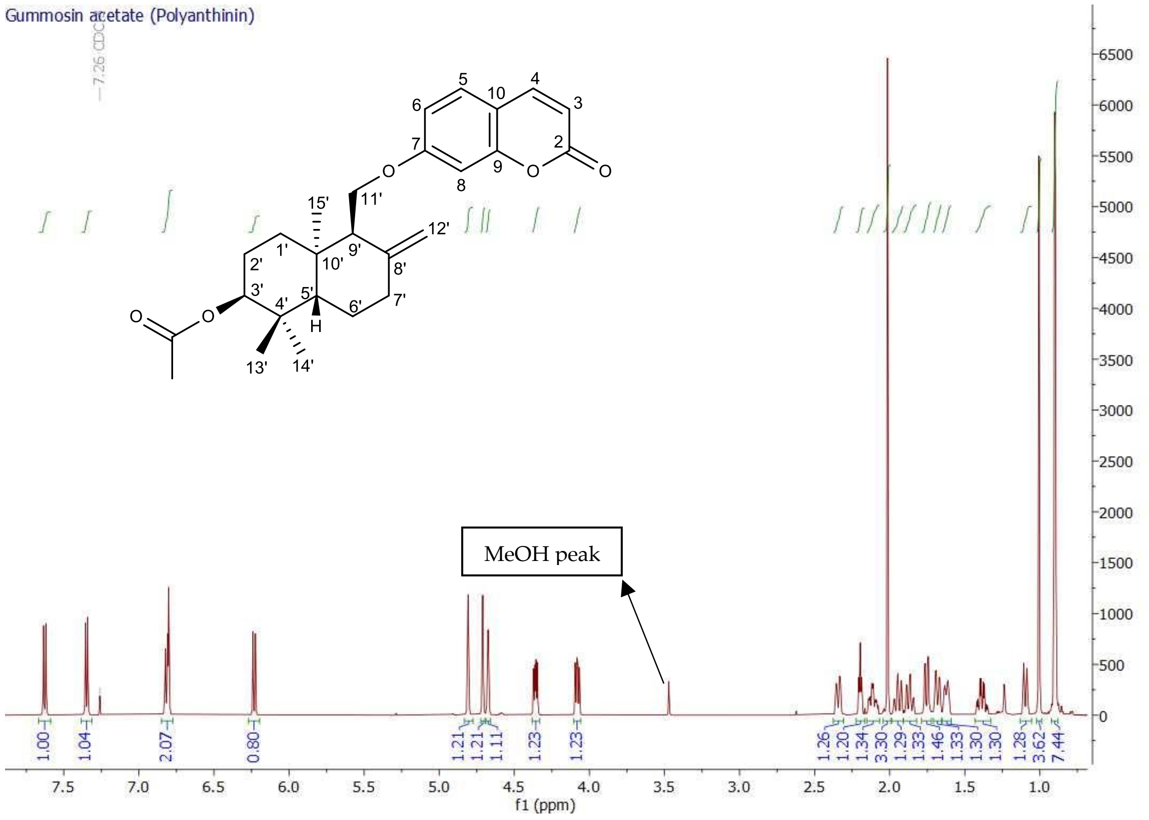

Figure S80 ^{1}H-NMR spectrum (600 MHz, $CDCl_3$) of gummosin acetate (**21**)

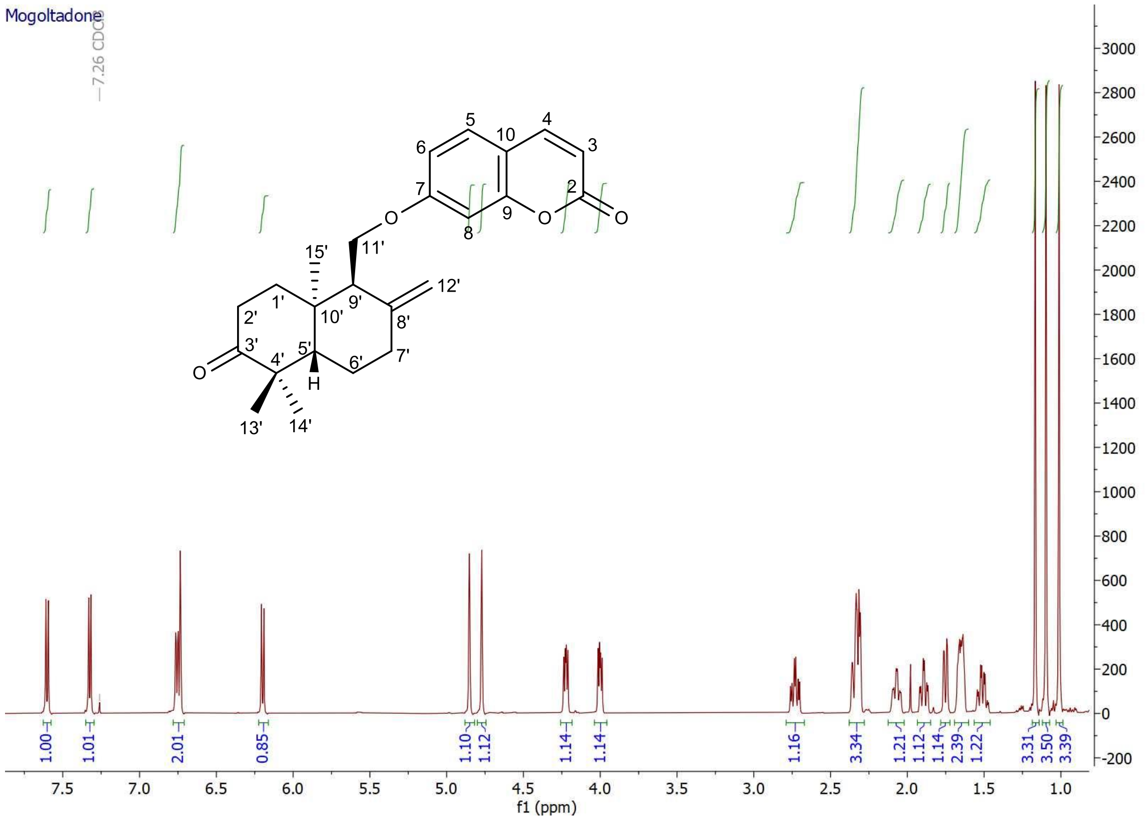

Figure S81 ^{1}H-NMR spectrum (600 MHz, $CDCl_3$) of mogoltadone (**22**)

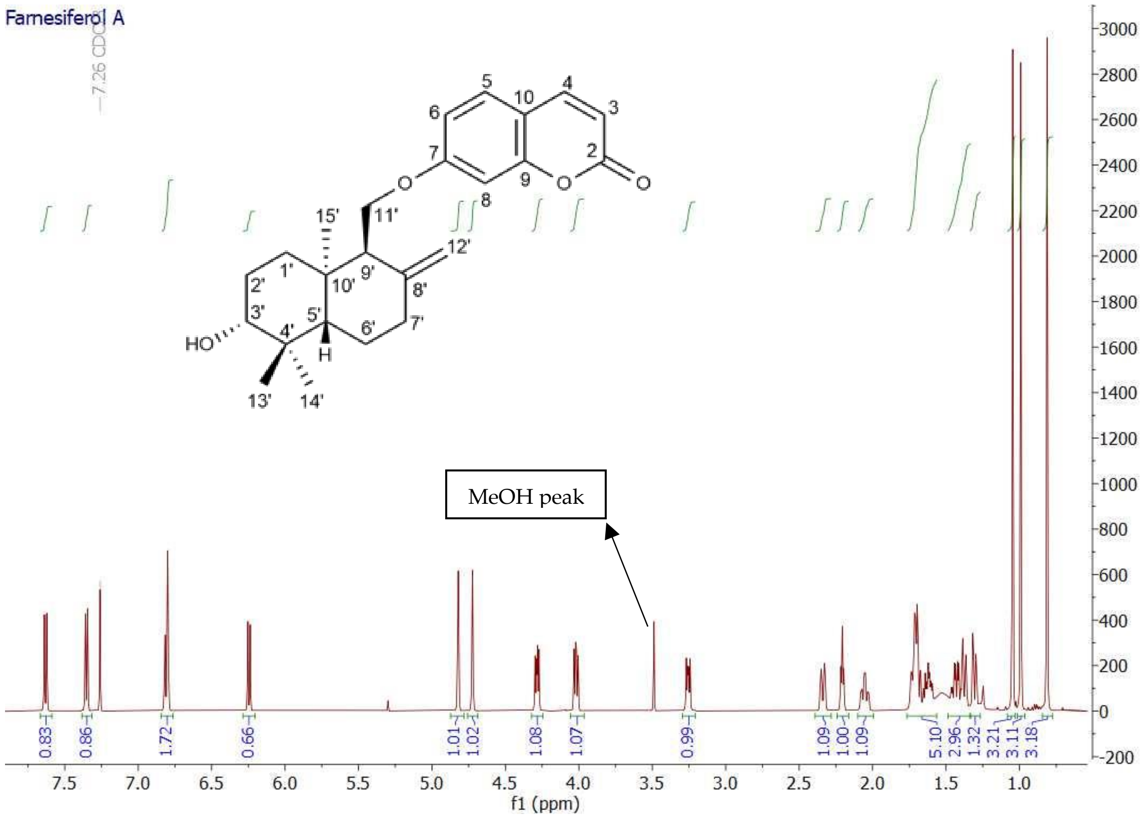

Figure S82 ^{1}H-NMR spectrum (600 MHz, $CDCl_3$) of farnesiferol A (**23**)

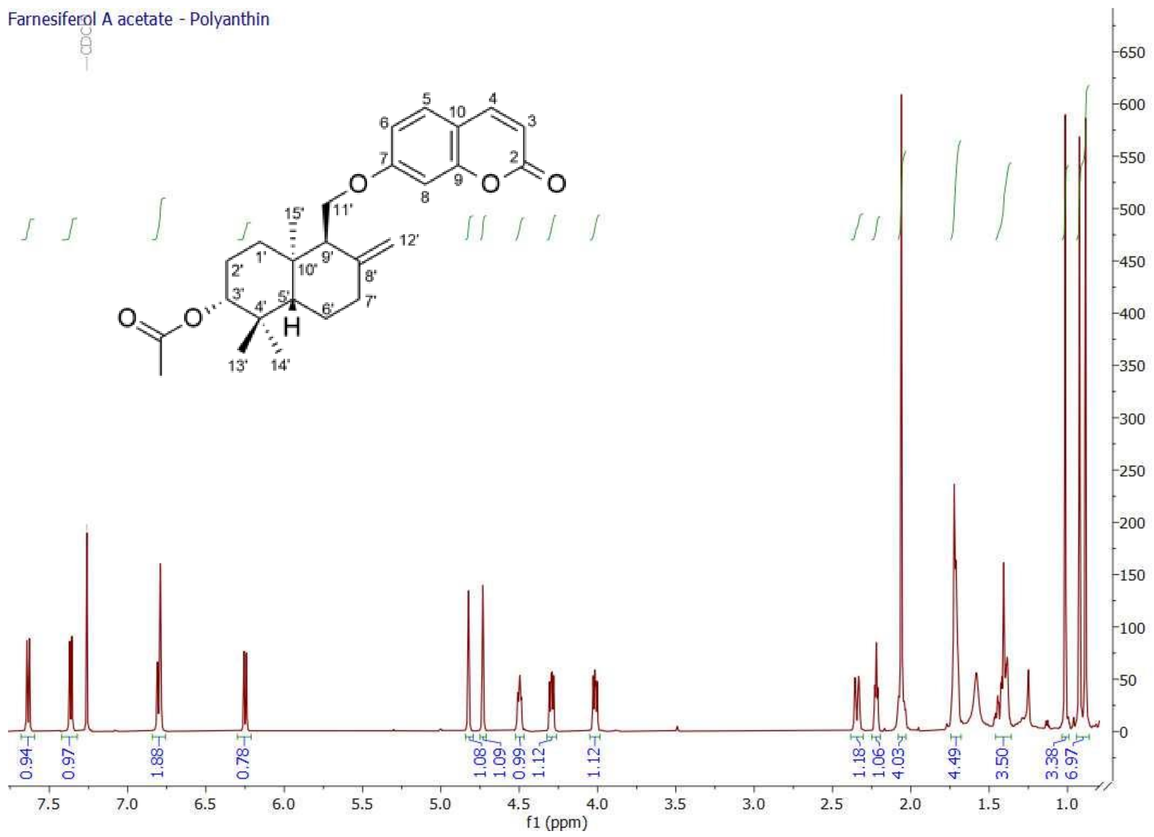

Figure S83 ^{1}H-NMR spectrum (600 MHz, $CDCl_3$) of farnesiferol A acetate (**24**)

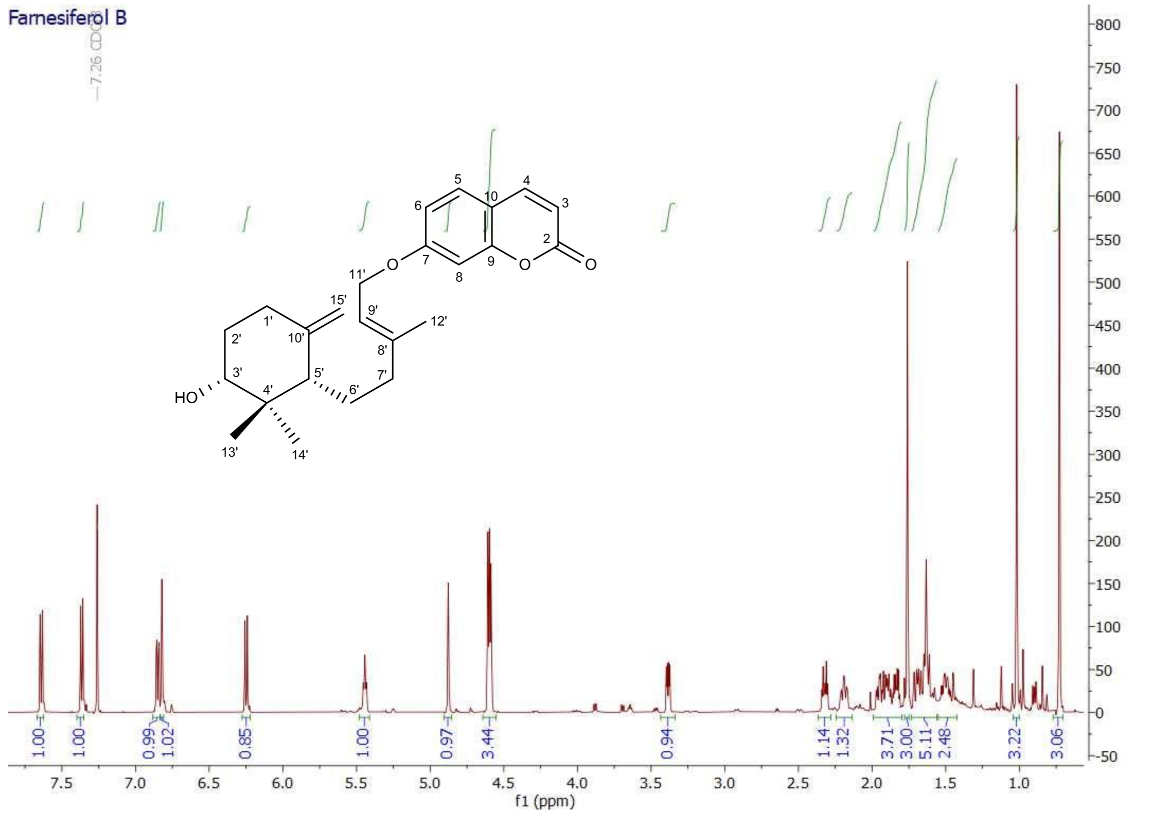

Figure S84 ^{1}H-NMR spectrum (600 MHz, $CDCl_3$) of farnesiferol B (**25**)

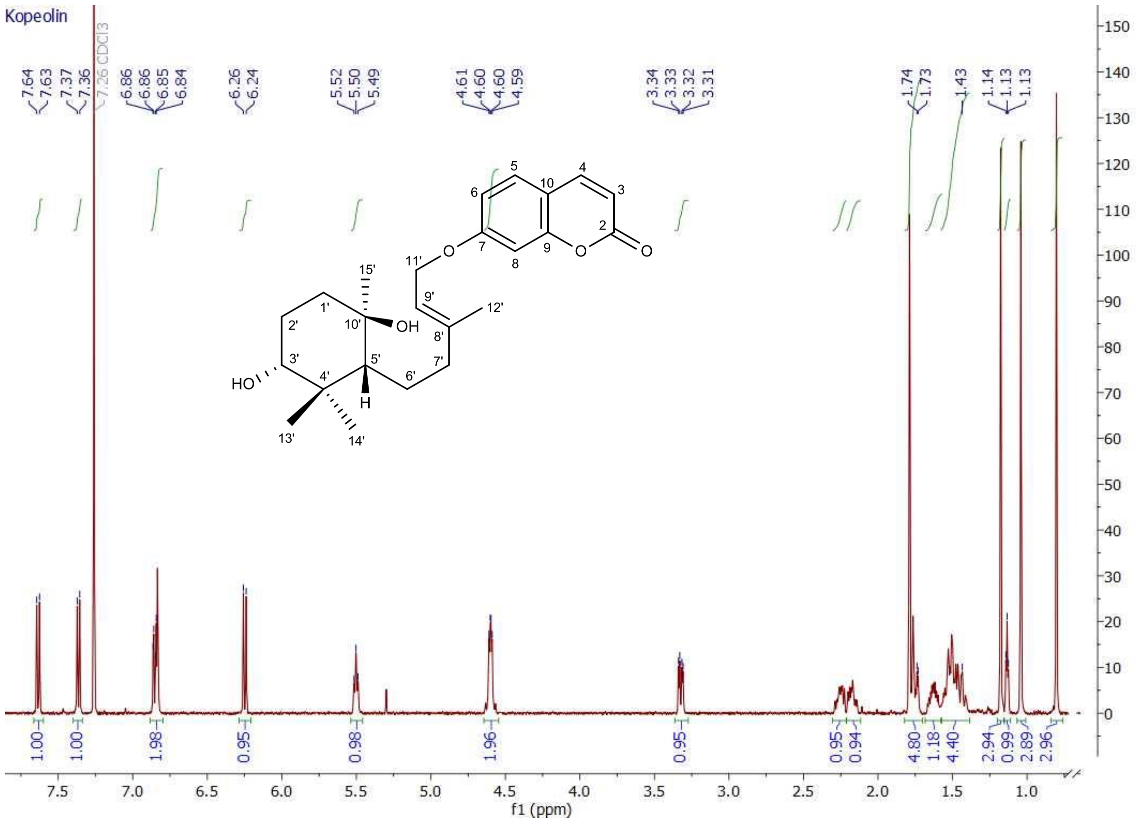

Figure S85 ^{1}H-NMR spectrum (600 MHz, $CDCl_3$) of kopeolin (**26**)

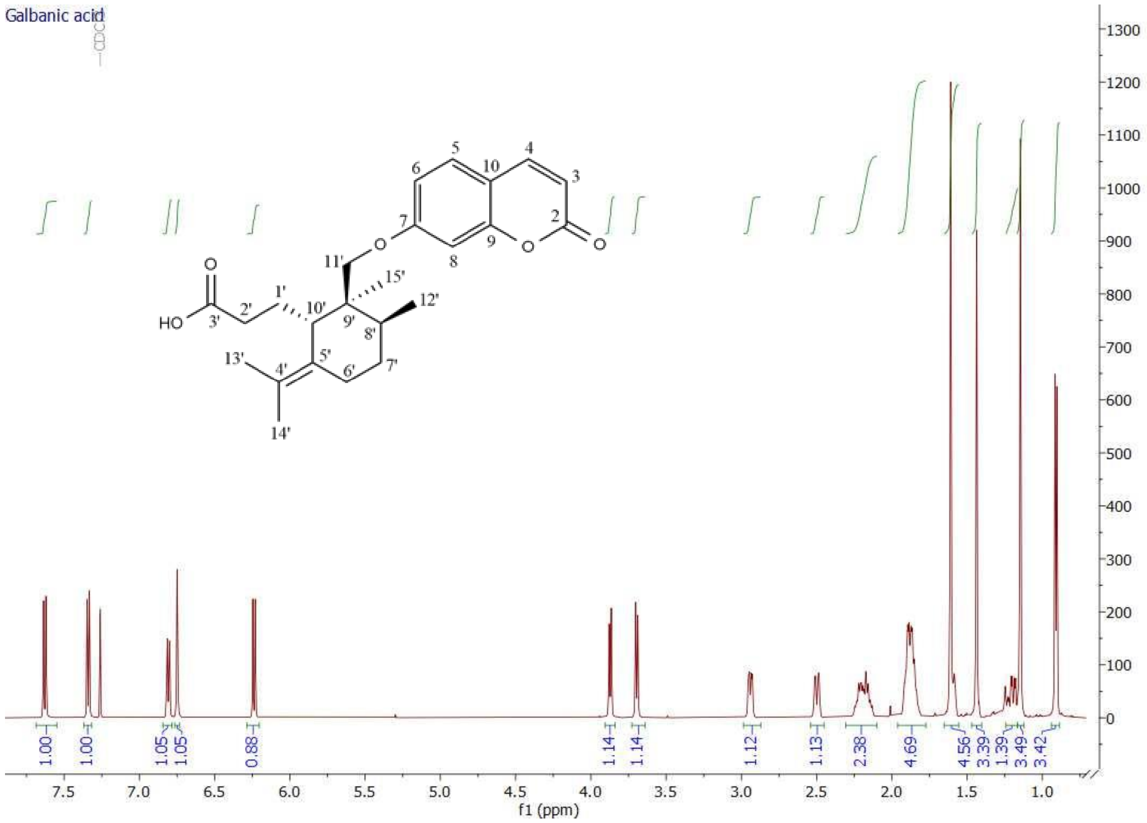

Figure S86 ^{1}H-NMR spectrum (600 MHz, $CDCl_3$) of galbanic acid (**27**)

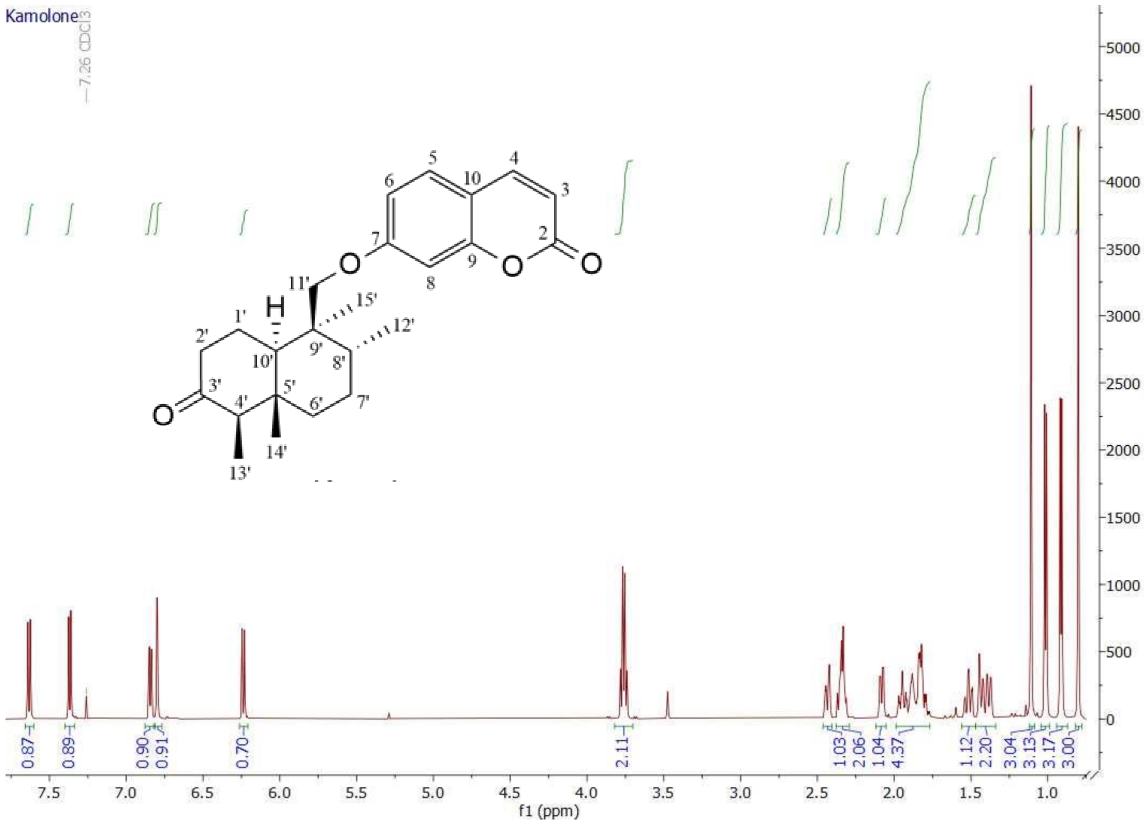

Figure S87 ^{1}H-NMR spectrum (600 MHz, $CDCl_3$) of kamolone (**28**)

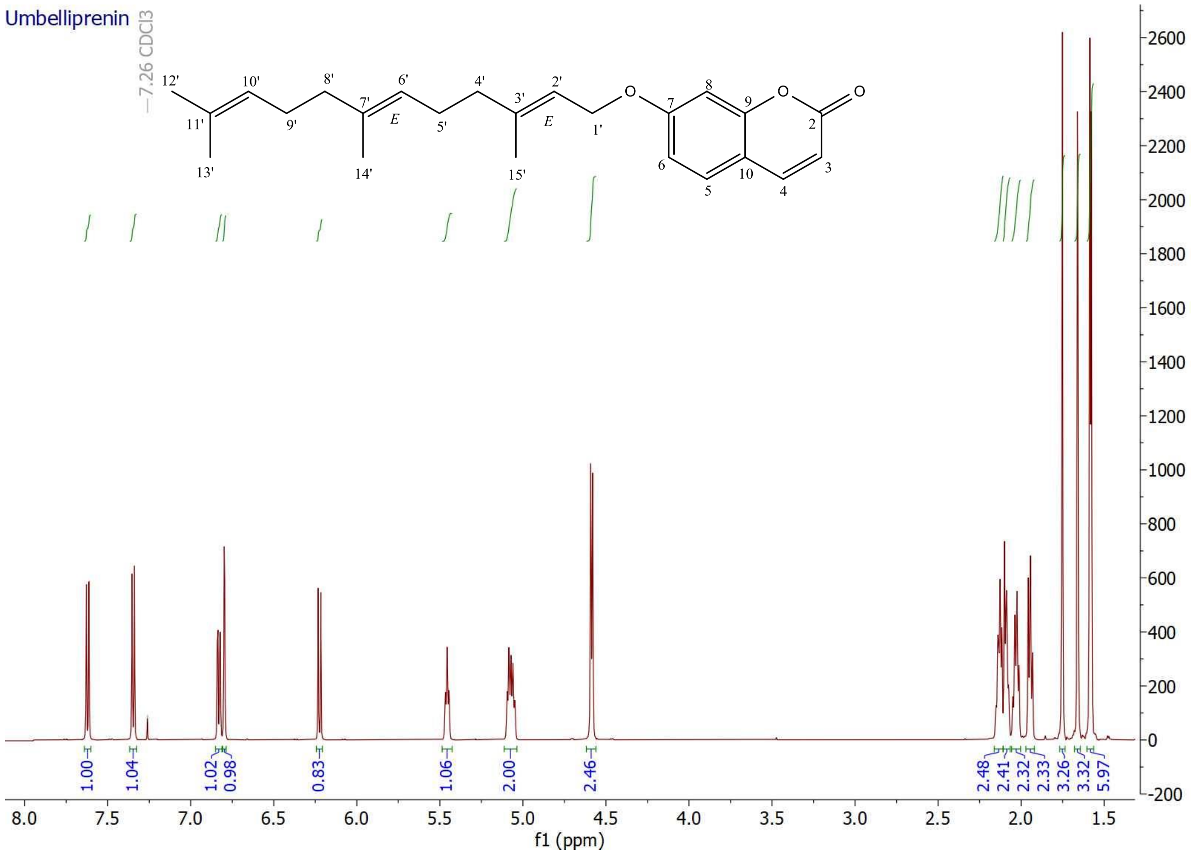

Figure S88 ^{1}H-NMR spectrum (600 MHz, $CDCl_3$) of umbelliprenin (**29**)

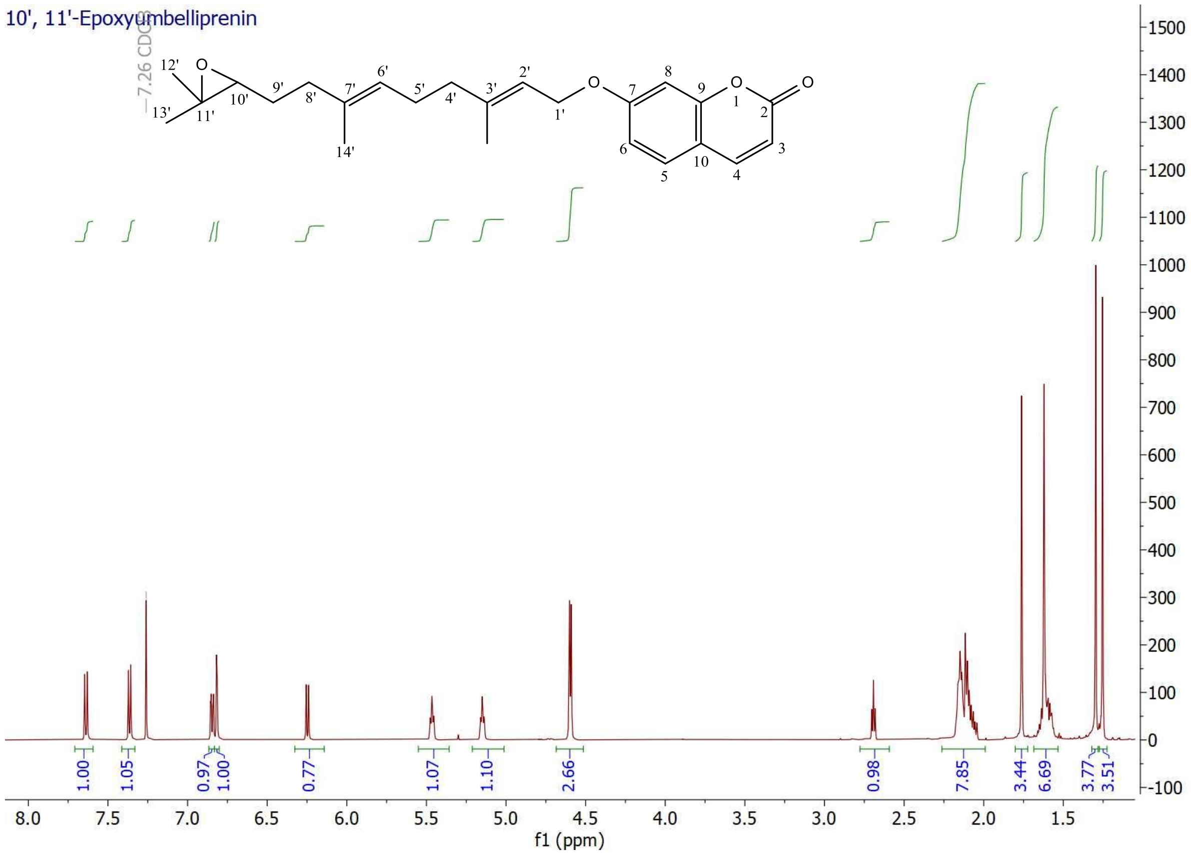

Figure S89 ^{1}H-NMR spectrum (600 MHz, $CDCl_3$) of 10', 11'-epoxyumbelliprenin (**30**)

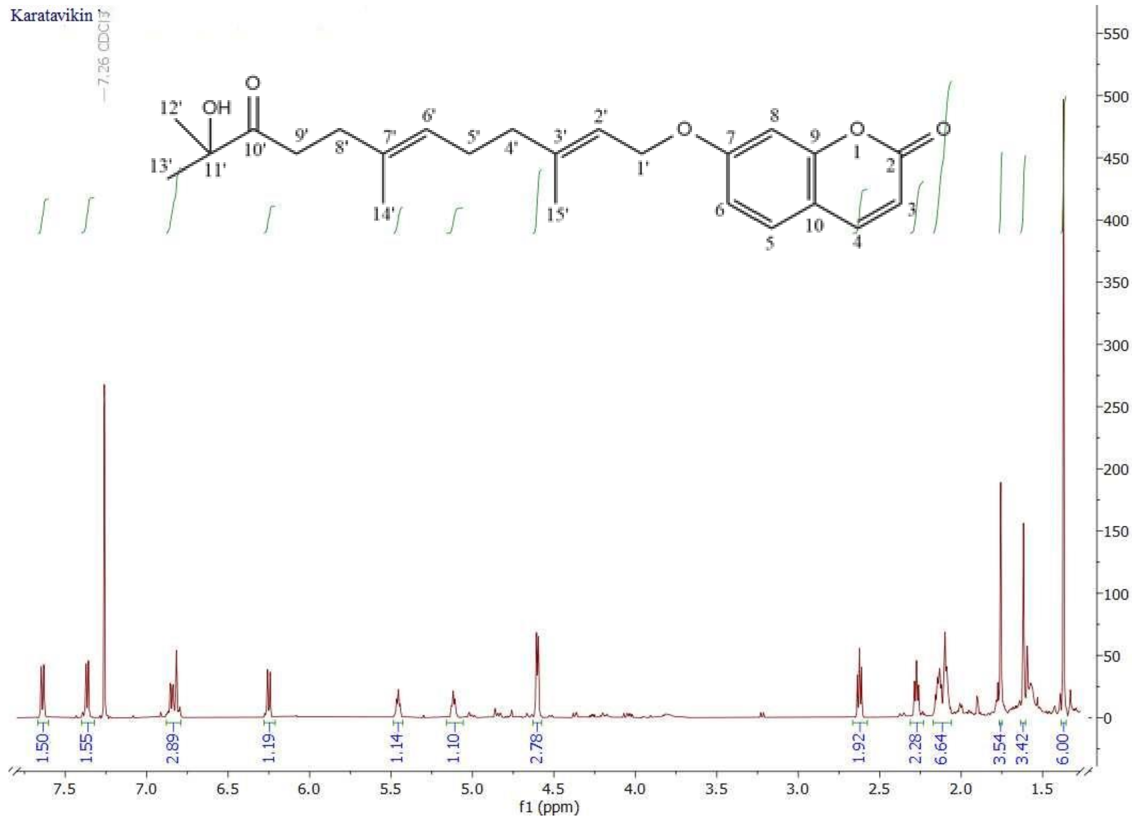

Figure S90 ^{1}H-NMR spectrum (600 MHz, $CDCl_3$) of karatavikin (**31**)

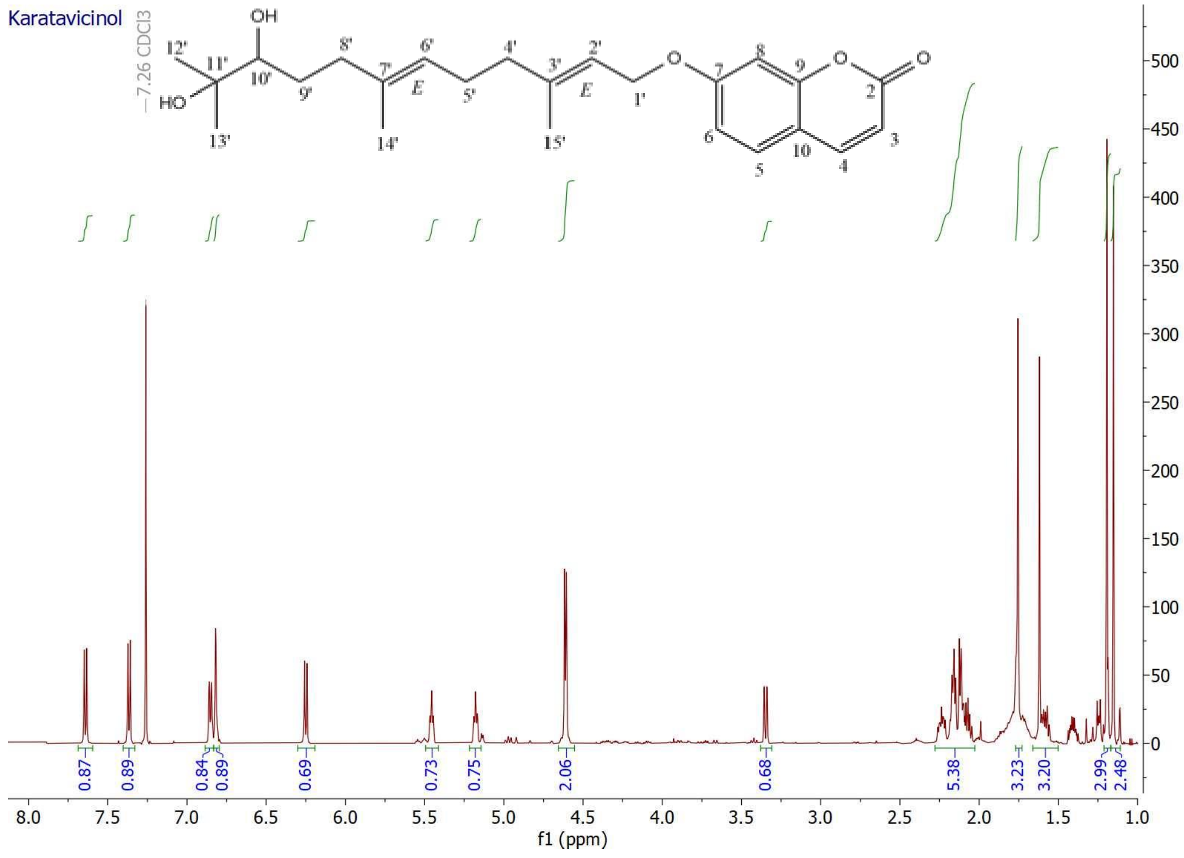

Figure S91 ^{1}H-NMR spectrum (600 MHz, $CDCl_3$) of karatavicinol (**32**)

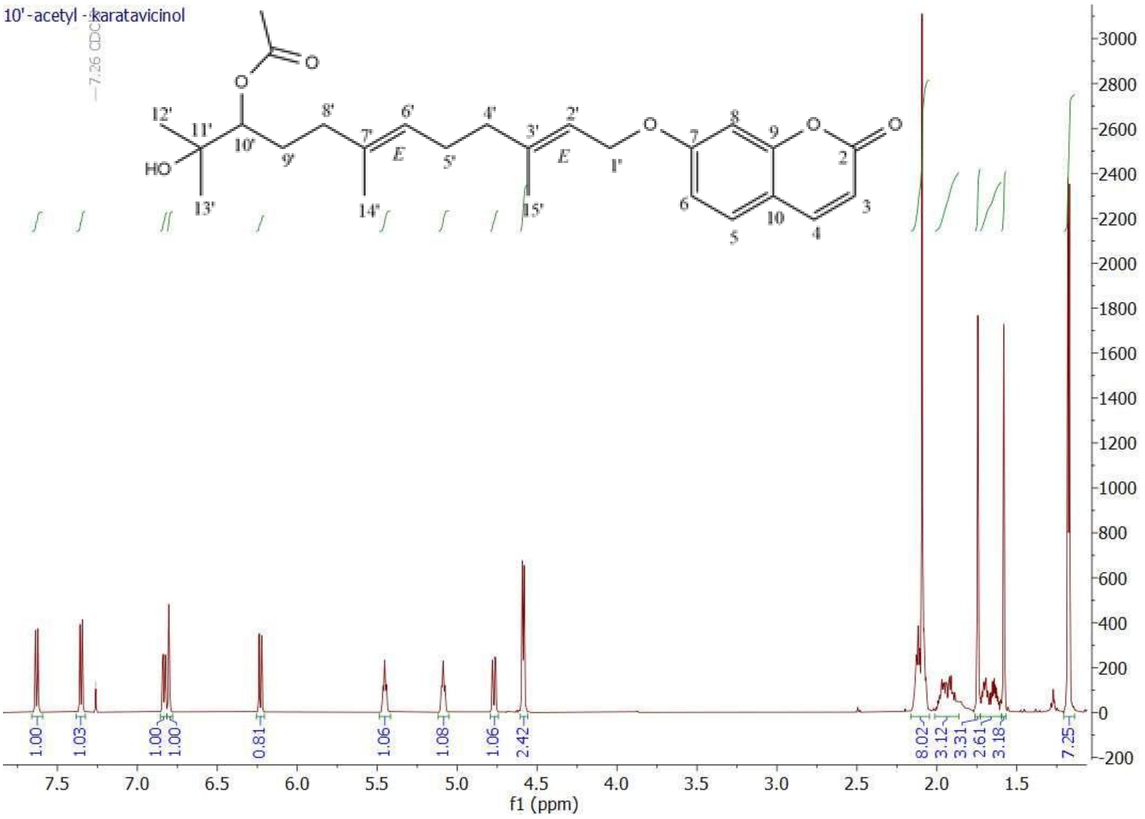

Figure S92 ^{1}H-NMR spectrum (600 MHz, $CDCl_3$) of 10′-acetylkaratavicinol (**33**)

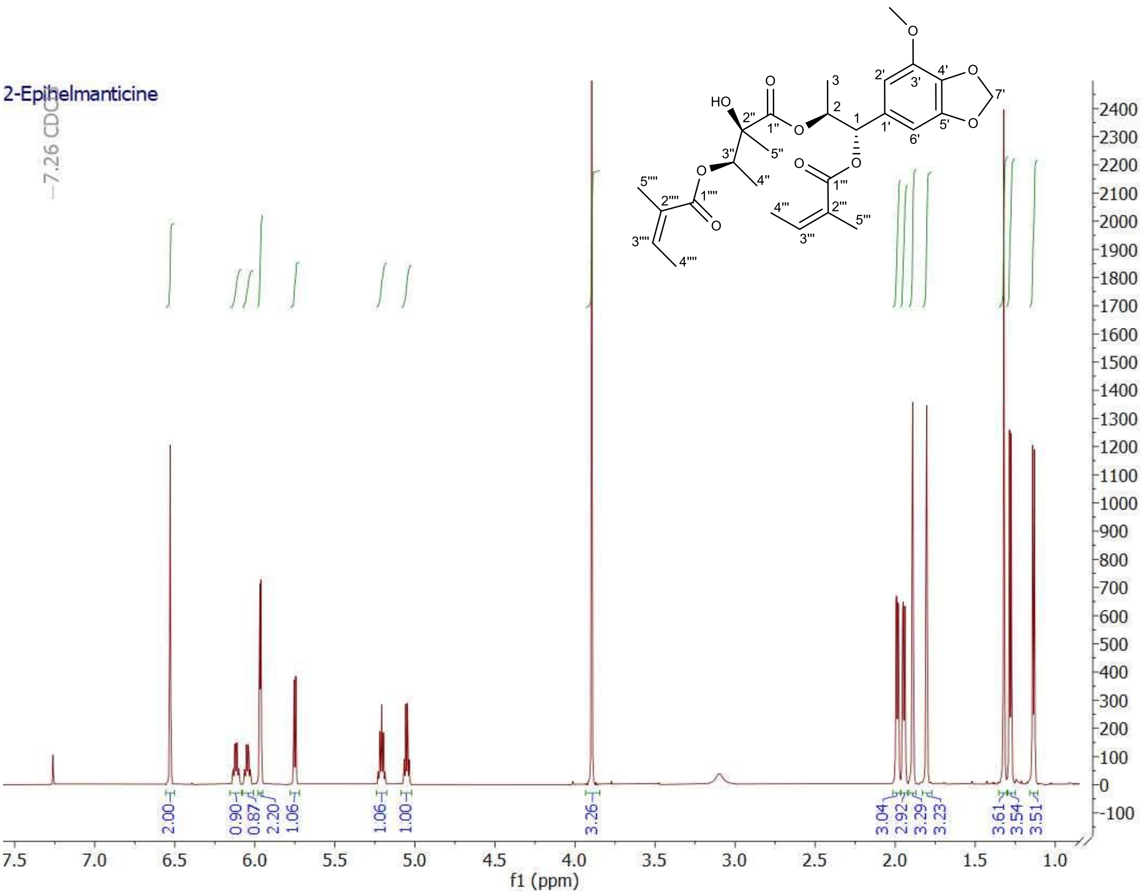

Figure S93 ^{1}H-NMR spectrum (600 MHz, $CDCl_3$) of 2-epihelmanticine (**34**)

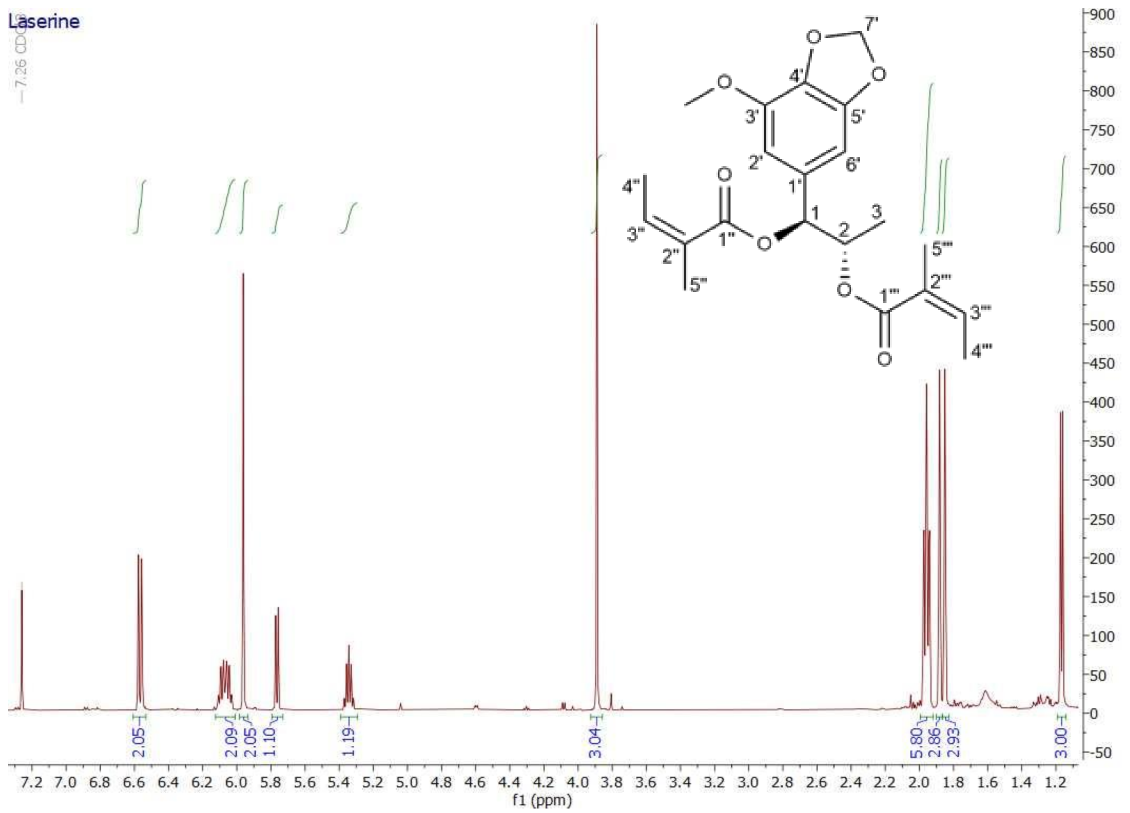

Figure S94 ^{1}H-NMR spectrum (600 MHz, $CDCl_3$) of laserine (**35**)

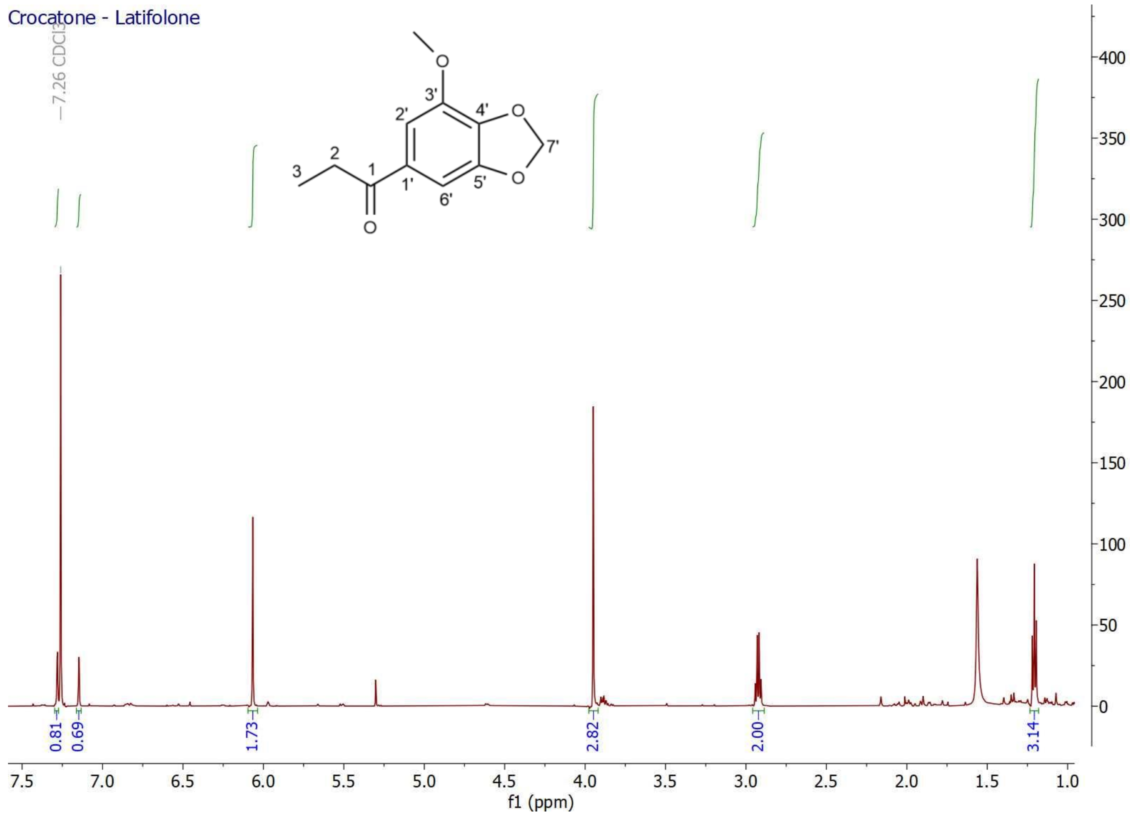

Figure S95 ^{1}H-NMR spectrum (600 MHz, $CDCl_3$) of crocatone (**36**)

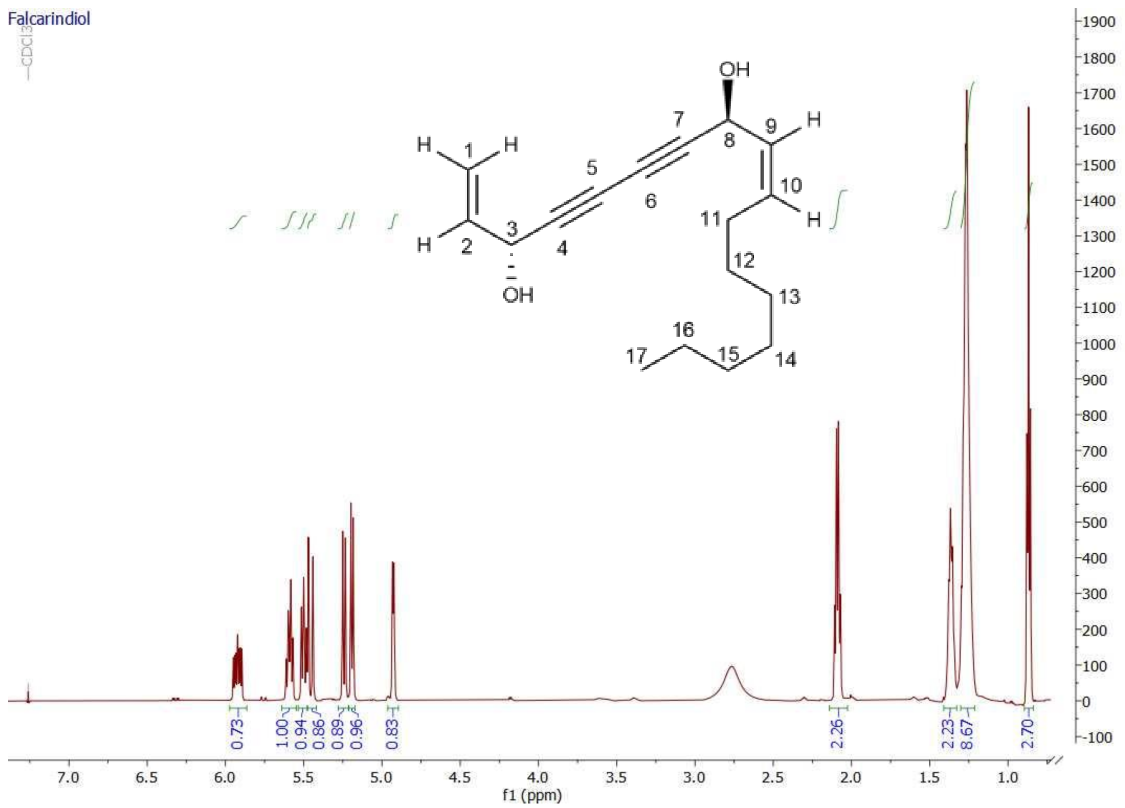

Figure S96 ^{1}H-NMR spectrum (600 MHz, $CDCl_3$) of falcarindiol (**37**)

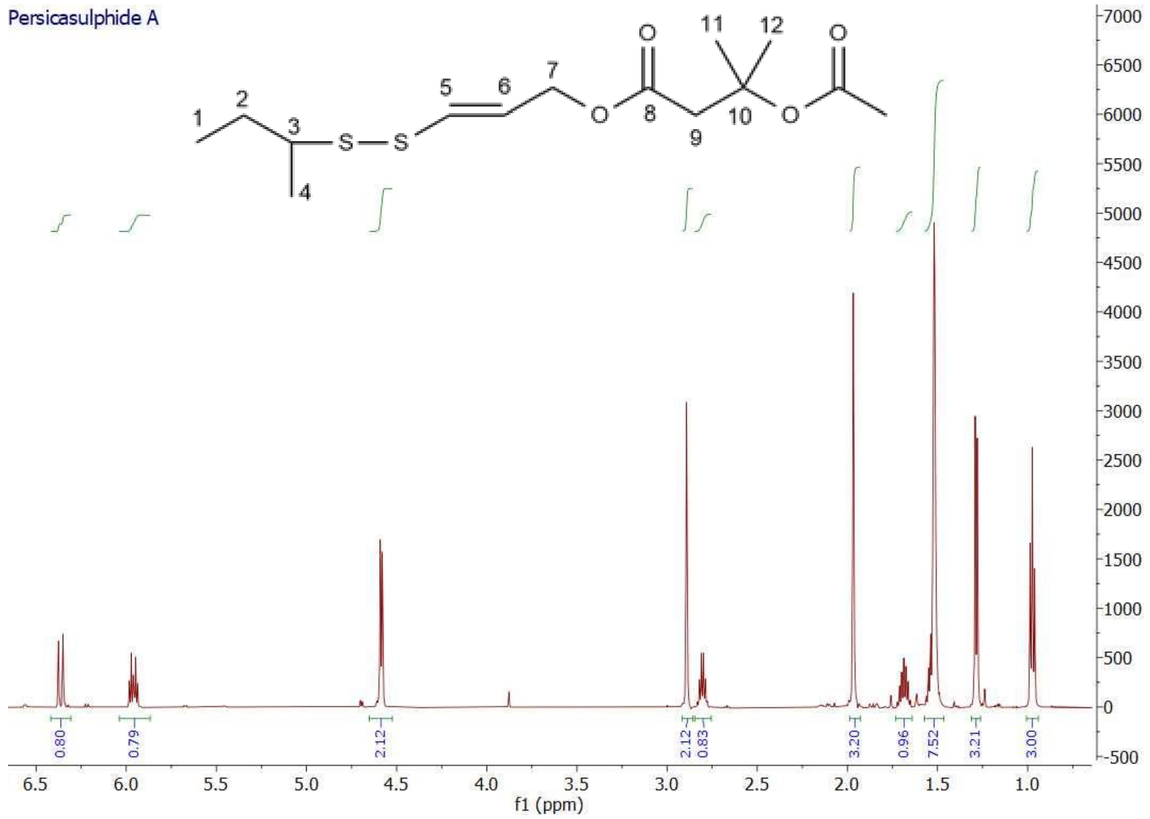

Figure S97 ^{1}H-NMR spectrum (600 MHz, $CDCl_3$) of persicasulphide A (**38**)

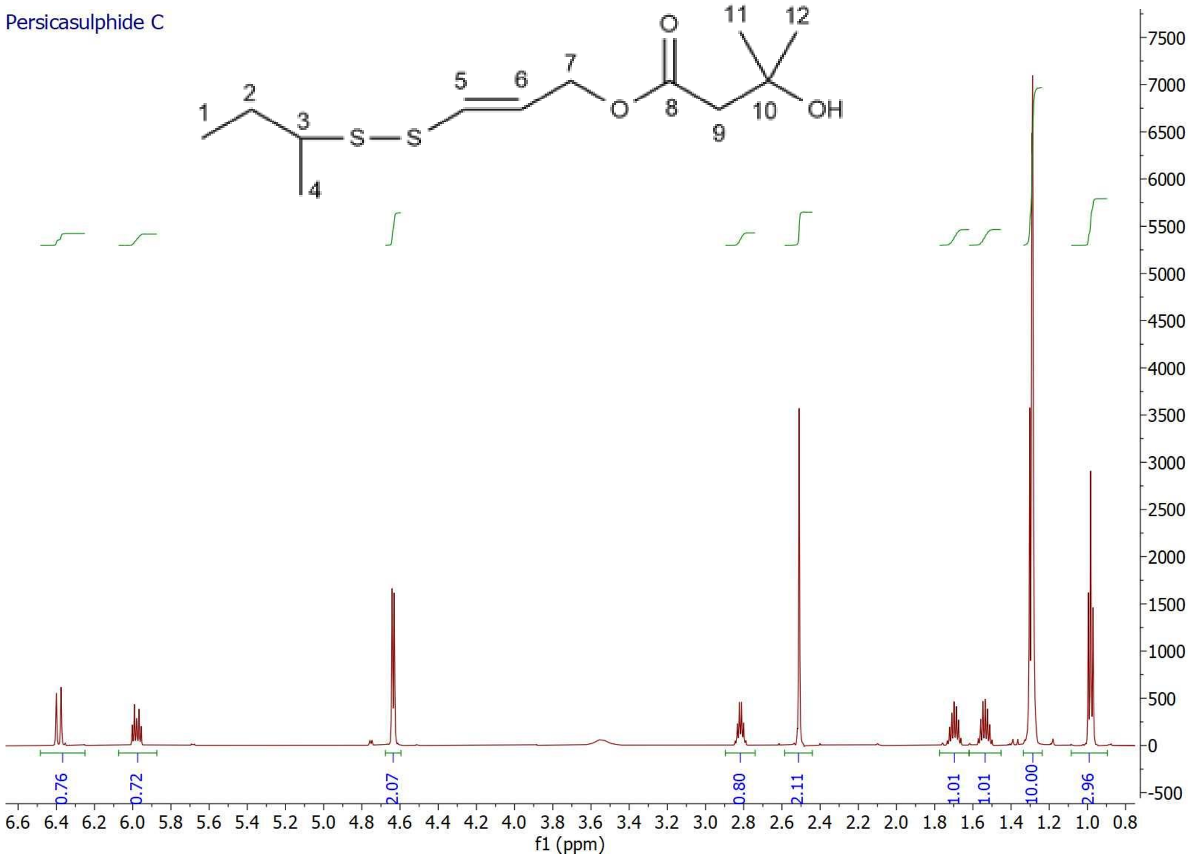

Figure S98 ^{1}H-NMR spectrum (600 MHz, $CDCl_3$) of persicasulphide C (**39**)

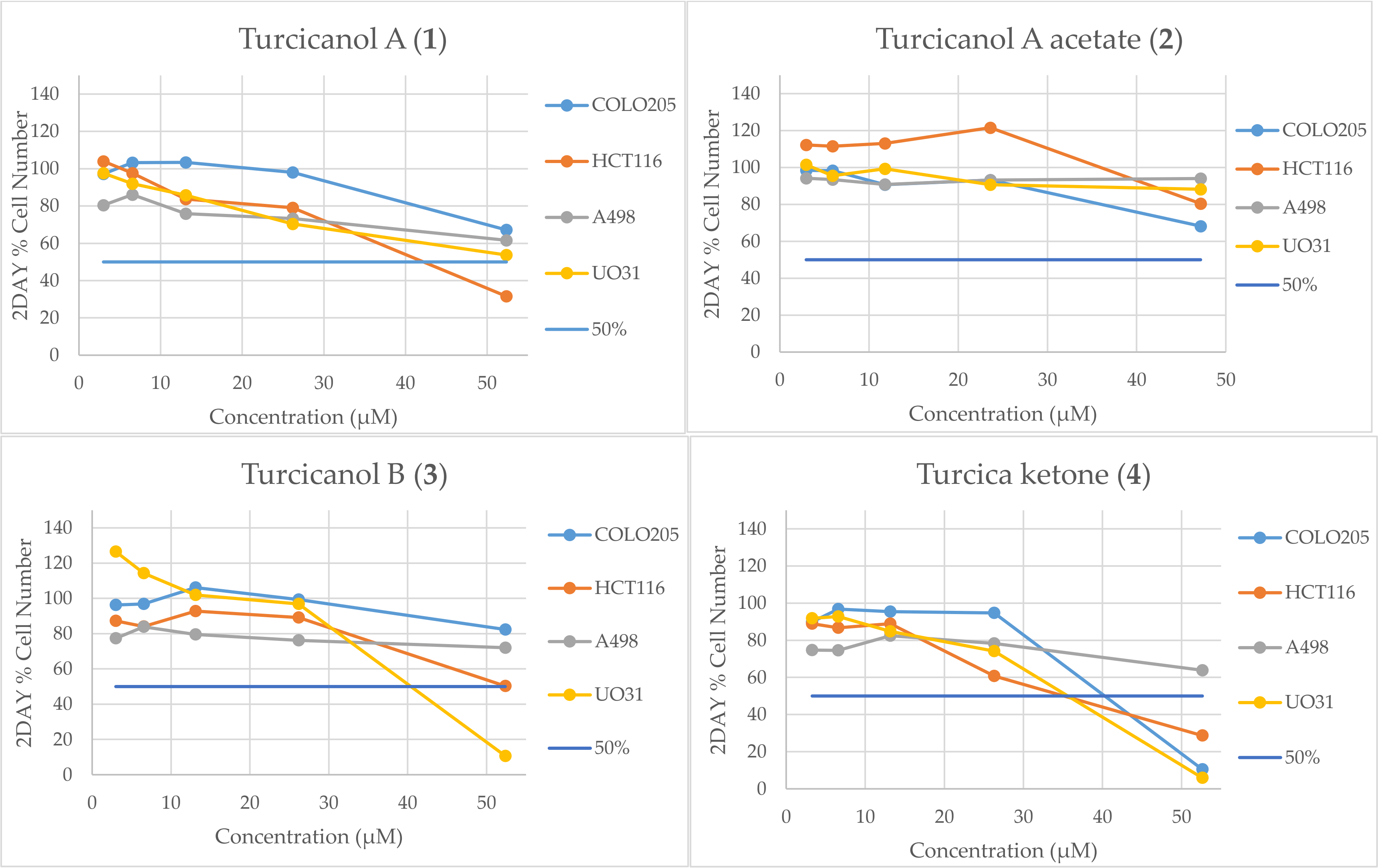

Figure S99 Cytotoxic activity graphics of pure compounds -1

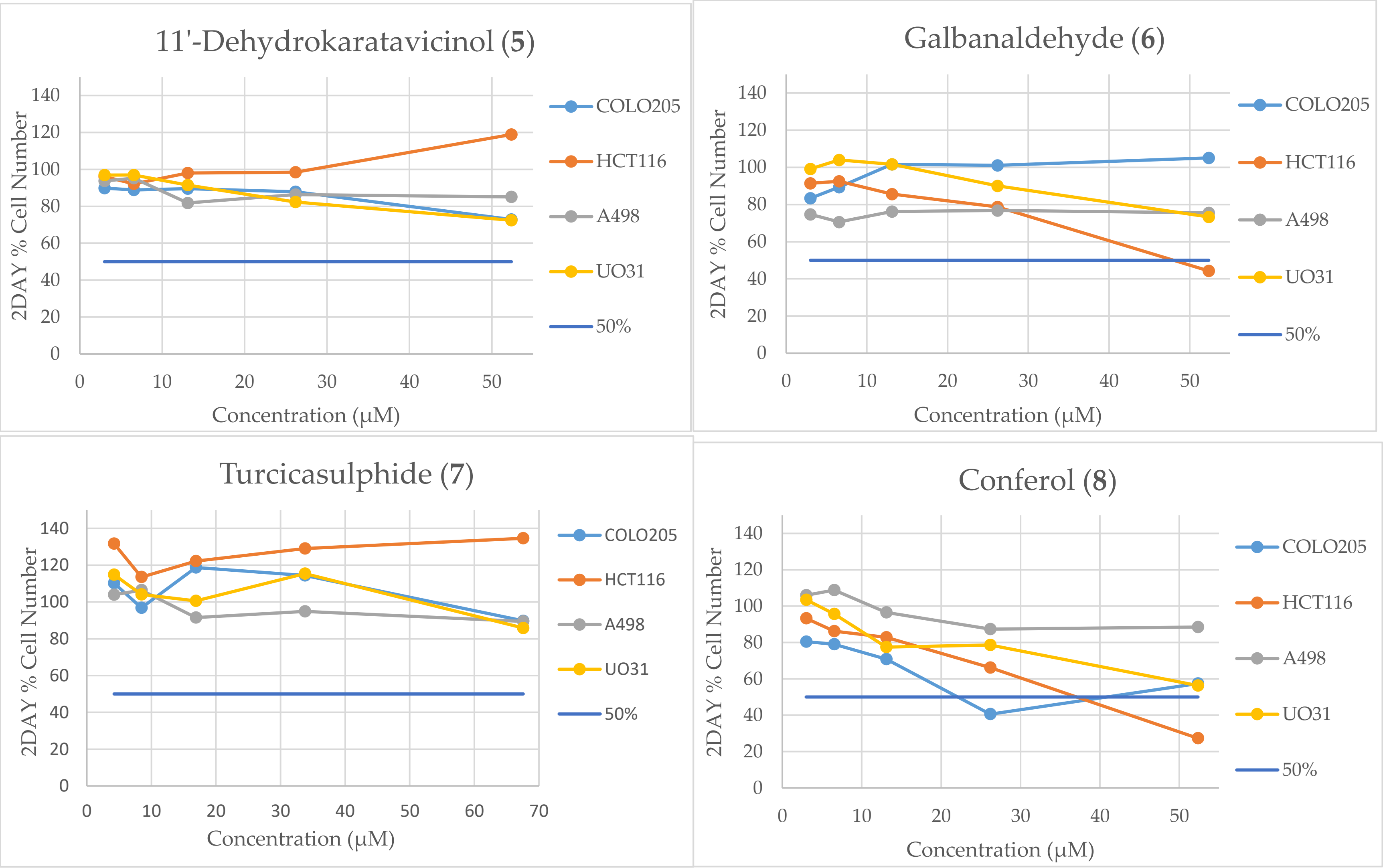

Figure S100 Cytotoxic activity graphics of pure compounds -2

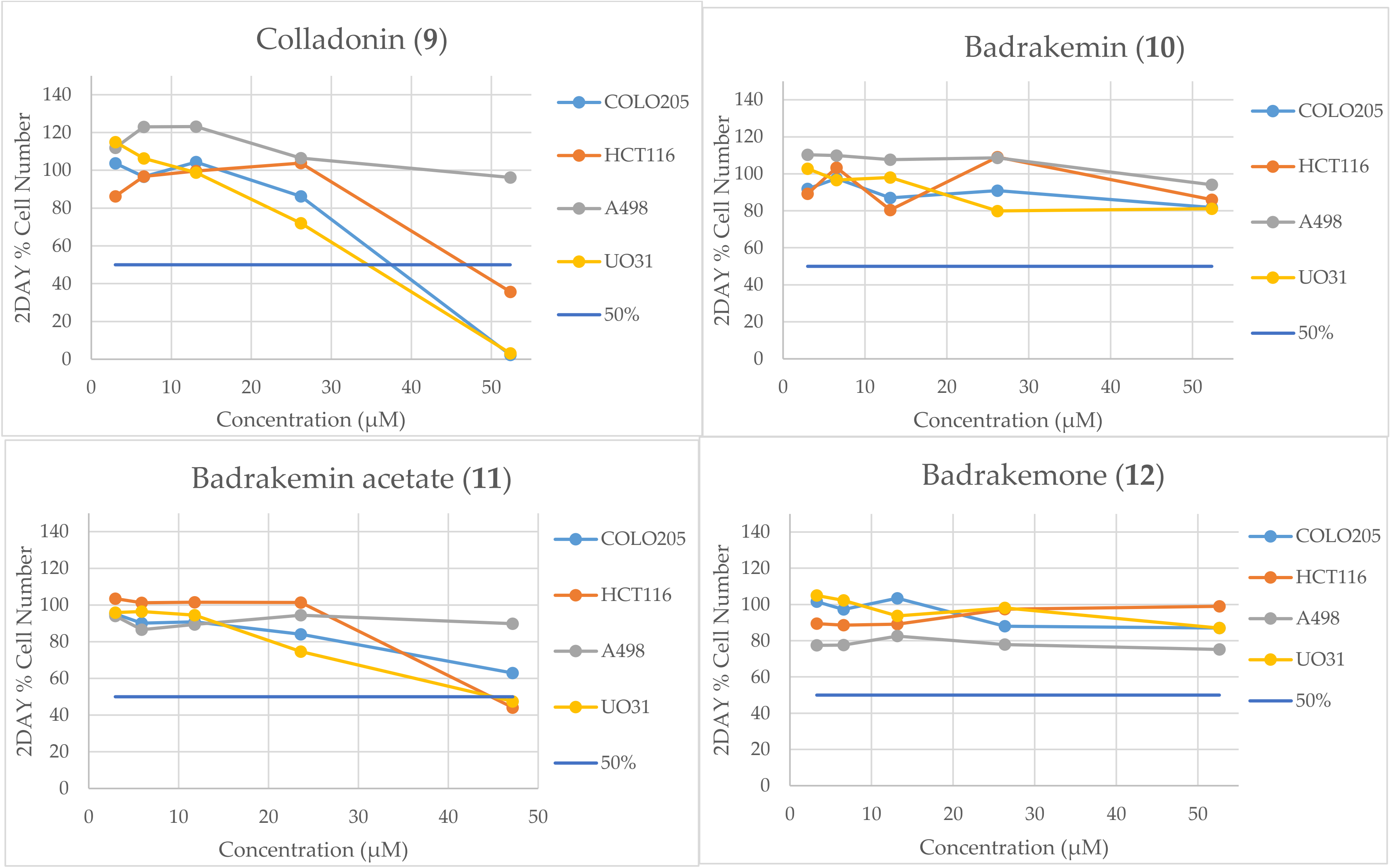

Figure S101 Cytotoxic activity graphics of pure compounds -3

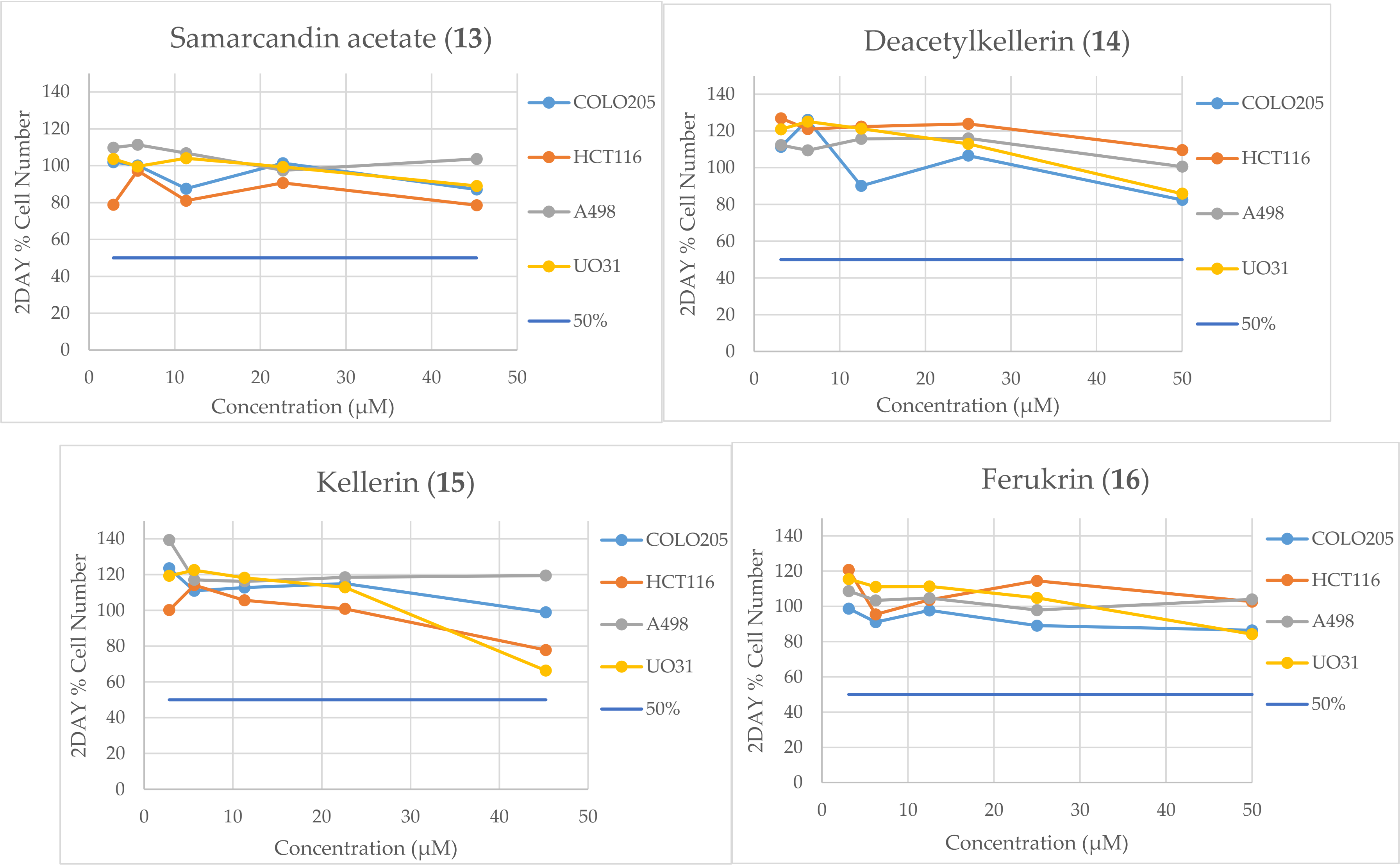

Figure S102 Cytotoxic activity graphics of pure compounds -4

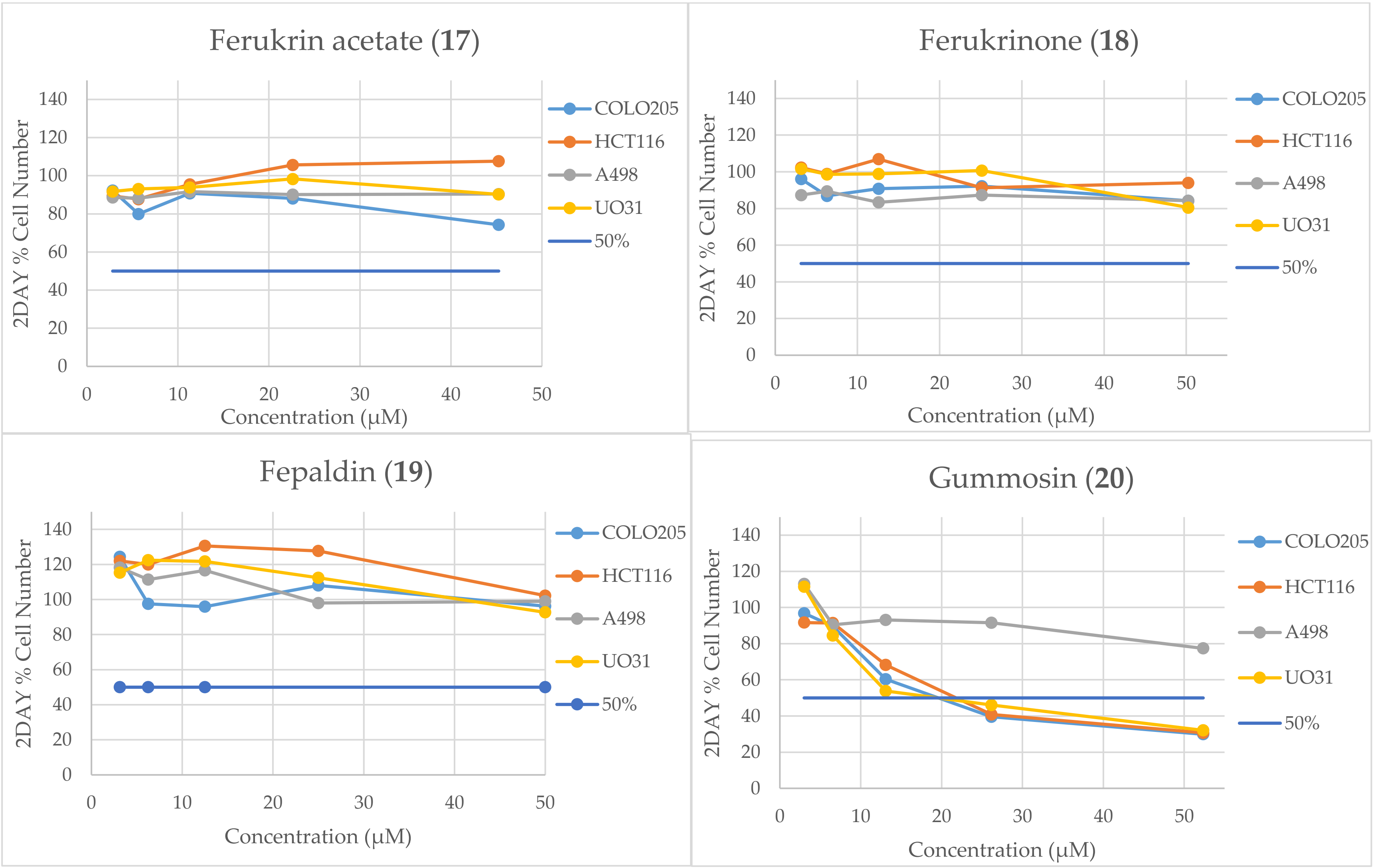

Figure S103 Cytotoxic activity graphics of pure compounds -5

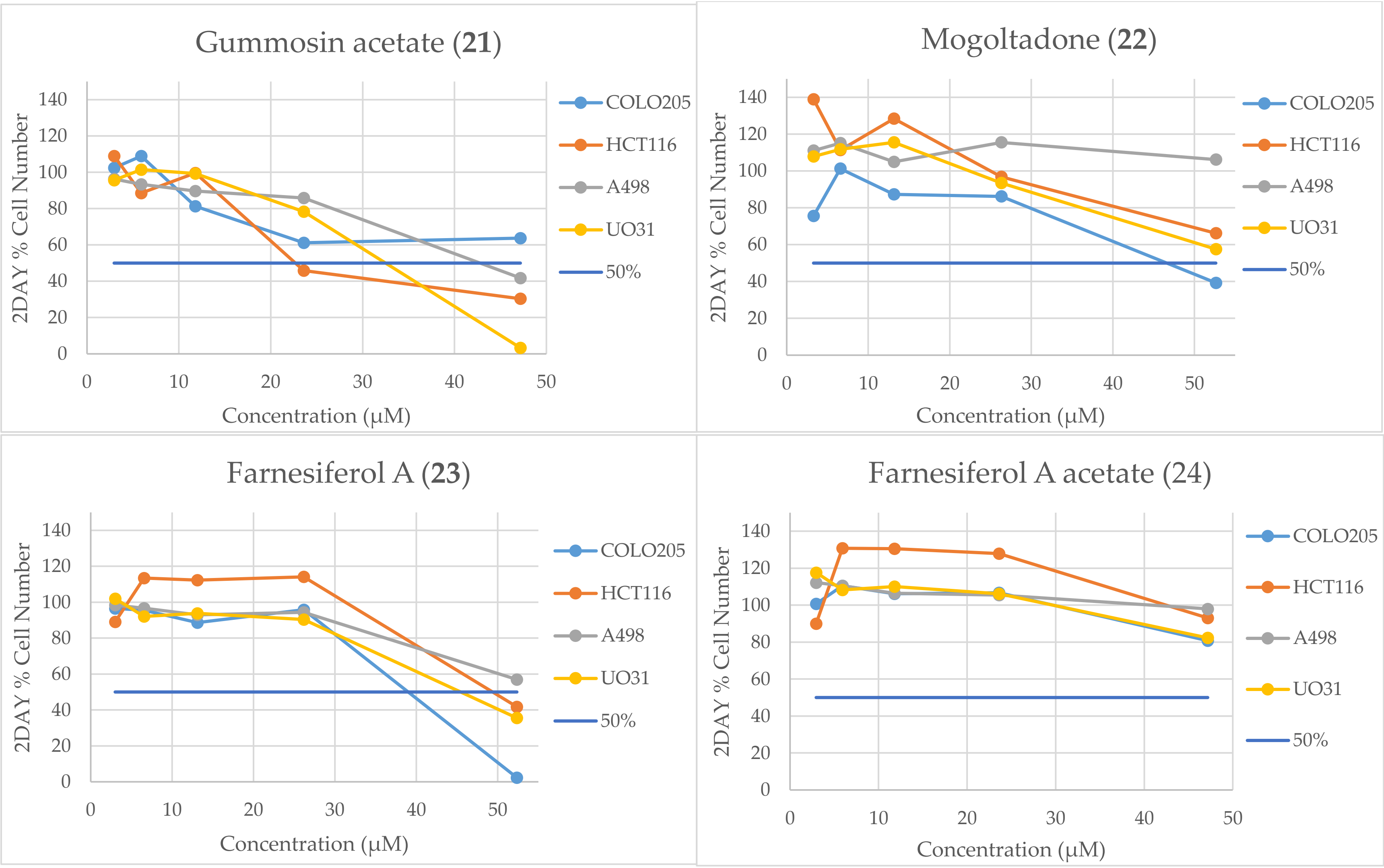

Figure S104 Cytotoxic activity graphics of pure compounds -6

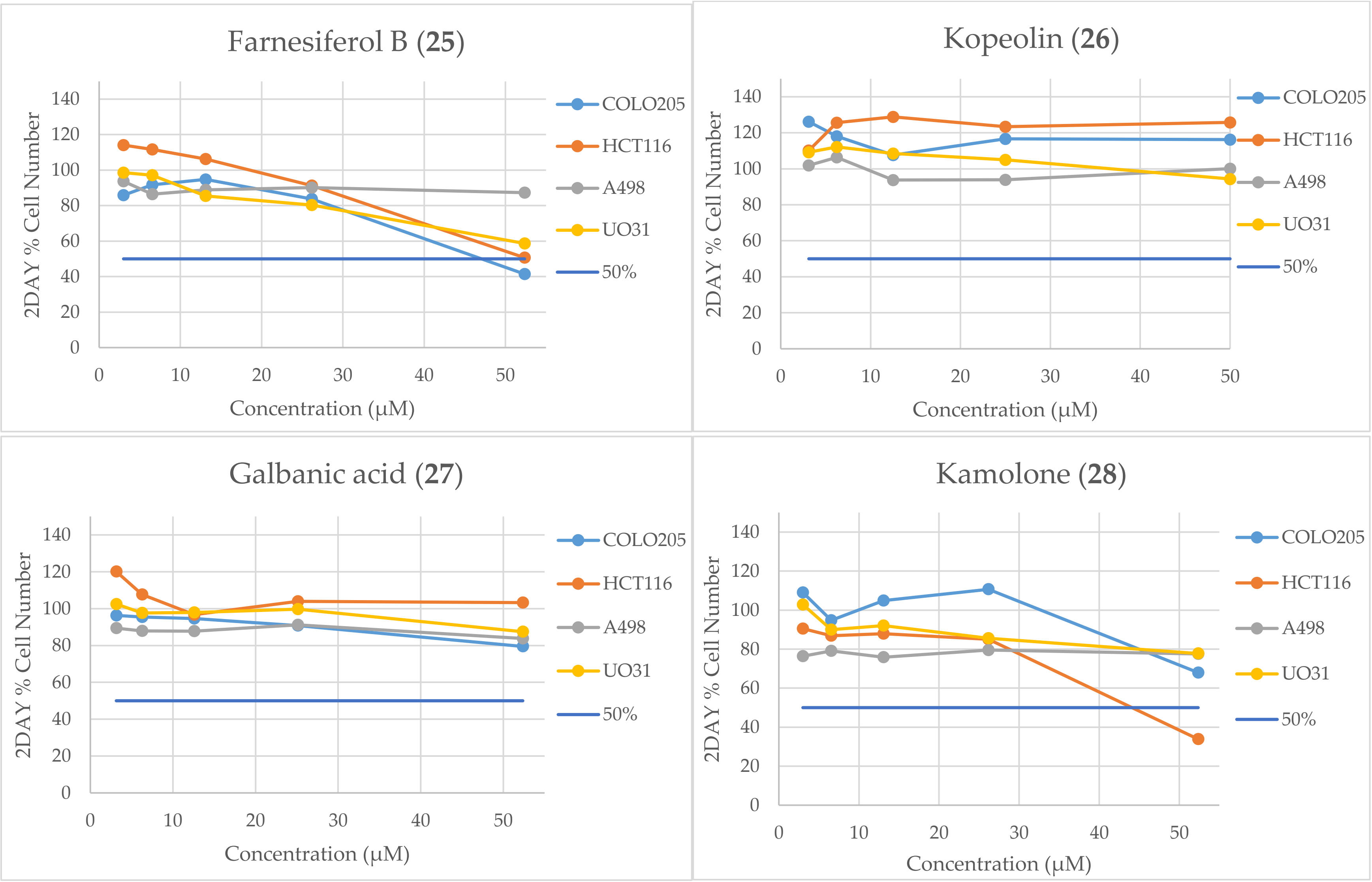

Figure S105 Cytotoxic activity graphics of pure compounds -7

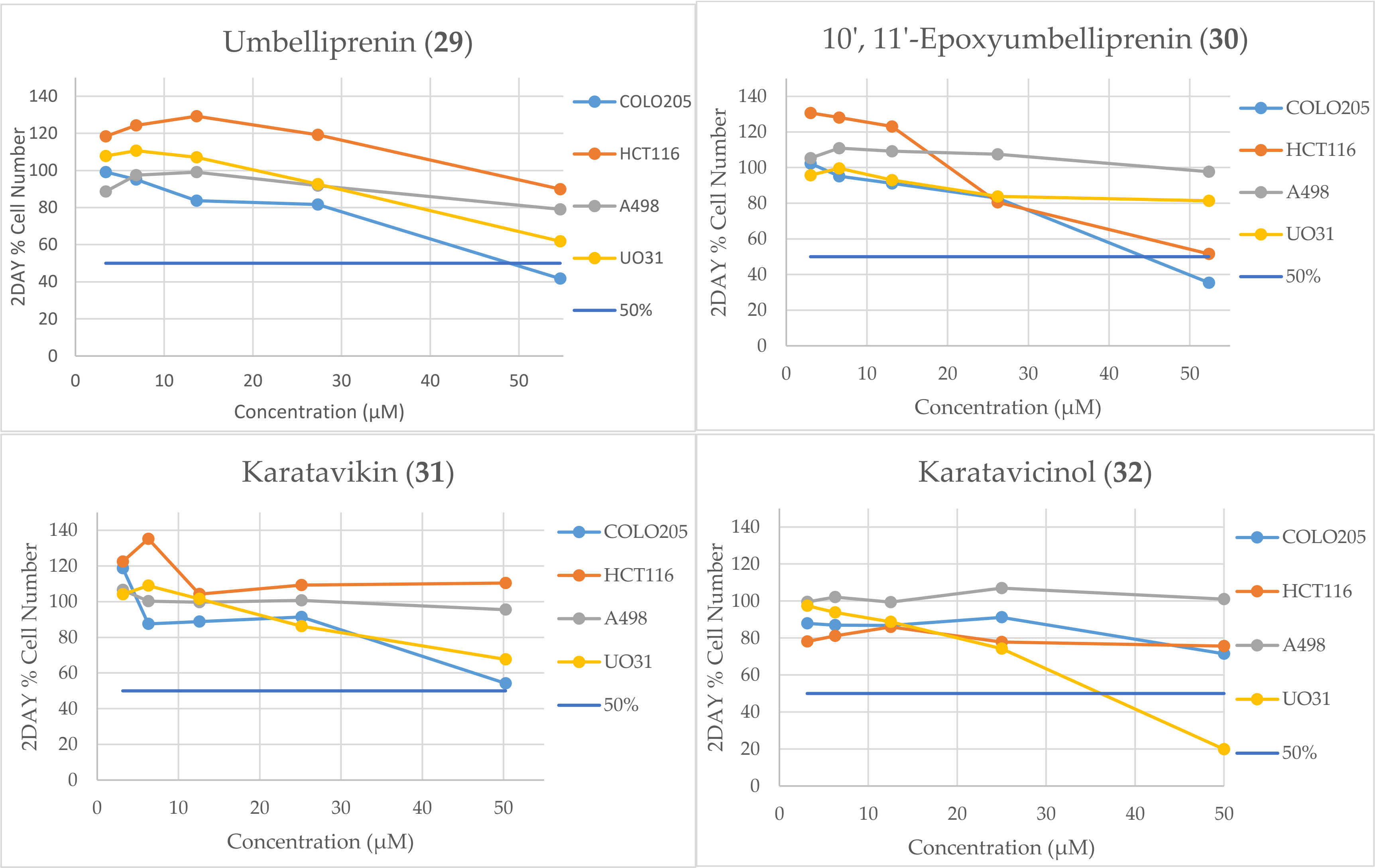

Figure S106 Cytotoxic activity graphics of pure compounds -8

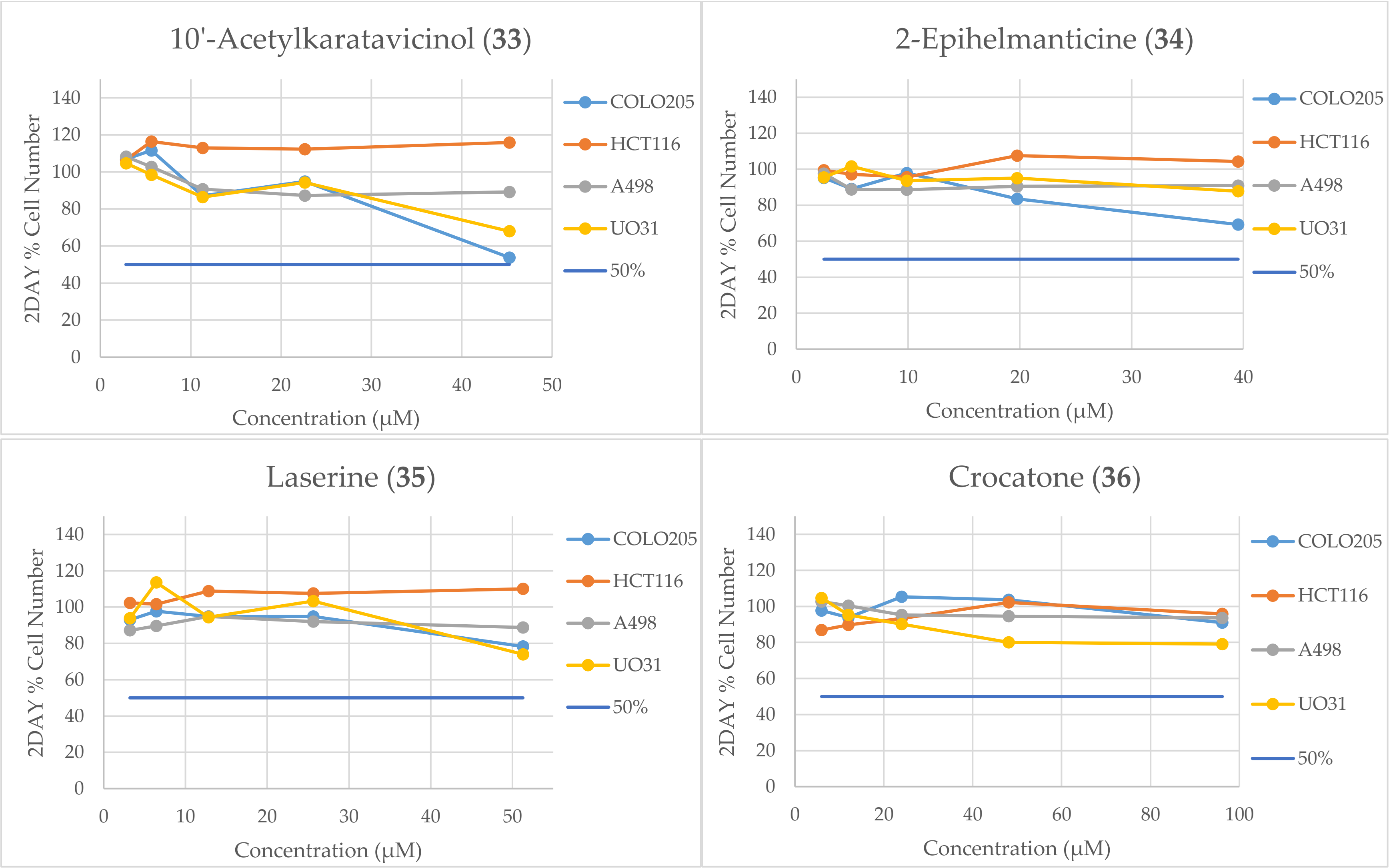

Figure S107 Cytotoxic activity graphics of pure compounds -9

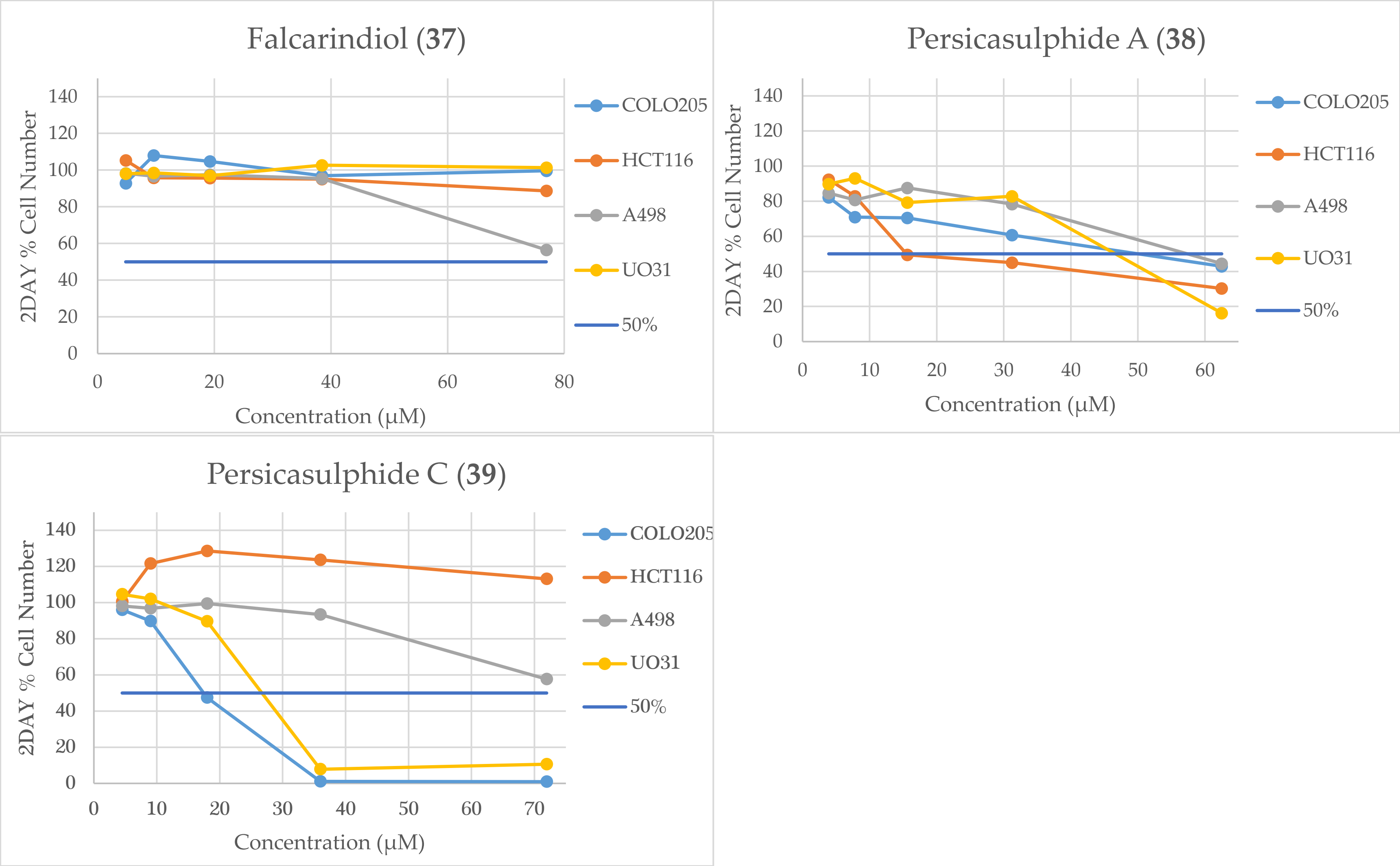

Figure S108 Cytotoxic activity graphics of pure compounds -10
